# Global impacts of COVID-19 on lifestyles and health and preparation preferences: An international survey of 30 countries

**DOI:** 10.7189/jogh.13.06031

**Published:** 2023-08-11

**Authors:** Jiaying Li, Daniel Yee Tak Fong, Kris Yuet Wan Lok, Janet Yuen Ha Wong, Mandy Man Ho, Edmond Pui Hang Choi, Vinciya Pandian, Patricia M Davidson, Wenjie Duan, Marie Tarrant, Jung Jae Lee, Chia-Chin Lin, Oluwadamilare Akingbade, Khalid M Alabdulwahhab, Mohammad Shakil Ahmad, Mohamed Alboraie, Meshari A Alzahrani, Anil S Bilimale, Sawitree Boonpatcharanon, Samuel Byiringiro, Muhammad Kamil Che Hasan, Luisa Clausi Schettini, Walter Corzo, Josephine M De Leon, Anjanette S De Leon, Hiba Deek, Fabio Efficace, Mayssah A El Nayal, Fathiya El-Raey, Eduardo Ensaldo-Carrasco, Pilar Escotorin, Oluwadamilola Agnes Fadodun, Israel Opeyemi Fawole, Yong-Shian Shawn Goh, Devi Irawan, Naimah Ebrahim Khan, Binu Koirala, Ashish Krishna, Cannas Kwok, Tung Thanh Le, Daniela Giambruno Leal, Miguel Ángel Lezana-Fernández, Emery Manirambona, Leandro Cruz Mantoani, Fernando Meneses-González, Iman Elmahdi Mohamed, Madeleine Mukeshimana, Chinh Thi Minh Nguyen, Huong Thi Thanh Nguyen, Khanh Thi Nguyen, Son Truong Nguyen, Mohd Said Nurumal, Aimable Nzabonimana, Nagla Abdelrahim Mohamed Ahmed Omer, Oluwabunmi Ogungbe, Angela Chiu Yin Poon, Areli Reséndiz-Rodriguez, Busayasachee Puang-Ngern, Ceryl G Sagun, Riyaz Ahmed Shaik, Nikhil Gauri Shankar, Kathrin Sommer, Edgardo Toro, Hanh Thi Hong Tran, Elvira L Urgel, Emmanuel Uwiringiyimana, Tita Vanichbuncha, Naglaa Youssef

**Affiliations:** 1 School of Nursing, Li Ka Shing Faculty of Medicine, University of Hong Kong, Hong Kong SAR, China; 2 School of Nursing and Health Studies, Hong Kong Metropolitan University, Hong Kong SAR, China; 3 School of Nursing, Johns Hopkins University, Baltimore, Maryland, USA; 4 Vice-Chancellor and Principal, University of Wollongong, Wollongong, Australia; 5 Department of Social Work, East China University of Science and Technology, Shanghai, China; 6 School of Nursing, The University of British Columbia, Kelowna British Columbia, Canada; 7 The Nethersole School of Nursing, The Chinese University of Hong Kong, Hong Kong; 8 Institute of Nursing Research, Osogbo, Osun State, Nigeria; 9 College of Medicine, Maimaah University, Al Majmaah, Saudi Arabia; 10 Department of Family & Community Medicine, College of Medicine, Majmaah University, Majmaah, Saudi Arabia.; 11 Department of Internal Medicine, Al-Azhar University, Cairo, Egypt; 12 Department of Urology, College of Medicine, Majmaah University, Al Majmaah, Saudi Arabia.; 13 School of Public Health, JSS Medical College, JSS AHER, Mysuru, India; 14 Department of Statistics, Chulalongkorn Business School, Bangkok, Thailand; 15 Kulliyyah of Nursing, International Islamic University, Kuantan, Malaysia; 16 Italian Association against Leukemia, Lymphoma and Myeloma (AIL) – Rome Section, Italy; 17 Diálogos Guatemala, Guatemala, Guatemala; 18 School of Nursing, Centro Escolar University, Manila, Philippines; 19 Nursing Department, Faculty of Health Science, Beirut Arab University, Lebanon; 20 Italian Group for Adult Hematologic Diseases (GIMEMA), Data Center and Health Outcomes Research Unit, Rome, Italy; 21 Department of Psychology, Beirut Arab University, Lebanon; 22 Department of hepatogastroenterology and infectious diseases, Damietta faculty of medicine, Al-Azher University, Egypt; 23 Ergonomics Research Center (ECR), University of Guadalajara, Jalisco, Mexico; 24 Laboratory of Applied Prosocial Research, Department of Basic, Developmental and Educational Psychology, Autonomous University of Barcelona, Spain; 25 Faculty of Health Sciences, University of Lethbridge, Alberta, Canada; 26 Faculty of Nursing, Ladoke Akintola University of Technology, Ogbomosho, Nigeria; 27 Alice Lee Centre for Nursing Studies, National University of Singapore, Singapore; 28 School of Nursing, Wijaya Husada Health Institute, Bogor, Indonesia; 29 Department of Optometry, University of Kwazulu-Natal, Durban, South Africa; 30 Ecove, Ghaziabad, India; 31 School of Nursing, Paramedicine and Health Care Science, Charles Sturt University, New South Wales, Australia; 32 Nam Dinh University of Nursing, Nam Dinh, Vietnam; 33 Pontificia Universidad Católica de Valparaíso, School of Social Work, Valparaíso, Chile; 34 Research Department, National Commission for Medical Arbitration, Mexico, Mexico; 35 College of Medicine and Health Sciences, University of Rwanda, Kigali, Rwanda; 36 Laboratory of Research in Respiratory Physiotherapy (LFIP), Department of Physiotherapy, State University of Londrina (UEL) – Londrina, Brazil; 37 Pharmacology and Toxicology Department, Faculty of Pharmacy, Benghazi University, Libya; 38 School of Nursing and Midwifery, College of Medicine and Health Sciences, University of Rwanda, Kigali, Rwanda; 39 Center for Language Enhancement, College of Arts and Social Sciences, University of Rwanda, Huye, Rwanda; 40 Faculty of Medicine, Alzaiem Alazhari University, Khartoum North, Sudan; 41 Faculty of Health Sciences and Sports, Macao Polytechnic University, Macao; 42 National Autonomous University of Mexico, Mexico; 43 Mental Health and Learning division, Wrexham Maelor Hospital, Wrexham, United Kingdom; 44 Medical-surgical Nursing Department, Faculty of Nursing, Cairo University, Egypt

## Abstract

**Background:**

The health area being greatest impacted by coronavirus disease 2019 (COVID-19) and residents’ perspective to better prepare for future pandemic remain unknown. We aimed to assess and make cross-country and cross-region comparisons of the global impacts of COVID-19 and preparation preferences of pandemic.

**Methods:**

We recruited adults in 30 countries covering all World Health Organization (WHO) regions from July 2020 to August 2021. 5 Likert-point scales were used to measure their perceived change in 32 aspects due to COVID-19 (-2 = substantially reduced to 2 = substantially increased) and perceived importance of 13 preparations (1 = not important to 5 = extremely important). Samples were stratified by age and gender in the corresponding countries. Multidimensional preference analysis displays disparities between 30 countries, WHO regions, economic development levels, and COVID-19 severity levels.

**Results:**

16 512 adults participated, with 10 351 females. Among 32 aspects of impact, the most affected were having a meal at home (mean (m) = 0.84, standard error (SE) = 0.01), cooking at home (m = 0.78, SE = 0.01), social activities (m = -0.68, SE = 0.01), duration of screen time (m = 0.67, SE = 0.01), and duration of sitting (m = 0.59, SE = 0.01). Alcohol (m = -0.36, SE = 0.01) and tobacco (m = -0.38, SE = 0.01) consumption declined moderately. Among 13 preparations, respondents rated medicine delivery (m = 3.50, SE = 0.01), getting prescribed medicine in a hospital visit / follow-up in a community pharmacy (m = 3.37, SE = 0.01), and online shopping (m = 3.33, SE = 0.02) as the most important. The multidimensional preference analysis showed the European Region, Region of the Americas, Western Pacific Region and countries with a high-income level or medium to high COVID-19 severity were more adversely impacted on sitting and screen time duration and social activities, whereas other regions and countries experienced more cooking and eating at home. Countries with a high-income level or medium to high COVID-19 severity reported higher perceived mental burden and emotional distress. Except for low- and lower-middle-income countries, medicine delivery was always prioritised.

**Conclusions:**

Global increasing sitting and screen time and limiting social activities deserve as much attention as mental health. Besides, the pandemic has ushered in a notable enhancement in lifestyle of home cooking and eating, while simultaneously reducing the consumption of tobacco and alcohol. A health care system and technological infrastructure that facilitate medicine delivery, medicine prescription, and online shopping are priorities for coping with future pandemics.

The coronavirus disease 2019 (COVID-19) pandemic has influenced the entire world, prompting more than 180 countries to adopt policy responses [1]. Both COVID-19 itself and the strict measures implemented to combat it have disrupted life and altered multifaceted lifestyle behaviours. Consequently, the pandemic has significantly impacted communities’ physical, psychological, financial, and social well-being. Individual health can be seriously compromised due to these impairments, burdening health care systems [2]. COVID-19's impact has been studied extensively, but evidence on the directions of various changes (e.g., smoking and drinking alcohol) remains inconsistent across countries [3,4]. For instance, while some studies have reported an increase in smoking among Chinese individuals, others have found a decrease in smoking among Italian and Spanish individuals. Similarly, some studies have reported a decrease in alcohol consumption among Spanish individuals, while others have found an increase in consumption among Russian individuals [3,4]. Moreover, using different designs and measurement tools across studies limits comparisons across global and impacted areas. Consequently, a multinational study is needed to measure disparities across countries. To date, three multinational studies have investigated COVID-19's impact on health: one focusing on Asia [5], another on Europe and Australia [6], and the other on 23 countries [7]. The latter study did not cover all of the World Health Organization (WHO) regions, such as the African region; and it only focused on mental health issues [7]. Thus, there is a need for a study that assesses COVID-19's impact on areas pertaining to health and lifestyles while utilising the same design and measurement tools across countries in all six WHO regions [8].

While it is our hope that the COVID-19 pandemic will end soon, we acknowledge that the path to normalcy is full of volatility, uncertainty, complexity, and ambiguity. Nevertheless, many countries have already shifted from imposing restrictive social measures with the aim of total eradication of COVID-19 to removing most measures and preparing to coexist with the virus. Indeed, we must be prepared to live with or minimise the societal impact of similar future pandemics. Protecting lives and minimising adverse impacts on society has been much sought, and health professionals from different disciplines have offered recommendations to improve health care systems [9,10]. However, there has been a lack of systematic assessments of needs from a societal perspective. As unmet needs can diminish life satisfaction, understanding people’s demands and priorities for future preparation is imperative. Moreover, it is uncertain whether needs differ across countries or regions.

Therefore, we aim to 1) globally assess the societal perceptions of COVID-19's impact and preferences for future preparations and 2) compare the impacts of COVID-19 and preparation preferences across countries, regions, economic levels, and COVID-19 severity levels. Specifically, we seek to answer the research question: How has COVID-19 impacted individuals’ lifestyles and health outcomes, and what are their preferences for future pandemic preparations? We hypothesise that: 1) the impact of COVID-19 would vary across different aspects of individuals' lifestyles and health outcomes, 2) different pandemic preparations would have varying levels of priority in individuals' preferences, and 3) the impact of COVID-19 and preferences for pandemic preparedness would differ across countries, regions, economic development levels, and COVID-19 severity levels. Our study information may assist health care organisations, governments, policymakers, social services, community activists, researchers, and other stakeholders in leveraging material and immaterial resources within a community to adapt to a pandemic.

## METHODS

### Study design

This was a cross-sectional international survey. The details of the study design, as well as the development, translation, and validation of the questionnaire were reported elsewhere [8].

### Setting

This study targeted populations from 30 countries: Australia, Brazil, Burundi, Canada, Chile, Egypt, Guatemala, Hong Kong, India, Indonesia, Italy, Lebanon, Libya, Macau, mainland China, Malaysia, Mexico, Nigeria, the Philippines, the Republic of Sudan, Rwanda, Saudi Arabia, Singapore, South Africa, South Korea, Spain, Thailand, the United Kingdom, the United States, and Vietnam. It covered six WHO regions, namely the Region of the Americas (AMR), the European Region (EUR), the Eastern Mediterranean Region (EMR), the South-East Asian Region (SEAR), the Western Pacific Region (WPR), and the African Region (AFR). Between July 6, 2020 and August 4, 2021, we recruited participants, primarily via tested online platforms, who self-completed the survey in their languages [8]. To motivate the study participation rate, one Hong Kong dollar (equivalent to 0.13 US dollars (US$)) was donated to the Red Cross for every completed questionnaire.

### Participants

This study employed convenient sampling with specific eligibility criteria that required participants to be adults aged 18 or above and possess the ability to complete the questionnaire in their respective language. The sample size calculation was based on estimating the prevalence of a health-related issue. Specifically, we used a conservative scenario of 50%, with a 5% margin of error and a 95% confidence interval. The sample size was calculated as 385 subjects in each participating country. To account for incomplete responses, we targeted to have 500 participants in each country.

### Variables and measurements

#### Socio-demographics

The sociodemographic variables included gender, age, country, marital status, education, employment, perceived social rank, weight, height, body mass index (BMI), weight status (based on the BMI thresholds of the corresponding populations [11]), pregnancy status, gestational week (if applicable), the need for regular medical follow-up before COVID-19, being a practicing health professional, having children under the age of 18, the number of people in the household, and house size.

#### Lifestyles and health-related impacts of COVID-19

Participants were asked to rate the degree of change in 32 lifestyle and health-related areas during COVID-19 when compared with those before the pandemic on a 5-point Likert scale (ranging from -2 = substantially reduced to 2 = substantially increased). Lifestyle and health-related areas included physical well-being (weight, appetite, and perceived physical health); psychological well-being (mental burden, emotional distress, sleep quality, quality of life, family disputes, social support provided, social support received, social activities); dietary (food types in daily meals, consumption of fruits and vegetables, consumption of frozen food / food products, consumption of snacks, drinking soft drinks, juices, or other sugary drinks, having a meal at home, cooking at home, eating takeout food, taking traditional Chinese medicine (TCM) or natural health products, taking oral supplements / vitamins); exercise (frequency, duration, type, and overall amount); sedentary behaviours (sitting and screen time duration); addiction behaviours (smoking tobacco and alcohol consumption); and financial situation (working hours, income, and economic burden).

#### Possible preparations

Participants were asked to rate, on a 5-point Likert scale (ranging from 1 = not important to 5 = extremely important), their perceived importance of 13 possible preparations during a pandemic, with higher scores indicating higher importance. These items included online consultations with doctors (e.g., Zoom, Skype), instant personalised health advice by online chatbots, telephone health advice, online courses, instant streaming courses (e.g., Zoom, Skype), receiving health information through e-mail, receiving health information through text (e.g., SMS, WhatsApp), receiving health information from social media (e.g., Facebook, Instagram, and Twitter), receiving health information from mobile apps, getting medicine prescribed during a hospital visit / follow-up in a community pharmacy, medicine delivery, online shopping, and food delivery.

#### Classification of countries by economic development level and COVID-19 severity

Based on the World Bank report for 2020 [12], we classified the economic development level of countries as low, lower-middle, upper-middle, and high. Moreover, for each country, we obtained the daily number of confirmed cases during the recruitment period from the WHO Coronavirus Disease dashboard [13]. Subsequently, we calculated the average daily percentage of population confirmed using the following formula: cumulative cases during the exact survey period in a country / (exact number of survey days in the country * total population in the country). Tertiles were used to classify the 30 countries as low, medium, and high COVID-19 severity.

#### Validation and rigor

To enhance internal validity, we administered the validation question of “Where does the sun rise every day?” This question was replaced with “Where is your STATE Capital?” in Nigeria for better cultural relevance. Furthermore, before a language-specific questionnaire or electronic survey platform was deployed, a pilot study involving at least ten respondents was conducted to ensure the data consistency across countries, and an adequate understanding of the items in the questionnaire. An expert panel comprising the local investigators and the principal investigator carefully reviewed the participants' responses. Overall, we did not find any significant inconsistencies or issues with comprehending the questionnaire. However, in Nigeria, participants found the validation question “Where does the sun rise every day?” to be awkward. After discussing with the local team, we replaced this question with the other validation question, “Where is your STATE Capital?”.

### Data collection

Data were collected via online survey platforms and offline electronic forms between July 2020 and August 2021. The participating countries used either online surveys developed in Qualtrics on the project website (https://care.hku.hk) or created their own links. An offline electronic form in PDF format was also created for places with limited Internet access so that the collected data could be electronically entered into a centralised database.

### Data analysis

The collected data were gathered into a master Excel database and cleaned by checking for missing responses, duplications, and inconsistencies. For each country, the sample weights were calculated based on the age and gender distribution of the corresponding population. Descriptive statistics were used to summarise the participants’ perception of COVID-19’s impact and the importance of possible preparations by country, WHO region, economic development, and COVID-19 severity levels. Specifically, continuous variables were assessed for normality using P-P plots and reported as mean and standard deviation, while categorical variables were reported as frequency and percentage. Their comparisons were assessed using multidimensional preference analyses weighted by each country's age and gender distribution. The number of dimensions was determined using the elbow method. Table S1 in the **Online Supplementary Document** shows the classifications of countries based on region, economic development, and pandemic severity levels. All analyses were performed using R Statistical Software (v4.1.1; R Core Team 2021).

## RESULTS

### Respondents’ socio-demographics

A total of 19 145 responses were received. After removing responses that were blank or 80% incomplete (n = 1940), duplicates (n = 116), inconsistent (n = 450), outside the 30 participating countries (n = 126), or lacking age or gender data (n = 1), we ended up with 16 512 responses. **Table 1** presents the detailed sociodemographic characteristics before and after weighting. Table S2 and S3 in the **Online Supplementary Document** show the distribution of respondents' unweighted and weighted socio-demographics for each country or region, respectively.

**Table 1:** Demographics and characteristics of 16 512 respondents

Variables	Unweighted (n = 16 512)	Weighted (n = 16 280)
**Variables, n (%)**		
Gender		
*Female*	10 351 (62.7%)	8171 (50.2%)
*Male*	6061 (36.7%)	8000 (49.1%)
*Non-binary*	100 (0.6%)	109 (0.7%)
Age		
*18-24 y-old*	4857 (29.4%)	1994 (12.3%)
*25-29 y-old*	2345 (14.2%)	1968 (12.1%)
*30-34 y-old*	1931 (11.7%)	1877 (11.5%)
*35-39 y-old*	1855 (11.2%)	1824 (11.2%)
*40-44 y-old*	1427 (8.6%)	1646 (10.1%)
*45-49 y-old*	1157 (7.0%)	1575 (9.7%)
*50-54 y-old*	975 (5.9%)	1388 (8.5%)
*55-59 y-old*	667 (4.0%)	1244 (7.6%)
*60-64 y-old*	699 (4.2%)	869 (5.3%)
*> = 65 y-old*	599 (3.6%)	1894 (11.6%)
Country		
*Australia*	639 (3.9%)	639 (3.9%)
*Brazil*	553 (3.3%)	553 (3.4%)
*Burundi*	369 (2.2%)	369 (2.3%)
*Canada*	368 (2.2%)	368 (2.3%)
*Chile*	342 (2.1%)	342 (2.1%)
*Egypt*	461 (2.8%)	461 (2.8%)
*Guatemala*	229 (1.4%)	229 (1.4%)
*Hong Kong*	2127 (12.9%)	2127 (13.1%)
*India*	529 (3.2%)	529 (3.2%)
*Indonesia*	482 (2.9%)	405 (2.5%)
*Italy*	203 (1.2%)	203 (1.2%)
*Lebanon*	440 (2.7%)	440 (2.7%)
*Libya*	645 (3.9%)	612 (3.8%)
*Macau*	250 (1.5%)	233 (1.4%)
*Mainland China*	667 (4.0%)	667 (4.1%)
*Malaysia*	535 (3.2%)	535 (3.3%)
*Mexico*	1016 (6.2%)	1016 (6.2%)
*Nigeria*	590 (3.6%)	580 (3.6%)
*Philippines*	457 (2.8%)	457 (2.8%)
*Republic of Sudan*	538 (3.3%)	538 (3.3%)
*Rwanda*	150 (0.9%)	136 (0.8%)
*Saudi Arabia*	631 (3.8%)	609 (3.7%)
*Singapore*	237 (1.4%)	237 (1.5%)
*South Africa*	198 (1.2%)	192 (1.2%)
*South Korea*	2238 (13.6%)	2238 (13.7%)
*Spain*	51 (0.3%)	45 (0.3%)
*Thailand*	723 (4.4%)	723 (4.4%)
*United Kingdom*	212 (1.3%)	212 (1.3%)
*United States*	213 (1.3%)	184 (1.1%)
*Vietnam*	419 (2.5%)	401 (2.5%)
Marital status		
*Married / cohabitation / common-law*	7275 (44.1%)	9442 (58.0%)
*Single*	8504 (51.5%)	5645 (34.7%)
*Separated / divorced / widowed*	732 (4.4%)	1193 (7.3%)
*Missing data*	1 (0.0%)	1 (0.0%)
Education		
*Primary or below*	405 (2.5%)	729 (4.5%)
*Secondary*	2627 (15.9%)	2410 (14.8%)
*Associate degree*	1576 (9.5%)	1339 (8.2%)
*Bachelor*	6500 (39.4%)	5393 (33.1%)
*College*	2258 (13.7%)	2271 (13.9%)
*Graduate*	2974 (18.0%)	3976 (24.4%)
*Missing*	172 (1.0%)	162 (1.0%)
Employment		
*Job seeking*	885 (5.4%)	747 (4.6%)
*Laid off*	170 (1.0%)	197 (1.2%)
*Not in workforce*	990 (6.0%)	1233 (7.6%)
*Retired*	614 (3.7%)	1447 (8.9%)
*Self-employed*	1309 (7.9%)	1672 (10.3%)
*Student*	4589 (27.8%)	2103 (12.9%)
*Working (> = 40 h/wk)*	5196 (31.5%)	5683 (34.9%)
*Working (1-39 h/wk)*	2759 (0.1671	3198 (19.6%)
BMI classification		
*Underweight*	1208 (19.5%)	744 (4.5%)
*Normal weight*	7456 (45.4%)	6293 (38.7%)
*Overweight*	3779 (23.0%)	4315 (26.5%)
*Obese*	3972 (24.2%)	4797 (29.5%)
*Missing data*	97 (0.6%)	132 (0.8%)
Pregnant		
*Yes*	226 (1.4%)	283 (1.7%)
*No*	10 179 (61.7%)	7966 (48.9%)
*Not applicable*	6107 (37.0%)	8031 (49.3%)
The need for regular medical follow-up before COVID-19		
*Yes*	4951 (30.0%)	6117 (37.6%)
*No*	11 558 (70.0%)	10 160 (62.4%)
*Missing data*	3 (0.0%)	3 (0.0%)
Practicing health professional		
*Yes*	4145 (25.1%)	3922 (24.1%)
*No*	12 366 (74.9%)	12 358 (75.9%)
*Missing data*	1 (0.0%)	0 (0.0%)
Having children less than 18 y of age		
*Yes*	4667 (28.3%)	5369 (33.0%)
*No*	11 845 (71.7%)	10 911 (67.0%)
**Variabels, mean (standard deviation)**		
Perceived social rank, 1 = lowest to 5 = highest	3.11 (0.9)	3.13 (0.92)
Weight, kg	65.62 (14.97)	68.45 (14.93)
Height, m	1.65 (0.09)	1.66 (0.10)
BMI, kg / m^2^	24.06 (4.70)	24.84 (4.71)
Gestational week	19.77 (12.9%)	23.10 (14.16)
Number of children less than 18 y old	0.50 (0.96)	0.62 (1.08)
Number of people in the household	3.94 (2.04)	3.78 (2.01)
House size, m^2^	106.70 (107.55)	109.74 (107.72)

y – years, hrs – hours, wk – week, BMI – body mass index, kg – kilogrammes, m – metres

### Respondents’ perception of COVID-19’s impact

**Figure 1:**
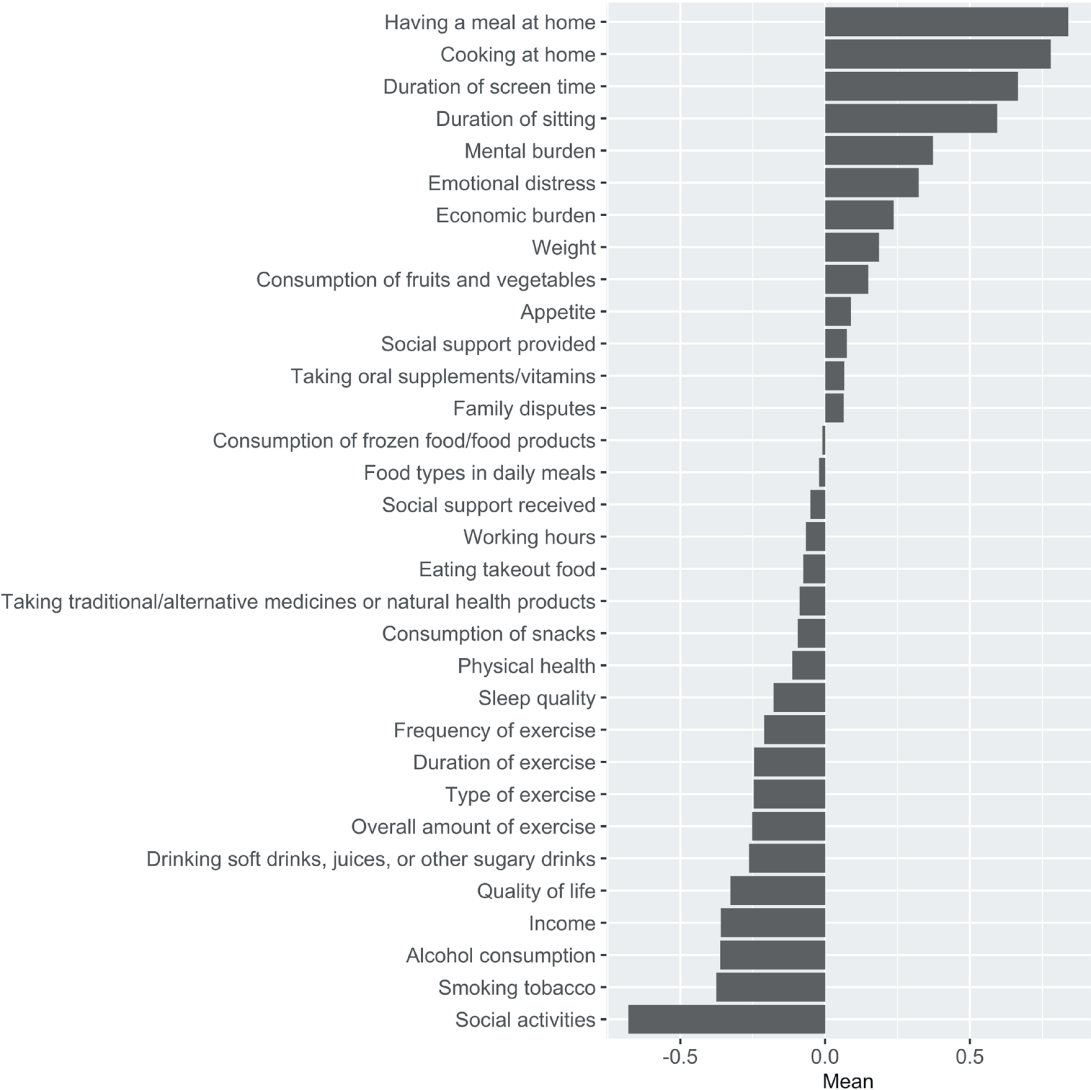
Overall weighted mean of COVID-19’s impact. The ratings of -2, -1, 0, 1, and 2 represent substantially reduced, a bit reduced, no change, a bit increased, and substantially increased, respectively.

**Table 2** summarises the perceived changes in lifestyles and health-related areas by WHO region, economic development, and COVID-19 severity level. The corresponding by-country summary is provided in Table S4 in the **Online Supplementary Document** and visualised in Figure S1 in the **Online Supplementary Document.** **Figure 1** depicts the overall weighted mean of COVID-19’s impact. Compared with the pre-pandemic period, the top five changes were more frequently having a meal at home (m = 0.84, standard deviation (SD) = 1.00), cooking more frequently at home (m = 0.78, SD = 1.00), reduced social activities (m = -0.68, SD = 1.06), longer screen time duration (m = 0.67, SD = 0.97), and longer duration of sitting (m = 0.59, SD = 0.97) (**Figure 1**). Remarkably, respondents also experienced a reduction in tobacco use (m = -0.38, SD = 0.95) and alcohol consumption (m = -0.36, SD = 0.95).

**Table 2:** Weighted mean (standard deviation) of perceived impact of COVID-19 on lifestyles and health-related areas by World Health Organization (WHO) region, economic development level, and COVID-19 severity level

Impact	World Health Organization regions						Economic development levels				COVID-19 severity levels		
	**AFR**	**AMR**	**EMR**	**EUR**	**SEAR**	**WPR**	**High**	**Upper-Middle**	**Lower-Middle**	**Low**	**High**	**Medium**	**Low**
**Number of countries**	4	6	5	3	3	9	12	9	6	3	10	10	10
**Number of participants**	1307	2721	2715	466	1734	7569	7511	5006	2938	1057	3688	4341	8483
**Lifestyles impact (-2 = substantially reduced to 2 = substantially increased)**													
1. Food types in daily meals	-0.25 (1.00)	0.16 (0.93)	-0.07 (0.89)	0.12 (0.75)	-0.09 (0.87)	-0.02 (0.79)	0.00 (0.78)	-0.01 (0.90)	0.00 (0.92)	-0.28 (1.00)	0.06 (0.90)	0.01 (0.94)	-0.07 (0.80)
2. Consumption of fruits and vegetables	0.02 (1.11)	0.24 (0.92)	0.24 (0.94)	0.28 (0.74)	0.11 (0.85)	0.11 (0.82)	0.12 (0.81)	0.17 (0.90)	0.25 (0.97)	0.01 (1.06)	0.24 (0.91)	0.20 (0.93)	0.08 (0.85)
3. Consumption of frozen food / food products	-0.37 (1.00)	-0.05 (0.96)	-0.40 (0.97)	0.03 (0.86)	-0.25 (0.96)	0.26 (0.87)	0.29 (0.83)	-0.22 (0.97)	-0.25 (1.03)	-0.48 (0.96)	-0.12 (0.93)	-0.15 (0.99)	0.11 (0.94)
4. Consumption of snacks	-0.19 (1.07)	-0.13 (1.16)	-0.41 (1.05)	0.04 (1.04)	-0.24 (0.99)	0.07 (0.86)	0.10 (0.90)	-0.24 (1.06)	-0.25 (1.02)	-0.35 (1.01)	-0.15 (1.14)	-0.20 (1.05)	-0.02 (0.90)
5. Soft drinks / juices / other sugary drinks	-0.36 (1.10)	-0.34 (1.11)	-0.69 (1.10)	-0.13 (0.92)	-0.36 (1.05)	-0.06 (0.92)	-0.05 (0.92)	-0.41 (1.10)	-0.46 (1.10)	-0.56 (1.06)	-0.41 (1.08)	-0.42 (1.13)	-0.13 (0.95)
6. Having meal at home	0.48 (1.11)	1.11 (0.99)	0.67 (1.10)	0.67 (0.95)	0.62 (1.02)	0.93 (0.89)	0.97 (0.87)	0.88 (1.05)	0.58 (1.04)	0.41 (1.14)	0.87 (1.05)	0.77 (1.08)	0.86 (0.93)
7. Cooking at home	0.46 (1.12)	0.99 (1.02)	0.66 (1.09)	0.58 (1.11)	0.50 (1.04)	0.87 (0.87)	0.91 (0.87)	0.77 (1.06)	0.56 (1.08)	0.45 (1.13)	0.78 (1.06)	0.75 (1.09)	0.79 (0.92)
8. Eating takeout food	-0.46 (1.11)	-0.01 (1.24)	-0.83 (1.10)	-0.10 (1.13)	-0.03 (1.17)	0.22 (1.08)	0.24 (1.08)	-0.24 (1.23)	-0.40 (1.19)	-0.68 (1.03)	-0.32 (1.23)	-0.31 (1.20)	0.15 (1.11)
9. Taking alternative medicine or natural health products	-0.16 (1.06)	0.20 (0.88)	-0.11 (1.16)	-0.15 (0.84)	-0.37 (0.99)	-0.10 (0.71)	-0.07 (0.72)	-0.06 (1.01)	-0.26 (1.02)	0.09 (1.09)	0.06 (0.95)	-0.03 (1.04)	-0.18 (0.79)
10. Taking oral supplements / vitamins	0.00 (1.06)	0.35 (0.91)	-0.22 (1.16)	0.12 (0.88)	-0.11 (1.02)	0.11 (0.77)	0.14 (0.76)	0.06 (1.04)	-0.05 (1.08)	-0.14 (1.06)	0.14 (1.01)	0.08 (1.03)	0.03 (0.84)
11. Smoking tobacco	-0.47 (0.97)	-0.38 (0.96)	-0.67 (1.12)	-0.21 (0.78)	-0.53 (0.98)	-0.23 (0.84)	-0.18 (0.81)	-0.52 (1.01)	-0.61 (1.08)	-0.42 (0.92)	-0.42 (1.00)	-0.46 (1.01)	-0.31 (0.89)
12. Alcohol consumption	-0.42 (1.03)	-0.17 (1.01)	-0.84 (1.01)	-0.20 (0.90)	-0.44 (0.99)	-0.28 (0.85)	-0.18 (0.83)	-0.49 (0.98)	-0.60 (1.08)	-0.35 (1.00)	-0.37 (1.03)	-0.36 (1.00)	-0.36 (0.90)
13. Duration of sitting	0.35 (1.12)	1.02 (0.96)	0.39 (1.09)	0.88 (0.88)	0.29 (0.94)	0.61 (0.84)	0.73 (0.84)	0.67 (1.01)	0.23 (1.04)	0.30 (1.08)	0.83 (1.01)	0.55 (1.05)	0.52 (0.89)
14. Duration of screen time	0.36 (1.14)	1.10 (0.95)	0.35 (1.10)	1.01 (0.78)	0.41 (0.90)	0.71 (0.83)	0.80 (0.81)	0.75 (1.03)	0.31 (1.02)	0.23 (1.12)	0.85 (1.02)	0.61 (1.06)	0.61 (0.88)
15. Frequency of exercise	0.08 (1.06)	-0.37 (1.25)	-0.30 (1.09)	-0.30 (1.20)	-0.09 (0.92)	-0.19 (0.98)	-0.27 (1.01)	-0.22 (1.14)	-0.07 (1.04)	-0.09 (1.08)	-0.32 (1.18)	-0.14 (1.10)	-0.20 (0.98)
16. Duration of exercise	-0.02 (1.09)	-0.40 (1.23)	-0.38 (1.08)	-0.34 (1.16)	-0.12 (0.87)	-0.20 (0.96)	-0.29 (0.98)	-0.26 (1.11)	-0.09 (1.05)	-0.24 (1.09)	-0.38 (1.16)	-0.16 (1.08)	-0.23 (0.96)
17. Type of exercise	0.07 (1.03)	-0.36 (1.22)	-0.36 (1.04)	-0.30 (1.13)	-0.08 (0.86)	-0.25 (0.90)	-0.31 (0.93)	-0.25 (1.10)	-0.11 (0.98)	-0.14 (1.03)	-0.34 (1.15)	-0.18 (1.03)	-0.24 (0.92)
18. Overall amount of exercise	0.07 (1.06)	-0.42 (1.26)	-0.34 (1.06)	-0.31 (1.25)	-0.13 (0.90)	-0.24 (0.96)	-0.32 (1.00)	-0.27 (1.12)	-0.09 (1.03)	-0.12 (1.04)	-0.37 (1.20)	-0.17 (1.08)	-0.24 (0.96)
**Health-related impact (-2 = substantially reduced to 2 = substantially increased)**													
19. Weight	-0.04 (1.00)	0.26 (1.03)	0.04 (0.94)	0.31 (0.91)	0.07 (0.80)	0.27 (0.76)	0.29 (0.79)	0.18 (0.94)	0.05 (0.90)	-0.09 (0.99)	0.20 (1.00)	0.11 (0.95)	0.22 (0.77)
20. Appetite	0.14 (0.89)	0.26 (0.89)	-0.03 (0.96)	0.13 (0.78)	0.05 (0.71)	0.07 (0.66)	0.10 (0.70)	0.11 (0.88)	0.04 (0.81)	0.09 (0.90)	0.13 (0.94)	0.11 (0.86)	0.06 (0.67)
21. Physical health	0.12 (0.93)	-0.16 (0.94)	-0.19 (0.84)	-0.24 (0.80)	0.05 (0.73)	-0.14 (0.66)	-0.19 (0.69)	-0.09 (0.84)	0.05 (0.85)	-0.10 (0.85)	-0.19 (0.89)	-0.04 (0.84)	-0.12 (0.69)
22. Sleep quality	0.07 (0.99)	-0.42 (1.06)	-0.26 (1.03)	-0.33 (0.95)	0.11 (0.82)	-0.16 (0.77)	-0.25 (0.80)	-0.20 (1.01)	0.00 (0.95)	-0.06 (0.98)	-0.36 (1.05)	-0.09 (0.98)	-0.15 (0.79)
23. Quality of life	-0.08 (1.02)	-0.39 (1.06)	-0.45 (1.05)	-0.57 (1.03)	0.09 (0.86)	-0.38 (0.83)	-0.49 (0.87)	-0.24 (1.00)	-0.06 (0.96)	-0.34 (0.98)	-0.48 (1.04)	-0.15 (0.99)	-0.35 (0.86)
24. Mental burden	0.25 (1.09)	0.92 (1.04)	0.25 (1.17)	0.70 (1.00)	0.21 (0.88)	0.26 (0.96)	0.35 (1.02)	0.55 (1.07)	0.15 (1.00)	0.26 (1.11)	0.68 (1.11)	0.35 (1.07)	0.25 (0.98)
25. Emotional distress	0.32 (1.11)	0.97 (0.98)	0.04 (1.08)	0.62 (0.87)	0.17 (0.90)	0.21 (0.92)	0.29 (0.95)	0.52 (1.06)	0.08 (1.00)	0.23 (1.11)	0.63 (1.07)	0.32 (1.04)	0.19 (0.95)
26. Family disputes	0.04 (1.08)	0.22 (0.89)	-0.02 (1.03)	0.13 (0.72)	-0.11 (0.81)	0.08 (0.71)	0.12 (0.71)	0.11 (0.91)	-0.16 (0.93)	0.11 (1.07)	0.17 (0.93)	-0.01 (0.88)	0.05 (0.79)
27. Social support provided	0.07 (1.07)	0.17 (1.03)	0.28 (1.03)	0.10 (0.82)	0.05 (0.78)	-0.03 (0.70)	0.01 (0.75)	0.18 (0.94)	0.02 (0.93)	0.24 (1.11)	0.17 (1.02)	0.15 (0.92)	-0.01 (0.77)
28. Social support received	-0.11 (1.05)	-0.17 (0.93)	-0.18 (0.99)	-0.06 (0.76)	-0.10 (0.79)	0.06 (0.71)	0.02 (0.73)	-0.12 (0.90)	-0.12 (0.91)	-0.04 (1.05)	-0.20 (0.94)	-0.03 (0.88)	0.01 (0.77)
29. Social activities	-0.42 (1.13)	-1.27 (1.04)	-0.55 (1.10)	-1.15 (1.07)	-0.09 (1.11)	-0.66 (0.91)	-0.83 (0.93)	-0.73 (1.16)	-0.27 (1.06)	-0.51 (1.12)	-1.03 (1.11)	-0.55 (1.14)	-0.60 (0.96)
30. Working hours	-0.05 (1.08)	0.34 (1.25)	-0.44 (1.11)	0.17 (1.05)	0.11 (0.95)	-0.14 (0.91)	-0.13 (0.94)	0.07 (1.19)	-0.02 (1.03)	-0.35 (1.09)	0.03 (1.24)	0.01 (1.12)	-0.15 (0.92)
31. Income	-0.40 (1.03)	-0.34 (0.96)	-0.47 (0.98)	-0.29 (0.76)	-0.08 (0.90)	-0.39 (0.86)	-0.38 (0.85)	-0.35 (0.96)	-0.33 (0.97)	-0.38 (1.04)	-0.44 (0.95)	-0.27 (0.95)	-0.37 (0.89)
32. Economic burden	0.35 (1.18)	0.41 (0.98)	0.16 (1.19)	0.00 (0.80)	0.26 (0.92)	0.19 (0.94)	0.15 (0.92)	0.36 (1.04)	0.25 (1.07)	0.21 (1.26)	0.28 (1.06)	0.26 (1.03)	0.20 (0.98)

AFR – African Region, AMR – Region of Americas, EMR – Eastern Mediterranean Region, EUR – European Region, SEAR – South-East Asian Region, WPR – Western Pacific Region

**Figure 2:**
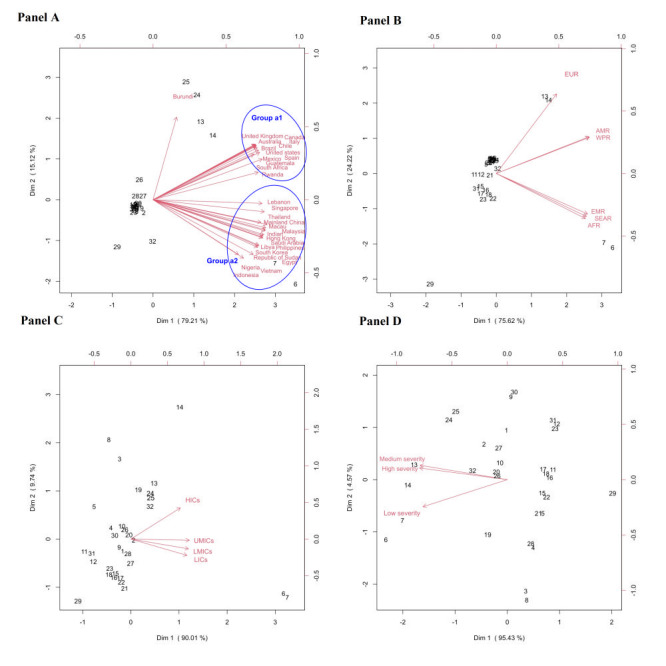
Biplots of multidimensional preference analysis visualizing countries’ preferences also by: **Panel A.** Country level impact. **Panel B.** World Health Organization (WHO) region level impact. **Panel C.** Economic development level impact. **Panel D.** COVID-19 severity level impact. The numbers in **Panels A**, **B**, **C** and **D** refer to those variables listed in **Table 2** that share the same corresponding number. Arrows in **Panels A**, **B**, **C** and **D** correspond to a country, a WHO region, an economic development level, and a COVID-19 severity level, respectively, and they point toward increased change. For each area, the projected length on the arrow corresponding to a particular country reflects the magnitude of the impact on that area relative to others in the country. AFR – African Region, AMR – Region of Americas, EMR – Eastern Mediterranean Region, EUR – European Region, SEAR – South-East Asian Region, WPR – Western Pacific Region, HICs – high income countries, UMICs – upper middle income countries, LMICs – lower middle income countries, LICs – low income countries

**Figure 2**, shows the biplots of the multidimensional preference analysis at the country, WHO region, economic development, and COVID-19 severity levels. Cooking at home (#7: indicated number 7 in the figures), having a meal at home (#6), duration of sitting (#13), duration of screen time (#14), and social activities (#29) were the areas most affected by COVID-19 across all countries and WHO regions (**Figure 2**, panel A and panel B) When comparing countries, Burundi showed a relatively higher increase in emotional distress (#25) and mental burden (#24) (**Figure 2**, panel A). The other countries formed two groups based on the extent of the impact of COVID-19 (**Figure 2**, panel A) Most of the countries in Group a1 were from AMR and EUR, whereas Group a2 comprised countries mostly from the EMR, SEAR, and WPR. Countries in Group a1 and the EUR, AMR, and WPR WHO regions experienced relatively more increased duration of sitting (#13) and screen time (#14), and reduced social activities (#29) (**Figure 2**, panel A and panel B). In contrast, Group a2 countries and the EMR, AFR, and SEAR WHO regions experienced a greater impact of having a meal (#6) and cooking at home (#7). Countries with low to upper-middle income levels experienced a greater increase in cooking (#7) and having meals at home (#6) (**Figure 2**, panel C), whereas high-income countries experienced a greater increase in the duration of screen time (#14), duration of sitting (#13), weight (#19), mental burden (#24), emotional distress (#25), and economic burden (#32) along with a greater reduction in social activities (#29). In countries with low COVID-19 severity levels, there was a greater increase in cooking (#7) and having meals at home (#6), whereas countries with medium to high levels of COVID-19 severity experienced a greater increase in the duration of sitting (#13) and screen time (#14), mental burden (#24), and emotional distress (#25), as well as a greater reduction in social activities (#29) (**Figure 2**, panel D).

### Preference for possible COVID-19 preparations

**Figure 3:**
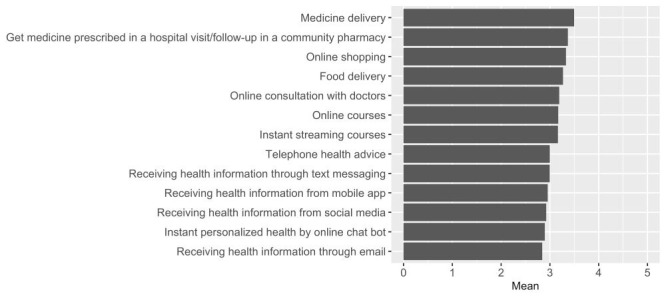
Overall weighted mean of the perceived importance for possible preparations. The ratings of 1, 2, 3, 4, and 5 indicate not important, somewhat important, important, very important, and extremely important, respectively.

**Table 3** summarises the weighted perceived importance of preparations by WHO region, economic development, and COVID-19 severity level. The corresponding by-country summary is provided in Table S4 in the **Online Supplementary Document** and visualised in Figure S1 in the **Online Supplementary Document****.** **Figure 3** shows that, on average, all listed possible preparations were perceived as at least important. Medicine delivery (m = 3.50, SD = 1.11) was rated as the most important, followed by getting prescribed medicine in a hospital visit / follow-up in a community pharmacy (m = 3.37, SD = 1.07), online shopping (m = 3.33, SD = 1.16), and food delivery (m = 3.27, SD = 1.18).

**Table 3:** Weighted mean (standard deviation) of perceived importance of possible preparations by World Health Organization (WHO) region, economic development level, and COVID-19 severity level.

Possible Preparations	World Health Organization regions						Economic development levels				COVID-19 severity levels		
	**AFR**	**AMR**	**EMR**	**EUR**	**SEAR**	**WPR**	**High**	**Upper-Middle**	**Lower-Middle**	**Low**	**High**	**Medium**	**Low**
**Perceived importance (1 = not important to 5 = extremely important)**													
1. Online consultation with doctors	3.23 (1.09)	3.33 (1.18)	3.17 (1.11)	3.31 (1.09)	2.85 (1.07)	3.21 (0.99)	3.20 (1.05)	3.25 (1.09)	3.05 (1.07)	3.23 (1.10)	3.24 (1.15)	3.17 (1.11)	3.18 (1.01)
2. Instant personalized health by online chatbots	3.08 (1.17)	2.96 (1.22)	2.89 (1.14)	2.76 (1.27)	2.67 (1.05)	2.90 (1.03)	2.77 (1.09)	3.07 (1.11)	2.82 (1.05)	3.13 (1.20)	2.85 (1.20)	2.93 (1.13)	2.90 (1.05)
3. Telephone health advice	3.34 (1.09)	3.00 (1.18)	3.03 (1.14)	3.18 (1.17)	2.75 (1.05)	2.97 (1.02)	2.93 (1.08)	3.03 (1.10)	2.99 (1.05)	3.30 (1.15)	2.98 (1.15)	3.02 (1.12)	2.99 (1.05)
4. Online courses	3.56 (1.04)	3.48 (1.15)	3.11 (1.20)	2.92 (1.18)	2.92 (1.14)	3.09 (1.06)	3.05 (1.12)	3.37 (1.11)	3.04 (1.09)	3.42 (1.13)	3.22 (1.23)	3.16 (1.14)	3.16 (1.07)
5. Instant streaming courses	3.40 (1.13)	3.48 (1.15)	3.03 (1.18)	2.97 (1.16)	2.99 (1.17)	3.11 (1.06)	3.06 (1.13)	3.37 (1.11)	3.05 (1.08)	3.22 (1.18)	3.22 (1.22)	3.15 (1.13)	3.15 (1.08)
6. Receiving health information through e-mail	3.40 (1.05)	2.96 (1.16)	2.75 (1.19)	2.88 (1.15)	2.72 (1.08)	2.77 (1.05)	2.65 (1.10)	3.02 (1.09)	2.95 (1.08)	3.12 (1.20)	2.90 (1.18)	2.87 (1.13)	2.81 (1.08)
7. Receiving health information through text messaging	3.44 (1.14)	2.86 (1.22)	2.92 (1.19)	2.73 (1.17)	2.90 (1.09)	3.03 (1.04)	2.89 (1.10)	3.08 (1.13)	3.05 (1.08)	3.23 (1.24)	2.81 (1.21)	3.07 (1.14)	3.04 (1.06)
8. Receiving health information from social media	3.29 (1.20)	2.80 (1.24)	3.07 (1.23)	2.21 (1.23)	2.89 (1.13)	2.90 (1.07)	2.71 (1.14)	3.12 (1.16)	3.02 (1.09)	3.28 (1.25)	2.72 (1.28)	3.06 (1.18)	2.94 (1.09)
9. Receiving health information from mobile apps	3.34 (1.12)	2.87 (1.19)	2.57 (1.15)	2.44 (1.24)	2.94 (1.07)	2.95 (1.05)	2.70 (1.14)	3.15 (1.08)	3.05 (1.03)	3.38 (1.11)	2.74 (1.26)	2.94 (1.07)	3.05 (1.07)
10. Getting medicine prescribed in a hospital visit / follow-up in a community pharmacy	3.35 (1.10)	3.53 (1.14)	3.46 (1.12)	3.46 (1.16)	3.04 (1.04)	3.34 (1.00)	3.37 (1.07)	3.45 (1.05)	3.22 (1.04)	3.34 (1.19)	3.46 (1.14)	3.38 (1.08)	3.32 (1.03)
11. Medicine delivery	3.52 (1.04)	3.71 (1.17)	3.57 (1.19)	3.35 (1.32)	3.29 (1.02)	3.44 (1.06)	3.48 (1.14)	3.63 (1.08)	3.34 (1.03)	3.37 (1.17)	3.56 (1.22)	3.53 (1.09)	3.45 (1.07)
12. Online shopping	3.44 (1.06)	3.45 (1.20)	3.20 (1.29)	3.43 (1.19)	3.16 (1.14)	3.35 (1.10)	3.43 (1.13)	3.39 (1.18)	2.98 (1.12)	3.28 (1.22)	3.29 (1.24)	3.25 (1.21)	3.39 (1.10)
13. Food delivery	3.32 (1.12)	3.36 (1.24)	3.12 (1.32)	3.39 (1.28)	3.17 (1.19)	3.29 (1.08)	3.29 (1.17)	3.39 (1.18)	3.05 (1.14)	3.13 (1.23)	3.20 (1.30)	3.24 (1.22)	3.31 (1.09)

AFR – African Region, AMR – Region of Americas, EMR – Eastern Mediterranean Region, EUR – European Region, SEAR – South-East Asian Region, WPR – Western Pacific Region

**Figure 4:**
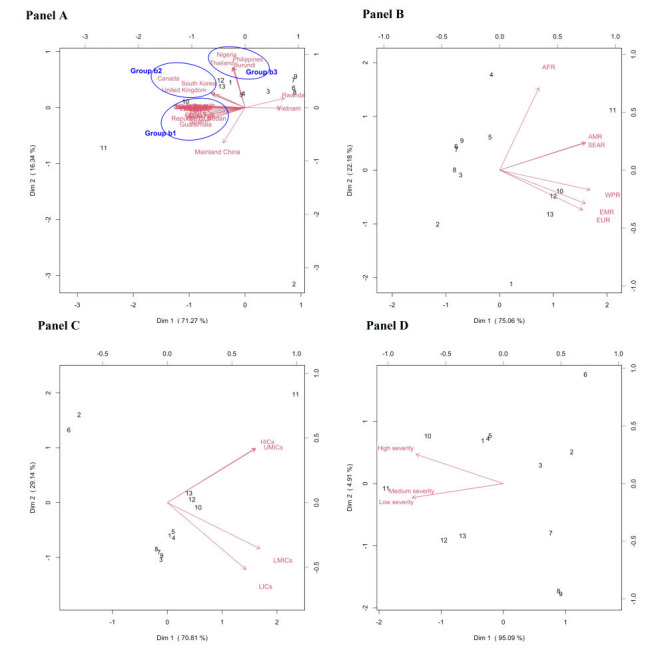
Biplots of multidimensional preference analysis visualizing countries’ preferences also by: **Panel A.** Country level impact. **Panel B.** World Health Organization (WHO) region level. **Panel C.** Economic developmental level. **Panel D.** COVID-19 severity level. The numbers in **panels A**, **B**, **C** and **D** refer to those variables listed in **Table 3** that share the same corresponding number. Arrows in **panels A**, **B**, **C** and **D** correspond to a country, a WHO region, an economic development level, and a COVID-19 severity level, respectively, and they point toward increased preference. For each area, the projected length on the arrow corresponding to a particular country reflects the magnitude of the impact on that area relative to others in the country. AFR – African Region, AMR – Region of Americas, EMR – Eastern Mediterranean Region, EUR – European Region, SEAR – South-East Asian Region, WPR – Western Pacific Region, HICs – high income countries, UMICs – upper middle income countries, LMICs – lower middle income countries, LICs – low income countries

**Figure 4** shows the biplots comparing countries, WHO regions, economic development levels, and COVID-19 severity levels. Medicine delivery (#11) was considered a crucial preparation in all WHO regions and countries except for Rwanda, Vietnam, Nigeria, the Philippines, Burundi, and Thailand (**Figure 4**, panel A and Panel B). Both Rwanda and Vietnam showed the highest preference for receiving health information through e-mail (#6), text messages (#7), social media (#8), mobile apps (#9), and instant personalised health advice via online chatbot (#2) (**Figure 4**, panel A). In Nigeria, Burundi, the Philippines, and Thailand, as well as in the WPR, EMR, and EUR WHO regions, online shopping (#12), medicine delivery (#11), getting medicine prescribed in a hospital visit / follow-up in a community pharmacy (#10), food delivery (#13), and online consultations with doctors (#1) were considered highly important (**Figure 4**, panel A and panel B). Instant personalised health delivered via online chatbots (#2) appeared to be rated as a lower priority in all countries except for mainland China, Rwanda, and Vietnam. When comparing countries by economic development level (**Figure 4**, panel C), those at the upper-middle to high income levels rated medicine delivery (#11) higher, whereas those at the low to lower-middle income levels considered other preparations similarly important, except for the lower rating of instant personalised health advice via online chatbot (#2) and receiving health information through e-mail (#6) (**Figure 4**, panel C). In countries with a high COVID-19 severity level, in addition to medicine delivery (#11), a higher preference was also reported for getting medicine prescribed during a hospital visit / follow-up in a community pharmacy (#10). In contrast, countries with a low-to-medium COVID-19 severity also rated online shopping (#12) and food delivery (#13) as high priorities (**Figure 4**, panel D).

## DISCUSSION

Our study findings confirm that COVID-19 has had a varying impact on individuals' lifestyles and health outcomes, and that there are differences in priority rankings for multiple pandemic preparations. Furthermore, we observed that the impact of COVID-19 and preparation preferences varied across countries, WHO regions, economic development levels, and COVID-19 severity levels. Specifically, the global community reported that the most significant changes were cooking and having more meals at home, having longer sitting and screen time, and engaging in fewer social activities compared to pre-pandemic times. The EUR, AMR, and WPR WHO regions and countries with a high-income level or medium to high COVID-19 severity were more adversely impacted in terms of sitting and screen time duration and social activities, whereas the AFR, SEAR, and EMR WHO regions and countries with low to upper-middle income levels or low COVID-19 severity experienced more cooking and eating at home. Countries with a high-income level or medium to high COVID-19 severity reported a higher perceived mental burden and emotional distress. Additionally, high-income countries perceived a greater negative impact on weight gain and economic burden. Nevertheless, there was an encouraging global reduction in the consumption of tobacco and alcohol. Respondents rated medicine delivery, getting medicine prescribed during a hospital visit / follow-up in a community pharmacy, and online shopping as the highest priorities in terms of preparations to better cope with the pandemic situation. In particular, medicine delivery was rated the highest among the preparation activities in most countries. In addition, a unique finding was that all low-income to lower-middle income countries considered all preparations important, except for obtaining personalised advice from online chatbots and receiving health information by email.

### COVID-19’s perceived impact

Increased cooking and having meals at home were the most significant lifestyle and health-related changes observed among 32 outcomes during the pandemic. However, Burundi did not experience a significant increase in cooking at home. This may be due to poverty and agriculture-based livelihoods [14], which made Burundians very reliant on homemade food even before the pandemic, resulting in limited room to increase or change cooking habits. Among the WHO regions, EMR, SEAR, and AFR showed the greatest increase in cooking and having meals at home. The restaurant-to-consumer delivery penetration in EMR, SEAR, and AFR ranges from 4.1% to 8.8%, which is substantially lower than the 15.0% to 23.8% in the other WHO regions [15], indicating a lower utility of food delivery services in the EMR, SEAR, and AFR. Thus, most people in these regions are more likely to cook at home than order delivery food service during the pandemic. Indeed, most countries in these regions had lower incomes, often with a lower COVID-19 severity [16,17], which renders cooking at home the most economical option. Another contributing factor is the generally larger household size with more children and older adults living together in the EMR, SEAR and AFR [18], as well as the tendency to cook more at home to protect vulnerable family members from infection.

Reduced social activities were the largest adverse impact of COVID-19, followed by increased sitting and screen time duration. Reduced social activities and increased sitting and screen time duration are more noticeable in the EUR, AMR, and WPR. These three WHO regions are more urbanized than the other regions [19]. Urbanisation renders the population vulnerable to sedentary behaviour. It reduces social activities under restriction measures because urban areas’ social and physical activities are more dependent on transportation, blue-green and open spaces, and city facilities [20]. Thus, high income countries with an 81% urbanisation rate in 2021, which significantly exceeds the 68% rate in upper-middle income countries [19], showed greater reductions in social activities and an increase in sitting and screen time duration. In particular, the EUR was the second most urbanised and had the greatest reduction in social activities and the largest increase in sitting and screen time duration. Indeed, the EUR also had the largest number of confirmed cases and may have experienced the most stringent outdoor restriction measures [17]. Generally, countries with medium to high COVID-19 severity reported more reduced social activities and increased sitting and screen time duration due to the more restrictive public health measures [17].

Interestingly, there was an overall reduction in tobacco and alcohol consumption and be fairly consistent across countries, regions, economic development levels, and COVID-19 severity levels. However, a previous systematic review of 37 studies covering 15 countries showed that more studies (19 studies, 51.4%) reported increased alcohol consumption during the pandemic [4], of which is contrary to our findings. This may be due to the lack of quantitative synthesis and not all included studies considered an exclusively community-based sample, whereas our surveys in all countries were conducted under the same protocol targeting community-based samples. Nevertheless, continual efforts to reduce smoking and alcohol addiction are desirable to reduce the risk of non-communicable diseases, including cancer, cardiovascular disease, chronic respiratory disease, heart disease, and liver cirrhosis.

In addition, high-income countries have also suffered from increased weight gain, mental and economic burdens, and emotional distress during the pandemic. A previous review claimed that low-to-middle income countries have insufficient resources to address mental health issues, so they deserve more attention than high-income countries [21]. However, high-income countries are indisputably the most affected and need considerable attention. Moreover, medium to high-severity countries also experienced increased mental burden and emotional distress, possibly due to more confirmed cases and stricter measures.

### Perceived importance of possible preparations

All 13 listed preparations were rated as at least important, with medicine delivery being the most preferred. Therefore, development or enhancement of medicine delivery services could, for the most part, meet residents’ demands. Most countries rated the importance of medicine delivery the highest, except Rwanda, Vietnam, Nigeria, the Philippines, Burundi, and Thailand. These countries have younger populations who are less likely living with a chronic condition and need regular medications. Besides, they are also developing nations, and medicine delivery may not be a popular service yet. Instead, they considered receiving health information through various electronic means important, which might result from their low internet penetration and limited access to health information [22]. This is consistent with the observation that upper-middle to high-income countries rated medicine delivery as the most important but not low to lower-middle countries.

Getting prescribed medicine during a hospital visit / follow-up in a community pharmacy was rated the second most important factor. Medications prescribed during a hospital visit are typically obtained from hospital's pharmacy department. However, the waiting time can be long, causing anxiety about the increased infection risk [23]. Obtaining prescription medicine in a community pharmacy closer to one’s residence is also desirable. However, access to prescribed medicine in a community pharmacy was not rated high in Rwanda and Vietnam as well as in the AFR, potentially owing to their younger populations and lower availability and poor quality of medications in the public sector in these countries or regions [24]. Providing adequate quality medications in communities is a priority area for development. This can be coupled with online consultations with doctors, which were also highly rated. Telemedicine has rapidly evolved and has successfully responded to the pandemic in consultation, follow-up, psychotherapeutic care, and getting the patient's family involved. Guidelines for practicing telemedicine have been developed to ensure the proper delivery of clinical care without compromising patient safety [25]. Moreover, there should be more development in information technology infrastructure, stricter data protection and privacy regulations, and more advanced technology for better body examination, such as test-specific medical devices equipped with smartphones, wearable devices, and remote palpation techniques.

Online shopping and food delivery were ranked third and fourth, respectively, and have undoubtedly become an important part of our lives because of their great flexibility and accessibility. Moreover, their use has increased since the COVID-19 pandemic, and was predicted to continue in the near future [26]. To enhance urban mobility and meet residents' demands, particularly in developing countries, strategists should focus more on e-commerce construction and development.

Instant personalised health delivered via online chatbots was rated relatively low on the list of important preparations; only mainland China rated it substantially important. The security and accuracy concerns with health chatbots might discourage most countries from using them [27]. Due to cultural differences, Chinese people treat robots more like real people and have greater trust and acceptance in them than people in the West [28]. Indeed, mental health adviser chatbots in mainland China received satisfactory feedback during the pandemic [29]. Nevertheless, artificial intelligence (AI) technology can be used for developing more sophisticated AI-powered chatbots to provide health advice and reduce viral transmission. Big data are particularly crucial for optimising algorithms by enhancing the accuracy and trustworthiness of data analytics.

### Limitations

Several limitations of this study are worth noting. First, the cross-sectional survey period lasted approximately one year, which may have introduced influences from variations in COVID-19 severity that could impact the results. To address this potential issue, we calculated the COVID-19 severity level for each country based on their exact survey period and conducted cross-severity group comparisons to gain a more accurate understanding of the impact of COVID-19 severity variation on the effects of COVID-19 and preparation preferences. Second, the use of convenient sampling methods may have introduced selection bias, as health care workers were overrepresented in the study and data were collected primarily through an online platform. As a result, there is a possibility of underrepresentation of individuals with low socio-economic status, limited digital literacy, or insufficient access to digital devices or the Internet. To compensate for this, we weighted our samples according to the corresponding populations to improve the representativeness of our sample. Third, the absence of *P*-values or confidence intervals from multidimensional preference analysis necessitates cautious interpretation of small differences observed in the biplots. Finally, the study's cross-sectional design might limit the exploration of longitudinal changes within each community.

## CONCLUSIONS

The COVID-19 pandemic has had unprecedented effects on our lives and health worldwide. Increased sitting and screen time duration and reduced social activity were more pronounced than in other areas, such as mental health. However, an increase in cooking and having meals at home and reductions in alcohol and tobacco consumption brought about by the pandemic will hopefully persist, thus contributing positively to healthy lifestyles. High-income countries, those with COVID-19 infection at medium-to-severe levels, and the EUR, AMR, and WPR WHO regions suffered more adverse effects from the pandemic. A health care system and technological infrastructure that facilitate medicine delivery, medicine prescription, and online shopping are priorities for coping with future pandemics. Our list of aspects impacted by COVID-19 and a priority list of preparations by perceived importance may offer essential information to policymakers, researchers, and other stakeholders to develop strategies to promote good health and overall quality of life, and better prepare for future pandemics.

## Additional material


Online Supplementary Document
