# Supplementary material for: Global impacts of COVID-19 on lifestyles and health and preparation preferences: An international survey of 30 countries
Source: J Glob Health. 2023 Aug 11;13:06031. doi: 10.7189/jogh.13.06031 (PMC10416140; doi:10.7189/jogh.13.06031)

ONLINE SUPPLEMENTARY DOCUMENT

Title: Global Impacts of COVID-19 on Lifestyles and Health and Preparation Preferences: An International Survey of 30 Countries

Authors: Jiaying Li, Daniel Yee Tak Fong, Kris Yuet Wan Lok, Janet Yuen Ha Wong, Mandy Man Ho, Edmond Pui Hang Choi, Vinciya Pandian, Patricia M Davidson, Wenjie Duan, Marie Tarrant, Jung Jae Lee, Chia-Chin Lin, Oluwadamilare Akingbade, Khalid M Alabdulwahhab, Mohammad Shakil Ahmad, Mohamed Alboraie, Meshari A Alzahrani, Anil S Bilimale, Sawitree Boonpatcharanon, Samuel Byiringiro, Muhammad Kamil Che Hasan, Luisa Clausi Schettini, Walter Corzo, Josephine M. De Leon, Anjanette S. De Leon, Hiba Deek, Fabio Efficace, Mayssah A El Nayal, Fathiya El-Raey, Eduardo Ensaldo-Carrasco, Pilar Escotorin, Oluwadamilola Agnes Fadodun, Israel Opeyemi Fawole, Yong-Shian Shawn Goh, Devi Irawan, Naimah Ebrahim Khan, Binu Koirala, Ashish Krishna, Cannas Kwok, Tung Thanh Le, Daniela Giambruno Leal, Miguel Ángel Lezana-Fernández, Emery Manirambona, Leandro Cruz Mantoani, Fernando Meneses-González, Iman Elmahdi Mohamed, Madeleine Mukeshimana, Chinh Thi Minh Nguyen, Huong Thi Thanh Nguyen, Khanh Thi Nguyen, Son Truong Nguyen, Mohd Said Nurumal, Aimable Nzabonimana, Nagla Abdelrahim Mohamed Ahmed Omer, Oluwabunmi Ogungbe, Angela Chiu Yin Poon, Areli Reséndiz-Rodriguez, Busayasachee Puang-Ngern, Ceryl G Sagun, Riyaz Ahmed Shaik, Nikhil Gauri Shankar, Kathrin Sommer, Edgardo Toro, Hanh Thi Hong Tran, Elvira L Urgel, Emmanuel Uwiringiyimana, Tita Vanichbuncha, Naglaa Youssef

Supplementary material summary

**Contents**	**Numbered pages**
Table S1. World Health Organization (WHO) regions, economic development levels and COVID-19 severity levels of the 30 countries or regions	2
Table S2. Demographic summary by country	3-10
Table S3. Weighted demographic summary by country	11-20
Table S4. Description of respondents' weighted perception of COVID-19's impact and preference for future preparations by country	21-29
Figure S1. Visualization of the perceived impact of COVID-19 on lifestyles and health-related areas as well as perceived importance of possible preparations by country, World Health Organization (WHO) region, economic development level, and COVID-19 severity level	27-49

Table S1. World Health Organization (WHO) regions, economic development levels and COVID-19 severity levels of the 30 countries or regions.

**Country**	**Region**	**Economic development level**	**COVID-19 severity level**
Australia	Western Pacific Region	High-income	Low severity
Brazil	Region of Americas	Upper-middle-income	Medium severity
Burundi	African Region	Low-income	Low severity
Canada	Region of Americas	High-income	High severity
Chile	Region of Americas	High-income	High severity
Egypt	Eastern Mediterranean Region	Lower-middle-income	Low severity
Guatemala	Region of Americas	Upper-middle-income	Medium severity
Hong Kong	Western Pacific Region	High-income	Low severity
India	South-East Asian Region	Lower-middle-income	Medium severity
Indonesia	South-East Asian Region	Lower-middle-income	Medium severity
Italy	European Region	High-income	High severity
Lebanon	Eastern Mediterranean Region	Upper-middle-income	High severity
Libya	Eastern Mediterranean Region	Upper-middle-income	High severity
Macau	Western Pacific Region	High-income	Low severity
Mainland China	Western Pacific Region	Upper-middle-income	Low severity
Malaysia	Western Pacific Region	Upper-middle-income	Medium severity
Mexico	Region of Americas	Upper-middle-income	High severity
Nigeria	African Region	Lower-middle-income	Low severity
Philippines	Western Pacific Region	Lower-middle-income	Medium severity
Republic of Sudan	Eastern Mediterranean Region	Low-income	Medium severity
Rwanda	African Region	Low-income	Medium severity
Saudi Arabia	Eastern Mediterranean Region	High-income	Medium severity
Singapore	Western Pacific Region	High-income	Medium severity
South African Regionica	African Region	Upper-middle-income	High severity
South Korea	Western Pacific Region	High-income	Low severity
Spain	European Region	High-income	High severity
Thailand	South-East Asian Region	Upper-middle-income	Low severity
United Kingdom	European Region	High-income	High severity
United States	Region of Americas	High-income	High severity
Vietnam	Western Pacific Region	Lower-middle-income	Low severity

Table S2. Demographic summary by country.

Characteristic	Australia, N = 639[1]	Brazil, N = 553[1]	Burundi, N = 369[1]	Canada, N = 368[1]	Chile, N = 342[1]	Egypt, N = 461[1]	Guatemala, N = 229[1]	Hong Kong, N = 2,127[1]	India, N = 529[1]	Indonesia, N = 482[1]	Italy, N = 203[1]	Lebanon, N = 440[1]	Libya, N = 645[1]	Macau, N = 250[1]	Mainland China, N = 667[1]
Age															
18-24	106 (17%)	128 (23%)	41 (11%)	20 (5·4%)	75 (22%)	103 (22%)	88 (38%)	196 (9·2%)	151 (29%)	433 (90%)	4 (2·0%)	210 (48%)	246 (38%)	70 (28%)	477 (72%)
25-29	84 (13%)	95 (17%)	44 (12%)	35 (9·5%)	45 (13%)	71 (15%)	43 (19%)	239 (11%)	98 (19%)	23 (4·8%)	12 (5·9%)	44 (10%)	138 (21%)	31 (12%)	94 (14%)
30-34	32 (5·0%)	73 (13%)	70 (19%)	48 (13%)	32 (9·4%)	85 (18%)	27 (12%)	315 (15%)	53 (10%)	11 (2·3%)	14 (6·9%)	53 (12%)	92 (14%)	43 (17%)	29 (4·3%)
35-39	49 (7·7%)	95 (17%)	77 (21%)	52 (14%)	40 (12%)	87 (19%)	22 (9·6%)	328 (15%)	94 (18%)	6 (1·2%)	16 (7·9%)	51 (12%)	70 (11%)	41 (16%)	15 (2·2%)
40-44	45 (7·0%)	41 (7·4%)	70 (19%)	33 (9·0%)	44 (13%)	52 (11%)	12 (5·2%)	240 (11%)	40 (7·6%)	2 (0·4%)	25 (12%)	29 (6·6%)	40 (6·2%)	27 (11%)	15 (2·2%)
45-49	80 (13%)	26 (4·7%)	32 (8·7%)	14 (3·8%)	43 (13%)	27 (5·9%)	18 (7·9%)	253 (12%)	31 (5·9%)	2 (0·4%)	29 (14%)	16 (3·6%)	22 (3·4%)	13 (5·2%)	25 (3·7%)
50-54	64 (10%)	28 (5·1%)	16 (4·3%)	26 (7·1%)	33 (9·6%)	22 (4·8%)	7 (3·1%)	165 (7·8%)	11 (2·1%)	3 (0·6%)	19 (9·4%)	16 (3·6%)	22 (3·4%)	11 (4·4%)	8 (1·2%)
55-59	69 (11%)	25 (4·5%)	9 (2·4%)	35 (9·5%)	12 (3·5%)	8 (1·7%)	6 (2·6%)	117 (5·5%)	17 (3·2%)	2 (0·4%)	36 (18%)	13 (3·0%)	10 (1·6%)	5 (2·0%)	4 (0·6%)
60-64	14 (2·2%)	25 (4·5%)	5 (1·4%)	49 (13%)	8 (2·3%)	4 (0·9%)	3 (1·3%)	125 (5·9%)	24 (4·5%)	0 (0%)	8 (3·9%)	6 (1·4%)	4 (0·6%)	8 (3·2%)	0 (0%)
>=65	96 (15%)	17 (3·1%)	5 (1·4%)	56 (15%)	10 (2·9%)	2 (0·4%)	3 (1·3%)	149 (7·0%)	10 (1·9%)	0 (0%)	40 (20%)	2 (0·5%)	1 (0·2%)	1 (0·4%)	0 (0%)
Gender															
Female	376 (59%)	395 (71%)	181 (49%)	297 (81%)	251 (73%)	266 (58%)	119 (52%)	1,186 (56%)	269 (51%)	396 (82%)	133 (66%)	291 (66%)	376 (58%)	201 (80%)	523 (78%)
Male	260 (41%)	157 (28%)	177 (48%)	64 (17%)	84 (25%)	195 (42%)	107 (47%)	940 (44%)	260 (49%)	86 (18%)	70 (34%)	149 (34%)	269 (42%)	49 (20%)	140 (21%)
Non-binary	3 (0·5%)	1 (0·2%)	11 (3·0%)	7 (1·9%)	7 (2·0%)	0 (0%)	3 (1·3%)	1 (<0·1%)	0 (0%)	0 (0%)	0 (0%)	0 (0%)	0 (0%)	0 (0%)	4 (0·6%)

Marital															
Married/Cohabitation/Common-law	332 (52%)	256 (46%)	236 (64%)	256 (70%)	120 (35%)	274 (59%)	71 (31%)	1,330 (63%)	275 (52%)	42 (8·7%)	145 (71%)	172 (39%)	211 (33%)	123 (49%)	113 (17%)
Separated/Divorced/Widowed	91 (14%)	39 (7·1%)	14 (3·8%)	40 (11%)	39 (11%)	17 (3·7%)	3 (1·3%)	99 (4·7%)	28 (5·3%)	0 (0%)	24 (12%)	12 (2·7%)	15 (2·3%)	5 (2·0%)	8 (1·2%)
Single	215 (34%)	258 (47%)	119 (32%)	72 (20%)	183 (54%)	170 (37%)	155 (68%)	698 (33%)	226 (43%)	440 (91%)	34 (17%)	256 (58%)	419 (65%)	122 (49%)	546 (82%)
Unknown	1 (0·2%)	0 (0%)	0 (0%)	0 (0%)	0 (0%)	0 (0%)	0 (0%)	0 (0%)	0 (0%)	0 (0%)	0 (0%)	0 (0%)	0 (0%)	0 (0%)	0 (0%)
Education															
Primary or below	0 (0%)	1 (0·2%)	139 (38%)	1 (0·3%)	3 (0·9%)	1 (0·2%)	0 (0%)	167 (7·9%)	42 (7·9%)	0 (0%)	3 (1·5%)	6 (1·4%)	3 (0·5%)	0 (0%)	2 (0·3%)
Secondary	156 (24%)	61 (11%)	180 (49%)	44 (12%)	68 (20%)	28 (6·1%)	49 (21%)	1,123 (53%)	61 (12%)	0 (0%)	6 (3·0%)	49 (11%)	96 (15%)	24 (9·6%)	59 (8·8%)
College	195 (31%)	52 (9·4%)	16 (4·3%)	103 (28%)	124 (36%)	33 (7·2%)	71 (31%)	181 (8·5%)	43 (8·1%)	91 (19%)	12 (5·9%)	27 (6·1%)	68 (11%)	4 (1·6%)	103 (15%)
Associate degree	130 (20%)	9 (1·6%)	6 (1·6%)	7 (1·9%)	8 (2·3%)	23 (5·0%)	2 (0·9%)	196 (9·2%)	40 (7·6%)	170 (35%)	63 (31%)	73 (17%)	92 (14%)	158 (63%)	8 (1·2%)
Bachelor	76 (12%)	154 (28%)	24 (6·5%)	126 (34%)	51 (15%)	230 (50%)	45 (20%)	415 (20%)	142 (27%)	208 (43%)	20 (9·9%)	199 (45%)	291 (45%)	51 (20%)	350 (52%)
Graduate	43 (6·7%)	276 (50%)	4 (1·1%)	87 (24%)	88 (26%)	146 (32%)	62 (27%)	45 (2·1%)	197 (37%)	13 (2·7%)	99 (49%)	86 (20%)	95 (15%)	13 (5·2%)	140 (21%)
Unkown	39 (6·1%)	0 (0%)	0 (0%)	0 (0%)	0 (0%)	0 (0%)	0 (0%)	0 (0%)	4 (0·8%)	0 (0%)	0 (0%)	0 (0%)	0 (0%)	0 (0%)	5 (0·7%)
Employment															
Job seeking	74 (12%)	10 (1·8%)	16 (4·3%)	19 (5·2%)	23 (6·7%)	24 (5·2%)	6 (2·6%)	69 (3·2%)	29 (5·5%)	5 (1·0%)	6 (3·0%)	60 (14%)	48 (7·4%)	3 (1·2%)	55 (8·2%)
Laid off	53 (8·3%)	1 (0·2%)	11 (3·0%)	10 (2·7%)	0 (0%)	0 (0%)	2 (0·9%)	45 (2·1%)	1 (0·2%)	0 (0%)	0 (0%)	5 (1·1%)	2 (0·3%)	0 (0%)	4 (0·6%)
Not in workforce	34 (5·3%)	8 (1·4%)	4 (1·1%)	50 (14%)	14 (4·1%)	46 (10·0%)	0 (0%)	214 (10%)	59 (11%)	2 (0·4%)	9 (4·4%)	34 (7·7%)	25 (3·9%)	4 (1·6%)	7 (1·0%)
Retired	41 (6·4%)	31 (5·6%)	3 (0·8%)	80 (22%)	12 (3·5%)	7 (1·5%)	1 (0·4%)	185 (8·7%)	12 (2·3%)	0 (0%)	45 (22%)	2 (0·5%)	2 (0·3%)	2 (0·8%)	2 (0·3%)

Self-employed	4 (0·6%)	62 (11%)	215 (58%)	28 (7·6%)	18 (5·3%)	50 (11%)	23 (10%)	34 (1·6%)	57 (11%)	11 (2·3%)	26 (13%)	39 (8·9%)	51 (7·9%)	13 (5·2%)	7 (1·0%)
Student	47 (7·4%)	130 (24%)	58 (16%)	26 (7·1%)	88 (26%)	89 (19%)	99 (43%)	162 (7·6%)	159 (30%)	417 (87%)	6 (3·0%)	149 (34%)	265 (41%)	57 (23%)	383 (57%)
Working (>=40hrs/wk)	58 (9·1%)	210 (38%)	21 (5·7%)	85 (23%)	153 (45%)	136 (30%)	65 (28%)	1,082 (51%)	153 (29%)	27 (5·6%)	55 (27%)	69 (16%)	75 (12%)	87 (35%)	128 (19%)
Working (1-39hrs/wk)	328 (51%)	101 (18%)	41 (11%)	70 (19%)	34 (9·9%)	109 (24%)	33 (14%)	336 (16%)	59 (11%)	20 (4·1%)	56 (28%)	82 (19%)	177 (27%)	84 (34%)	81 (12%)
Height (m)	1·69 (0·08) (1·50 to 1·96)	1·67 (0·10) (1·16 to 1·94)	1·63 (0·05) (1·52 to 1·90)	1·67 (0·10) (1·28 to 2·11)	1·64 (0·08) (1·40 to 1·90)	1·68 (0·10) (1·30 to 1·97)	1·66 (0·10) (1·44 to 1·98)	1·64 (0·08) (1·37 to 1·98)	1·64 (0·10) (1·43 to 1·96)	1·60 (0·06) (1·45 to 1·84)	1·69 (0·08) (1·50 to 1·94)	1·67 (0·09) (1·47 to 1·98)	1·67 (0·11) (1·20 to 2·00)	1·62 (0·08) (1·49 to 1·98)	1·64 (0·07) (1·30 to 1·86)
Unknown	12	0	1	5	0	0	2	1	4	0	0	3	2	0	2
Weight(kg)	72 (14) (27 to 130)	70 (16) (37 to 120)	60 (6) (45 to 89)	75 (18) (31 to 127)	71 (14) (41 to 113)	80 (18) (40 to 160)	68 (14) (39 to 110)	62 (10) (39 to 117)	66 (13) (34 to 104)	55 (11) (33 to 103)	70 (15) (45 to 122)	70 (16) (40 to 140)	71 (17) (36 to 150)	58 (13) (30 to 110)	61 (17) (35 to 135)
Unknown	9	0	0	8	0	0	2	0	4	0	0	0	0	0	2
BMI	25·2 (4·1) (11·7 to 45·8)	25·2 (4·7) (15·6 to 42·0)	22·5 (1·9) (15·5 to 34·0)	26·8 (5·6) (13·5 to 43·8)	26·3 (4·2) (17·6 to 44·4)	28·2 (5·6) (15·4 to 62·4)	24·4 (4·1) (13·9 to 37·1)	22·8 (3·0) (13·4 to 38·2)	24·4 (3·9) (14·5 to 36·1)	21·4 (3·9) (14·1 to 40·3)	24·7 (4·4) (17·3 to 38·9)	25·0 (4·7) (13·8 to 48·4)	25·6 (5·6) (12·9 to 52·1)	21·9 (4·0) (11·0 to 37·5)	22·4 (5·8) (12·1 to 48·4)
Unknown	15	0	1	9	0	0	4	1	5	0	0	3	2	0	2
BMI classification															
Severely underweight	7 (1·1%)	1 (0·2%)	2 (0·5%)	2 (0·6%)	0 (0%)	1 (0·2%)	4 (1·8%)	9 (0·4%)	6 (1·1%)	18 (3·7%)	0 (0%)	1 (0·2%)	11 (1·7%)	10 (4·0%)	15 (2·3%)
Underweight	17 (2·7%)	17 (3·1%)	2 (0·5%)	7 (1·9%)	4 (1·2%)	8 (1·7%)	4 (1·8%)	126 (5·9%)	21 (4·0%)	90 (19%)	4 (2·0%)	21 (4·8%)	22 (3·4%)	38 (15%)	100 (15%)
Normal weight	281 (45%)	280 (51%)	344 (93%)	142 (40%)	132 (39%)	68 (15%)	123 (55%)	925 (44%)	174 (33%)	240 (50%)	118 (58%)	148 (34%)	297 (46%)	120 (48%)	356 (54%)
Overweight	241 (39%)	171 (31%)	15 (4·1%)	114 (32%)	143 (42%)	59 (13%)	72 (32%)	620 (29%)	91 (17%)	58 (12%)	58 (29%)	62 (14%)	201 (31%)	32 (13%)	68 (10%)

Obesity	78 (12%)	84 (15%)	5 (1·4%)	94 (26%)	63 (18%)	325 (70%)	22 (9·8%)	446 (21%)	232 (44%)	76 (16%)	23 (11%)	205 (47%)	112 (17%)	50 (20%)	126 (19%)
Unknown	15	0	1	9	0	0	4	1	5	0	0	3	2	0	2
Pregnant															
Not applicable	261 (41%)	157 (28%)	177 (48%)	64 (17%)	84 (25%)	219 (48%)	107 (47%)	940 (44%)	260 (49%)	86 (18%)	71 (35%)	159 (36%)	269 (42%)	49 (20%)	141 (21%)
No	372 (58%)	393 (71%)	159 (43%)	280 (76%)	256 (75%)	242 (52%)	119 (52%)	1,177 (55%)	268 (51%)	390 (81%)	131 (65%)	277 (63%)	357 (55%)	189 (76%)	506 (76%)
Yes	6 (0·9%)	3 (0·5%)	33 (8·9%)	24 (6·5%)	2 (0·6%)	0 (0%)	3 (1·3%)	10 (0·5%)	1 (0·2%)	6 (1·2%)	1 (0·5%)	4 (0·9%)	19 (2·9%)	12 (4·8%)	20 (3·0%)
Gestational week	22 (13) (6 to 40)	6 (1) (6 to 7)	4 (1) (3 to 7)	24 (12) (4 to 39)	30 (NA) (30 to 30)	NA (NA) (Inf to -Inf)	NA (NA) (Inf to -Inf)	24 (9) (15 to 40)	NA (NA) (Inf to -Inf)	19 (12) (8 to 32)	12 (NA) (12 to 12)	16 (12) (6 to 30)	22 (12) (9 to 36)	30 (13) (5 to 40)	30 (12) (4 to 41)
Unknown	633	551	348	345	341	461	229	2,117	529	477	202	436	638	238	655
Regular medical follow-up before COVID-19															
NA	1 (0·2%)	0 (0%)	0 (0%)	0 (0%)	0 (0%)	0 (0%)	0 (0%)	0 (0%)	0 (0%)	0 (0%)	0 (0%)	0 (0%)	0 (0%)	0 (0%)	0 (0%)
No	475 (74%)	393 (71%)	352 (95%)	119 (32%)	207 (61%)	347 (75%)	170 (74%)	1,606 (76%)	377 (71%)	365 (76%)	44 (22%)	287 (65%)	450 (70%)	199 (80%)	628 (94%)
Yes	163 (26%)	160 (29%)	17 (4·6%)	249 (68%)	135 (39%)	114 (25%)	59 (26%)	521 (24%)	152 (29%)	117 (24%)	159 (78%)	153 (35%)	195 (30%)	51 (20%)	39 (5·8%)
Practising healthcare professional															
NA	0 (0%)	0 (0%)	0 (0%)	0 (0%)	0 (0%)	0 (0%)	0 (0%)	0 (0%)	0 (0%)	1 (0·2%)	0 (0%)	0 (0%)	0 (0%)	0 (0%)	0 (0%)
No	563 (88%)	339 (61%)	356 (96%)	332 (90%)	303 (89%)	253 (55%)	177 (77%)	2,068 (97%)	342 (65%)	261 (54%)	184 (91%)	261 (59%)	427 (66%)	148 (59%)	645 (97%)
Yes	76 (12%)	214 (39%)	13 (3·5%)	36 (9·8%)	39 (11%)	208 (45%)	52 (23%)	59 (2·8%)	187 (35%)	220 (46%)	19 (9·4%)	179 (41%)	218 (34%)	102 (41%)	22 (3·3%)
Number of children less than 18 years old															

No	513 (80%)	410 (74%)	153 (41%)	242 (66%)	220 (64%)	215 (47%)	182 (79%)	1,518 (71%)	328 (62%)	465 (96%)	145 (71%)	307 (70%)	474 (73%)	132 (53%)	383 (57%)
Yes	126 (20%)	143 (26%)	216 (59%)	126 (34%)	122 (36%)	246 (53%)	47 (21%)	609 (29%)	201 (38%)	17 (3·5%)	58 (29%)	133 (30%)	171 (27%)	118 (47%)	284 (43%)
Number of children aged above 18	0·34 (0·74) (0·00 to 3·00)	0·40 (0·82) (0·00 to 8·00)	1·25 (1·21) (0·00 to 6·00)	0·61 (1·01) (0·00 to 5·00)	0·55 (0·88) (0·00 to 5·00)	1·13 (1·27) (0·00 to 5·00)	0·32 (0·74) (0·00 to 5·00)	0·39 (0·68) (0·00 to 4·00)	0·59 (0·86) (0·00 to 5·00)	0·04 (0·20) (0·00 to 2·00)	0·47 (0·83) (0·00 to 3·00)	0·69 (1·17) (0·00 to 5·00)	0·71 (1·39) (0·00 to 6·00)	0·80 (1·05) (0·00 to 7·00)	0·54 (0·75) (0·00 to 6·00)
Unknown	0	7	1	3	3	9	3	0	1	0	0	0	11	1	7
Number of people lived with	2·90 (1·19) (1·00 to 6·00)	2·89 (1·29) (1·00 to 7·00)	4·08 (1·31) (1·00 to 8·00)	2·61 (1·37) (1·00 to 7·00)	3·26 (1·44) (1·00 to 8·00)	4·67 (1·83) (1·00 to 17·00)	4·52 (2·25) (1·00 to 12·00)	3·35 (1·08) (1·00 to 9·00)	3·86 (1·72) (1·00 to 20·00)	4·35 (1·30) (1·00 to 9·00)	2·50 (1·17) (1·00 to 6·00)	4·60 (1·54) (1·00 to 11·00)	6·19 (2·88) (1·00 to 26·00)	3·84 (1·45) (1·00 to 12·00)	3·75 (1·39) (1·00 to 10·00)
Unknown	51	0	2	2	0	4	0	0	2	0	2	0	21	0	12
Perceived social rank	3·13 (0·93) (1·00 to 5·00)	3·42 (0·79) (1·00 to 5·00)	1·66 (0·68) (1·00 to 4·00)	3·46 (0·93) (1·00 to 5·00)	3·12 (0·77) (1·00 to 5·00)	3·32 (0·91) (1·00 to 5·00)	3·15 (0·75) (1·00 to 5·00)	2·73 (0·76) (1·00 to 5·00)	3·18 (1·08) (1·00 to 5·00)	3·51 (0·87) (1·00 to 5·00)	3·60 (0·69) (2·00 to 5·00)	3·03 (0·81) (1·00 to 5·00)	3·21 (1·11) (1·00 to 5·00)	3·21 (0·70) (1·00 to 5·00)	2·77 (0·77) (1·00 to 5·00)
Unknown	1	0	0	0	0	0	0	0	1	1	0	0	0	0	1
House size	175 (138) (10 to 575)	131 (91) (10 to 500)	83 (26) (10 to 202)	146 (91) (3 to 550)	91 (56) (10 to 400)	119 (60) (10 to 400)	148 (126) (10 to 550)	38 (18) (9 to 195)	57 (67) (1 to 581)	243 (141) (10 to 590)	114 (48) (40 to 315)	145 (78) (10 to 570)	234 (138) (11 to 500)	153 (142) (10 to 570)	112 (49) (11 to 400)
Unknown	452	35	3	116	27	2	40	1	44	0	4	47	360	0	2

（Continued）

Characteristic	Malaysia, N = 535[1]	Mexico, N = 1,016[1]	Nigeria, N = 590[1]	Philippines, N = 457[1]	Republic Of Sudan, N = 538[1]	Rwanda, N = 150[1]	Saudi Arabia, N = 631[1]	Singapore, N = 237[1]	South Africa, N = 198[1]	South Korea, N = 2,238[1]	Spain, N = 51[1]	Thailand, N = 723[1]	United Kingdom, N = 212[1]	United States, N = 213[1]	Vietnam, N = 419[1]
Age															
18-24	315 (59%)	389 (38%)	171 (29%)	225 (49%)	375 (70%)	10 (6·7%)	220 (35%)	80 (34%)	101 (51%)	161 (7·2%)	12 (24%)	152 (21%)	83 (39%)	19 (8·9%)	196 (47%)
25-29	23 (4·3%)	140 (14%)	255 (43%)	46 (10%)	85 (16%)	53 (35%)	121 (19%)	13 (5·5%)	19 (9·6%)	290 (13%)	2 (3·9%)	109 (15%)	27 (13%)	32 (15%)	34 (8·1%)
30-34	45 (8·4%)	95 (9·4%)	81 (14%)	54 (12%)	21 (3·9%)	44 (29%)	84 (13%)	26 (11%)	17 (8·6%)	227 (10%)	1 (2·0%)	136 (19%)	30 (14%)	32 (15%)	61 (15%)
35-39	25 (4·7%)	99 (9·7%)	38 (6·4%)	29 (6·3%)	9 (1·7%)	24 (16%)	73 (12%)	34 (14%)	20 (10%)	222 (9·9%)	5 (9·8%)	104 (14%)	25 (12%)	28 (13%)	77 (18%)
40-44	26 (4·9%)	101 (9·9%)	18 (3·1%)	20 (4·4%)	13 (2·4%)	12 (8·0%)	45 (7·1%)	29 (12%)	10 (5·1%)	273 (12%)	10 (20%)	93 (13%)	15 (7·1%)	21 (9·9%)	26 (6·2%)
45-49	45 (8·4%)	62 (6·1%)	13 (2·2%)	18 (3·9%)	12 (2·2%)	3 (2·0%)	44 (7·0%)	25 (11%)	13 (6·6%)	178 (8·0%)	10 (20%)	49 (6·8%)	8 (3·8%)	30 (14%)	16 (3·8%)
50-54	34 (6·4%)	47 (4·6%)	10 (1·7%)	27 (5·9%)	13 (2·4%)	1 (0·7%)	24 (3·8%)	12 (5·1%)	3 (1·5%)	288 (13%)	4 (7·8%)	32 (4·4%)	7 (3·3%)	14 (6·6%)	8 (1·9%)
55-59	10 (1·9%)	36 (3·5%)	3 (0·5%)	17 (3·7%)	4 (0·7%)	2 (1·3%)	10 (1·6%)	7 (3·0%)	9 (4·5%)	156 (7·0%)	4 (7·8%)	22 (3·0%)	7 (3·3%)	11 (5·2%)	1 (0·2%)
60-64	7 (1·3%)	31 (3·1%)	1 (0·2%)	6 (1·3%)	4 (0·7%)	1 (0·7%)	8 (1·3%)	6 (2·5%)	6 (3·0%)	321 (14%)	0 (0%)	10 (1·4%)	4 (1·9%)	11 (5·2%)	0 (0%)
>=65	5 (0·9%)	16 (1·6%)	0 (0%)	15 (3·3%)	2 (0·4%)	0 (0%)	2 (0·3%)	5 (2·1%)	0 (0%)	122 (5·5%)	3 (5·9%)	16 (2·2%)	6 (2·8%)	15 (7·0%)	0 (0%)
Gender															
Female	367 (69%)	661 (65%)	331 (56%)	324 (71%)	345 (64%)	41 (27%)	372 (59%)	169 (71%)	136 (69%)	1,128 (50%)	34 (67%)	512 (71%)	155 (73%)	191 (90%)	325 (78%)
Male	166 (31%)	347 (34%)	255 (43%)	124 (27%)	193 (36%)	106 (71%)	259 (41%)	66 (28%)	61 (31%)	1,103 (49%)	16 (31%)	192 (27%)	56 (26%)	20 (9·4%)	90 (21%)
Non-binary	2 (0·4%)	8 (0·8%)	4 (0·7%)	9 (2·0%)	0 (0%)	3 (2·0%)	0 (0%)	2 (0·8%)	1 (0·5%)	7 (0·3%)	1 (2·0%)	19 (2·6%)	1 (0·5%)	2 (0·9%)	4 (1·0%)

Marital															
Married/Cohabitation/Common-law	179 (33%)	309 (30%)	156 (26%)	124 (27%)	75 (14%)	69 (46%)	283 (45%)	109 (46%)	67 (34%)	1,331 (59%)	31 (61%)	168 (23%)	96 (45%)	127 (60%)	195 (47%)
Separated/Divorced/Widowed	7 (1·3%)	59 (5·8%)	7 (1·2%)	16 (3·5%)	9 (1·7%)	5 (3·3%)	20 (3·2%)	6 (2·5%)	7 (3·5%)	112 (5·0%)	2 (3·9%)	18 (2·5%)	9 (4·2%)	14 (6·6%)	7 (1·7%)
Single	349 (65%)	648 (64%)	427 (72%)	317 (69%)	454 (84%)	76 (51%)	328 (52%)	122 (51%)	124 (63%)	795 (36%)	18 (35%)	537 (74%)	107 (50%)	72 (34%)	217 (52%)
Education															
Primary or below	3 (0·6%)	12 (1·2%)	3 (0·5%)	3 (0·7%)	3 (0·6%)	0 (0%)	5 (0·8%)	1 (0·4%)	0 (0%)	3 (0·1%)	0 (0%)	3 (0·4%)	0 (0%)	1 (0·5%)	0 (0%)
Secondary	35 (6·5%)	206 (20%)	28 (4·7%)	58 (13%)	58 (11%)	25 (17%)	105 (17%)	2 (0·8%)	35 (18%)	16 (0·7%)	9 (18%)	25 (3·5%)	15 (7·1%)	2 (0·9%)	4 (1·0%)
College	89 (17%)	162 (16%)	166 (28%)	115 (25%)	22 (4·1%)	9 (6·0%)	21 (3·3%)	47 (20%)	23 (12%)	405 (18%)	12 (24%)	6 (0·8%)	49 (23%)	2 (0·9%)	7 (1·7%)
Associate degree	115 (21%)	3 (0·3%)	22 (3·7%)	9 (2·0%)	26 (4·8%)	8 (5·3%)	20 (3·2%)	1 (0·4%)	2 (1·0%)	344 (15%)	3 (5·9%)	8 (1·1%)	3 (1·4%)	2 (0·9%)	25 (6·0%)
Bachelor	265 (50%)	358 (35%)	299 (51%)	191 (42%)	392 (73%)	68 (45%)	389 (62%)	132 (56%)	105 (53%)	1,222 (55%)	5 (9·8%)	296 (41%)	86 (41%)	14 (6·6%)	296 (71%)
Graduate	27 (5·0%)	275 (27%)	72 (12%)	81 (18%)	37 (6·9%)	39 (26%)	90 (14%)	54 (23%)	33 (17%)	248 (11%)	22 (43%)	382 (53%)	58 (27%)	75 (35%)	87 (21%)
Unkown	1 (0·2%)	0 (0%)	0 (0%)	0 (0%)	0 (0%)	1 (0·7%)	1 (0·2%)	0 (0%)	0 (0%)	0 (0%)	0 (0%)	3 (0·4%)	1 (0·5%)	117 (55%)	0 (0%)
Employment	3 (0·6%)	12 (1·2%)	3 (0·5%)	3 (0·7%)	3 (0·6%)	0 (0%)	5 (0·8%)	1 (0·4%)	0 (0%)	3 (0·1%)	0 (0%)	3 (0·4%)	0 (0%)	1 (0·5%)	0 (0%)
Job seeking	16 (3·0%)	33 (3·2%)	65 (11%)	19 (4·2%)	42 (7·8%)	17 (11%)	79 (13%)	1 (0·4%)	8 (4·0%)	116 (5·2%)	2 (3·9%)	24 (3·3%)	5 (2·4%)	3 (1·4%)	8 (1·9%)
Laid off	1 (0·2%)	7 (0·7%)	4 (0·7%)	0 (0%)	1 (0·2%)	2 (1·3%)	5 (0·8%)	0 (0%)	1 (0·5%)	11 (0·5%)	0 (0%)	2 (0·3%)	2 (0·9%)	0 (0%)	0 (0%)
Not in workforce	17 (3·2%)	27 (2·7%)	8 (1·4%)	18 (3·9%)	20 (3·7%)	1 (0·7%)	60 (9·5%)	2 (0·8%)	2 (1·0%)	308 (14%)	0 (0%)	8 (1·1%)	3 (1·4%)	6 (2·8%)	0 (0%)
Retired	14 (2·6%)	19 (1·9%)	0 (0%)	14 (3·1%)	1 (0·2%)	0 (0%)	21 (3·3%)	5 (2·1%)	4 (2·0%)	69 (3·1%)	2 (3·9%)	20 (2·8%)	7 (3·3%)	9 (4·2%)	4 (1·0%)

- Self-employed	19 (3·6%)	91 (9·0%)	28 (4·7%)	32 (7·0%)	31 (5·8%)	11 (7·3%)	8 (1·3%)	4 (1·7%)	19 (9·6%)	300 (13%)	9 (18%)	63 (8·7%)	9 (4·2%)	8 (3·8%)	39 (9·3%)
- Student	309 (58%)	351 (35%)	200 (34%)	203 (44%)	352 (65%)	15 (10%)	211 (33%)	92 (39%)	95 (48%)	148 (6·6%)	12 (24%)	159 (22%)	103 (49%)	41 (19%)	163 (39%)
- Working (>=40hrs/wk)	119 (22%)	299 (29%)	192 (33%)	132 (29%)	39 (7·2%)	78 (52%)	124 (20%)	117 (49%)	55 (28%)	988 (44%)	13 (25%)	325 (45%)	45 (21%)	106 (50%)	160 (38%)
- Working (1-39hrs/wk)	40 (7·5%)	189 (19%)	93 (16%)	39 (8·5%)	52 (9·7%)	26 (17%)	123 (19%)	16 (6·8%)	14 (7·1%)	298 (13%)	13 (25%)	122 (17%)	38 (18%)	40 (19%)	45 (11%)
Height (m)	1·60 (0·08) (1·40 to 1·85)	1·64 (0·09) (1·23 to 2·10)	1·66 (0·14) (1·20 to 2·20)	1·60 (0·10) (1·15 to 1·96)	1·66 (0·11) (1·12 to 2·00)	1·68 (0·09) (1·40 to 1·93)	1·65 (0·10) (1·35 to 1·95)	1·64 (0·08) (1·48 to 1·85)	1·62 (0·15) (1·10 to 1·96)	1·67 (0·08) (1·40 to 1·90)	1·68 (0·08) (1·50 to 1·85)	1·63 (0·08) (1·43 to 1·85)	1·67 (0·11) (1·25 to 1·95)	1·65 (0·07) (1·45 to 1·80)	1·59 (0·07) (1·45 to 1·84)
- Unknown	1	4	10	3	7	5	1	0	6	0	0	1	0	8	0
Weight(kg)	61 (13) (35 to 98)	69 (14) (40 to 120)	63 (11) (25 to 100)	62 (15) (21 to 112)	64 (14) (35 to 120)	68 (12) (32 to 97)	71 (19) (35 to 145)	64 (14) (39 to 103)	66 (18) (40 to 140)	65 (13) (32 to 137)	68 (13) (49 to 100)	62 (14) (37 to 108)	73 (18) (16 to 160)	73 (17) (40 to 134)	54 (9) (38 to 88)
- Unknown	0	2	3	0	0	0	1	0	2	0	0	2	1	8	0
BMI	24·0 (4·6) (14·3 to 39·4)	25·6 (4·3) (13·8 to 39·4)	23·2 (5·3) (11·2 to 51·7)	24·3 (5·5) (7·5 to 47·4)	23·2 (4·7) (10·8 to 38·3)	24·0 (3·7) (12·9 to 32·9)	26·1 (5·7) (12·9 to 53·5)	23·5 (4·3) (15·2 to 36·9)	25·8 (7·7) (14·3 to 56·7)	23·2 (3·4) (13·3 to 45·7)	24·2 (3·6) (18·0 to 36·3)	23·1 (4·3) (14·1 to 38·9)	26·3 (6·4) (6·2 to 50·6)	26·8 (6·2) (16·9 to 50·2)	21·2 (2·7) (14·7 to 32·9)
- Unknown	1	6	11	3	7	5	1	0	7	0	0	2	1	11	0
BMI classification															
- Severely underweight	14 (2·6%)	4 (0·4%)	30 (5·2%)	11 (2·4%)	26 (4·9%)	3 (2·1%)	7 (1·1%)	2 (0·8%)	4 (2·1%)	11 (0·5%)	0 (0%)	13 (1·8%)	3 (1·4%)	0 (0%)	7 (1·7%)
- Underweight	37 (6·9%)	28 (2·8%)	49 (8·5%)	35 (7·7%)	50 (9·4%)	1 (0·7%)	33 (5·2%)	21 (8·9%)	19 (9·9%)	108 (4·8%)	2 (3·9%)	60 (8·3%)	7 (3·3%)	2 (1·0%)	53 (13%)
- Normal weight	199 (37%)	440 (44%)	343 (59%)	175 (39%)	279 (53%)	90 (62%)	155 (25%)	99 (42%)	80 (42%)	1,032 (46%)	32 (63%)	338 (47%)	90 (43%)	92 (46%)	264 (63%)

Overweight	85 (16%)	378 (37%)	101 (17%)	63 (14%)	124 (23%)	42 (29%)	95 (15%)	35 (15%)	45 (24%)	485 (22%)	14 (27%)	116 (16%)	72 (34%)	61 (30%)	58 (14%)
Obesity	199 (37%)	160 (16%)	56 (9·7%)	170 (37%)	52 (9·8%)	9 (6·2%)	340 (54%)	80 (34%)	43 (23%)	602 (27%)	3 (5·9%)	194 (27%)	39 (18%)	47 (23%)	37 (8·8%)
Unknown	1	6	11	3	7	5	1	0	7	0	0	2	1	11	0
Pregnant															
Not applicable	166 (31%)	347 (34%)	255 (43%)	124 (27%)	193 (36%)	106 (71%)	259 (41%)	66 (28%)	61 (31%)	1,110 (50%)	16 (31%)	192 (27%)	56 (26%)	22 (10%)	90 (21%)
No	363 (68%)	665 (65%)	320 (54%)	330 (72%)	342 (64%)	40 (27%)	356 (56%)	170 (72%)	136 (69%)	1,119 (50%)	35 (69%)	527 (73%)	156 (74%)	187 (88%)	317 (76%)
Yes	6 (1·1%)	4 (0·4%)	15 (2·5%)	3 (0·7%)	3 (0·6%)	4 (2·7%)	16 (2·5%)	1 (0·4%)	1 (0·5%)	9 (0·4%)	0 (0%)	4 (0·6%)	0 (0%)	4 (1·9%)	12 (2·9%)
Gestational week	25 (12) (12 to 35)	24 (NA) (24 to 24)	27 (8) (17 to 34)	NA (NA) (Inf to -Inf)	7 (1) (6 to 8)	6 (3) (4 to 9)	15 (11) (5 to 30)	10 (NA) (10 to 10)	NA (NA) (Inf to -Inf)	9 (2) (5 to 10)	NA (NA) (Inf to -Inf)	18 (15) (6 to 36)	NA (NA) (Inf to -Inf)	29 (4) (25 to 34)	31 (8) (15 to 36)
Unknown	532	1,015	586	457	536	147	626	236	198	2,229	51	719	212	209	413
Regular medical follow-up before COVID-19															
NA	0 (0%)	0 (0%)	0 (0%)	0 (0%)	0 (0%)	0 (0%)	0 (0%)	0 (0%)	0 (0%)	0 (0%)	0 (0%)	0 (0%)	0 (0%)	2 (0·9%)	0 (0%)
No	436 (81%)	702 (69%)	469 (79%)	344 (75%)	450 (84%)	139 (93%)	448 (71%)	187 (79%)	151 (76%)	1,372 (61%)	35 (69%)	502 (69%)	154 (73%)	88 (41%)	62 (15%)
Yes	99 (19%)	314 (31%)	121 (21%)	113 (25%)	88 (16%)	11 (7·3%)	183 (29%)	50 (21%)	47 (24%)	866 (39%)	16 (31%)	221 (31%)	58 (27%)	123 (58%)	357 (85%)
Practising healthcare professional															
NA	0 (0%)	0 (0%)	0 (0%)	0 (0%)	0 (0%)	0 (0%)	0 (0%)	0 (0%)	0 (0%)	0 (0%)	0 (0%)	0 (0%)	0 (0%)	0 (0%)	0 (0%)
No	290 (54%)	425 (42%)	276 (47%)	267 (58%)	357 (66%)	71 (47%)	466 (74%)	124 (52%)	133 (67%)	2,150 (96%)	42 (82%)	634 (88%)	162 (76%)	81 (38%)	229 (55%)

Yes	245 (46%)	591 (58%)	314 (53%)	190 (42%)	181 (34%)	79 (53%)	165 (26%)	113 (48%)	65 (33%)	88 (3·9%)	9 (18%)	89 (12%)	50 (24%)	132 (62%)	190 (45%)
Number of children less than 18 years old															
No	398 (74%)	802 (79%)	468 (79%)	352 (77%)	484 (90%)	87 (58%)	390 (62%)	164 (69%)	163 (82%)	1,634 (73%)	33 (65%)	631 (87%)	163 (77%)	147 (69%)	242 (58%)
Yes	137 (26%)	214 (21%)	122 (21%)	105 (23%)	54 (10%)	63 (42%)	241 (38%)	73 (31%)	35 (18%)	604 (27%)	18 (35%)	92 (13%)	49 (23%)	66 (31%)	177 (42%)
Number of children aged above 18	0·58 (1·17) (0·00 to 8·00)	0·34 (0·84) (0·00 to 11·00)	0·48 (1·21) (0·00 to 10·00)	0·36 (0·79) (0·00 to 4·00)	0·26 (0·90) (0·00 to 6·00)	0·83 (1·20) (0·00 to 5·00)	0·99 (1·55) (0·00 to 7·00)	0·61 (1·02) (0·00 to 4·00)	0·30 (0·71) (0·00 to 3·00)	0·42 (0·76) (0·00 to 5·00)	0·63 (0·94) (0·00 to 3·00)	0·18 (0·52) (0·00 to 4·00)	0·41 (0·90) (0·00 to 6·00)	0·49 (0·83) (0·00 to 4·00)	0·76 (0·96) (0·00 to 4·00)
Unknown	0	11	3	2	3	0	17	0	0	0	0	2	1	1	0
Number of people lived with	5·07 (2·17) (1·00 to 16·00)	3·86 (1·83) (1·00 to 14·00)	4·26 (2·61) (1·00 to 23·00)	4·97 (2·25) (1·00 to 14·00)	6·59 (2·60) (1·00 to 18·00)	3·83 (2·21) (1·00 to 10·00)	6·25 (2·76) (1·00 to 21·00)	4·21 (1·47) (1·00 to 10·00)	4·33 (2·16) (1·00 to 12·00)	3·05 (1·15) (1·00 to 7·00)	3·35 (1·38) (1·00 to 6·00)	3·35 (1·91) (1·00 to 18·00)	3·14 (1·49) (1·00 to 8·00)	2·67 (1·31) (1·00 to 8·00)	4·31 (2·03) (1·00 to 12·00)
Unknown	2	2	4	5	35	0	32	0	1	0	0	1	0	10	6
Perceived social rank	3·49 (0·81) (1·00 to 5·00)	3·01 (0·64) (1·00 to 5·00)	2·99 (0·90) (1·00 to 5·00)	3·27 (0·70) (1·00 to 5·00)	3·17 (1·03) (1·00 to 5·00)	2·83 (0·75) (1·00 to 5·00)	3·46 (0·97) (1·00 to 5·00)	3·32 (0·71) (1·00 to 5·00)	3·19 (1·01) (1·00 to 5·00)	2·95 (0·70) (1·00 to 5·00)	3·00 (0·66) (1·00 to 5·00)	3·84 (0·87) (1·00 to 5·00)	3·34 (0·77) (1·00 to 5·00)	3·77 (0·76) (1·00 to 5·00)	3·23 (0·72) (1·00 to 5·00)
Unknown	0	0	0	0	0	0	0	0	0	0	0	0	0	2	0
House size	136 (127) (1 to 594)	125 (99) (10 to 500)	68 (80) (1 to 555)	120 (107) (1 to 561)	243 (145) (10 to 500)	98 (45) (12 to 249)	247 (155) (10 to 555)	115 (71) (1 to 500)	146 (124) (1 to 550)	31 (13) (10 to 100)	110 (62) (25 to	134 (119) (10 to 540)	98 (89) (1 to 500)	195 (109) (20 to 560)	168 (141) (10 to 650)

											300)				
Unknown	17	85	19	20	226	1	296	3	8	1	0	56	22	42	19

[1]n (%); Mean (SD) (Range)

Table S3. Weighted demographic summary by country.

Characteristic	Australia, N = 639[1]	Brazil, N = 553[1]	Burundi, N = 369[1]	Canada, N = 368[1]	Chile, N = 342[1]	Egypt, N = 461[1]	Guatemala, N = 229[1]	Hong Kong, N = 2,127[1]	India, N = 529[1]	Indonesia, N = 405[1]	Italy, N = 203[1]	Lebanon, N = 440[1]	Libya, N = 612[1]	Macau, N = 233[1]	Mainland China, N = 667[1]
Age															
18-24	59 (9·2%)	75 (13%)	80 (22%)	40 (11%)	34 (10·0%)	71 (15%)	41 (18%)	154 (7·2%)	80 (15%)	69 (17%)	12 (6·0%)	59 (13%)	91 (15%)	19 (8·3%)	101 (15%)
25-29	64 (10·0%)	74 (13%)	70 (19%)	30 (8·1%)	39 (11%)	71 (15%)	37 (16%)	176 (8·3%)	73 (14%)	69 (17%)	13 (6·4%)	51 (11%)	95 (15%)	22 (9·5%)	83 (12%)
30-34	63 (9·9%)	68 (12%)	54 (15%)	30 (8·3%)	38 (11%)	63 (14%)	31 (14%)	199 (9·4%)	66 (12%)	67 (17%)	14 (6·7%)	43 (9·9%)	94 (15%)	33 (14%)	76 (11%)
35-39	60 (9·3%)	60 (11%)	38 (10%)	30 (8·1%)	34 (9·9%)	56 (12%)	26 (11%)	197 (9·3%)	60 (11%)	66 (16%)	15 (7·3%)	40 (9·1%)	91 (15%)	29 (12%)	90 (13%)
40-44	53 (8·3%)	56 (10%)	30 (8·2%)	30 (8·0%)	32 (9·3%)	45 (9·7%)	21 (9·4%)	197 (9·2%)	54 (10%)	31 (7·7%)	17 (8·5%)	37 (8·3%)	77 (13%)	22 (9·6%)	100 (15%)
45-49	56 (8·8%)	51 (9·3%)	26 (6·9%)	31 (8·4%)	30 (8·8%)	38 (8·2%)	18 (7·7%)	196 (9·2%)	47 (9·0%)	57 (14%)	20 (9·7%)	36 (8·3%)	60 (9·8%)	22 (9·6%)	94 (14%)
50-54	51 (8·0%)	44 (7·9%)	22 (5·9%)	35 (9·5%)	29 (8·5%)	35 (7·5%)	14 (6·1%)	222 (10%)	39 (7·5%)	25 (6·2%)	20 (9·9%)	39 (8·9%)	42 (6·9%)	21 (9·0%)	58 (8·7%)
55-59	52 (8·1%)	36 (6·5%)	19 (5·0%)	34 (9·3%)	27 (7·9%)	28 (6·1%)	11 (4·9%)	215 (10%)	32 (6·0%)	21 (5·2%)	19 (9·3%)	34 (7·8%)	29 (4·7%)	24 (10%)	66 (10·0%)
60-64	46 (7·3%)	28 (5·1%)	12 (3·2%)	30 (8·1%)	23 (6·8%)	23 (5·0%)	9 (3·9%)	171 (8·0%)	25 (4·8%)	0 (0%)	16 (7·9%)	29 (6·5%)	9 (1·5%)	20 (8·8%)	0 (0%)
>=65	135 (21%)	61 (11%)	18 (4·9%)	78 (21%)	56 (16%)	32 (6·9%)	21 (9·4%)	401 (19%)	53 (10·0%)	0 (0%)	57 (28%)	72 (16%)	24 (3·9%)	19 (8·3%)	0 (0%)
Gender															
Female	324 (51%)	287 (52%)	178 (48%)	186 (50%)	173 (51%)	224 (49%)	117 (51%)	1,171 (55%)	260 (49%)	184 (45%)	105 (52%)	207 (47%)	287 (47%)	133 (57%)	327 (49%)
Male	312 (49%)	266 (48%)	172 (47%)	174 (47%)	164 (48%)	237 (51%)	107 (47%)	955 (45%)	269 (51%)	222 (55%)	98 (48%)	233 (53%)	326 (53%)	99 (43%)	334 (50%)
Non-binary	3 (0·5%)	0 (<0·1%)	18 (5·0%)	9 (2·3%)	5 (1·4%)	0 (0%)	4 (1·9%)	1 (<0·1%)	0 (0%)	0 (0%)	0 (0%)	0 (0%)	0 (0%)	0 (0%)	6 (0·9%)

Marital															
Single	188 (29%)	197 (36%)	155 (42%)	89 (24%)	139 (41%)	130 (28%)	116 (50%)	559 (26%)	147 (28%)	158 (39%)	35 (17%)	125 (28%)	268 (44%)	70 (30%)	200 (30%)
Married/Cohabitation/Common-law	350 (55%)	306 (55%)	190 (51%)	239 (65%)	144 (42%)	306 (66%)	109 (48%)	1,411 (66%)	333 (63%)	248 (61%)	142 (70%)	269 (61%)	324 (53%)	154 (66%)	411 (62%)
Separated/Divorced/Widowed	100 (16%)	50 (9·1%)	24 (6·5%)	39 (11%)	59 (17%)	25 (5·4%)	5 (2·0%)	156 (7·3%)	49 (9·2%)	0 (0%)	26 (13%)	46 (10%)	20 (3·3%)	9 (3·9%)	56 (8·3%)
Unknown	1	0	0	0	0	0	0	0	0	0	0	0	0	0	0
Education															
Primary or below	0 (0%)	2 (0·3%)	133 (36%)	0 (0·1%)	5 (1·4%)	1 (0·1%)	0 (0%)	371 (17%)	74 (14%)	0 (0%)	4 (2·2%)	37 (8·4%)	6 (1·0%)	0 (0%)	7 (1·1%)
Secondary	163 (27%)	57 (10%)	178 (48%)	51 (14%)	38 (11%)	23 (5·0%)	30 (13%)	1,072 (50%)	63 (12%)	0 (0%)	11 (5·3%)	49 (11%)	61 (10%)	12 (5·1%)	134 (20%)
Associate degree	119 (20%)	9 (1·7%)	14 (3·7%)	10 (2·8%)	4 (1·2%)	16 (3·4%)	2 (0·7%)	158 (7·4%)	44 (8·4%)	73 (18%)	66 (32%)	40 (9·2%)	51 (8·4%)	114 (49%)	17 (2·6%)
College	179 (30%)	53 (9·5%)	13 (3·6%)	104 (28%)	128 (37%)	45 (9·8%)	62 (27%)	143 (6·7%)	65 (13%)	27 (6·6%)	13 (6·2%)	43 (9·7%)	70 (11%)	9 (3·8%)	176 (27%)
Bachelor	79 (13%)	144 (26%)	24 (6·6%)	96 (26%)	47 (14%)	201 (44%)	44 (19%)	342 (16%)	102 (20%)	195 (48%)	17 (8·1%)	127 (29%)	284 (46%)	78 (34%)	158 (24%)
Graduate	55 (9·2%)	288 (52%)	6 (1·7%)	107 (29%)	119 (35%)	175 (38%)	92 (40%)	41 (1·9%)	173 (33%)	110 (27%)	93 (46%)	144 (33%)	139 (23%)	19 (8·2%)	163 (25%)
Unknown	44	0	0	0	0	0	0	0	7	0	0	0	0	0	12
Employment															
Job seeking	84 (13%)	9 (1·7%)	18 (5·0%)	12 (3·3%)	24 (7·1%)	17 (3·8%)	6 (2·6%)	60 (2·8%)	27 (5·0%)	10 (2·4%)	6 (2·9%)	38 (8·7%)	35 (5·7%)	1 (0·5%)	52 (7·8%)
Laid off	44 (6·9%)	1 (0·2%)	13 (3·6%)	8 (2·0%)	0 (0%)	0 (0%)	3 (1·2%)	36 (1·7%)	0 (<0·1%)	0 (0%)	0 (0%)	7 (1·5%)	1 (0·2%)	0 (0%)	22 (3·2%)
Not in workforce	44 (6·9%)	8 (1·4%)	3 (0·9%)	34 (9·2%)	14 (4·0%)	34 (7·3%)	0 (0%)	231 (11%)	95 (18%)	3 (0·7%)	7 (3·6%)	63 (14%)	34 (5·5%)	3 (1·2%)	18 (2·8%)

Retired	58 (9·0%)	63 (11%)	3 (0·8%)	91 (25%)	52 (15%)	48 (10%)	12 (5·1%)	437 (21%)	22 (4·3%)	0 (0%)	62 (30%)	5 (1·2%)	24 (3·9%)	23 (9·7%)	35 (5·3%)
Self-employed	7 (1·1%)	63 (11%)	179 (48%)	20 (5·4%)	26 (7·7%)	68 (15%)	34 (15%)	37 (1·7%)	78 (15%)	73 (18%)	21 (10%)	108 (25%)	58 (9·4%)	14 (6·2%)	40 (6·1%)
Student	39 (6·1%)	83 (15%)	82 (22%)	25 (6·9%)	50 (15%)	64 (14%)	57 (25%)	127 (6·0%)	92 (17%)	90 (22%)	12 (6·1%)	49 (11%)	114 (19%)	16 (6·7%)	102 (15%)
Working (>=40hrs/wk)	66 (10%)	239 (43%)	22 (6·1%)	82 (22%)	143 (42%)	129 (28%)	78 (34%)	885 (42%)	158 (30%)	112 (28%)	46 (22%)	77 (18%)	111 (18%)	89 (38%)	280 (42%)
Working (1-39hrs/wk)	297 (46%)	87 (16%)	47 (13%)	97 (26%)	32 (9·4%)	100 (22%)	40 (17%)	313 (15%)	57 (11%)	117 (29%)	49 (24%)	92 (21%)	236 (39%)	87 (37%)	118 (18%)
Height (m)	1·70 (0·08) (1·50 to 1·96)	1·69 (0·11) (1·16 to 1·94)	1·63 (0·06) (1·52 to 1·90)	1·70 (0·12) (1·28 to 2·11)	1·67 (0·09) (1·40 to 1·90)	1·68 (0·10) (1·30 to 1·97)	1·66 (0·10) (1·44 to 1·98)	1·64 (0·08) (1·37 to 1·98)	1·63 (0·10) (1·43 to 1·96)	1·62 (0·07) (1·45 to 1·84)	1·70 (0·09) (1·50 to 1·94)	1·68 (0·09) (1·47 to 1·98)	1·66 (0·14) (1·20 to 2·00)	1·65 (0·08) (1·49 to 1·98)	1·67 (0·08) (1·30 to 1·86)
Unknown	14	0	1	5	0	0	1	1	4	0	0	1	3	0	5
Weight(kg)	74 (14) (27 to 130)	74 (16) (37 to 120)	60 (7) (45 to 89)	79 (19) (31 to 127)	75 (13) (41 to 113)	80 (17) (40 to 160)	69 (14) (39 to 110)	62 (10) (39 to 117)	67 (12) (34 to 104)	64 (13) (33 to 103)	72 (16) (45 to 122)	75 (15) (40 to 140)	76 (18) (36 to 150)	63 (12) (30 to 110)	66 (14) (35 to 135)
Unknown	8	0	0	26	0	0	1	0	5	0	0	0	0	0	5
BMI	25·6 (4·2) (11·7 to 45·8)	26·0 (4·5) (15·6 to 42·0)	22·4 (2·2) (15·5 to 34·0)	27·0 (5·2) (13·5 to 43·8)	26·7 (3·8) (17·6 to 44·4)	28·4 (5·3) (15·4 to 62·4)	24·8 (3·9) (13·9 to 37·1)	23·1 (3·0) (13·4 to 38·2)	25·1 (3·7) (14·5 to 36·1)	24·1 (4·5) (14·1 to 40·3)	24·9 (4·4) (17·3 to 38·9)	26·6 (4·3) (13·8 to 48·4)	27·9 (7·5) (12·9 to 52·1)	22·9 (3·5) (11·0 to 37·5)	23·5 (4·2) (12·1 to 48·4)
Unknown	16	0	1	28	0	0	2	1	5	0	0	1	3	0	5
BMI classification															
Severely underweight	6 (1·0%)	2 (0·3%)	4 (1·0%)	1 (0·4%)	0 (0%)	1 (0·2%)	5 (2·0%)	7 (0·3%)	3 (0·5%)	3 (0·7%)	0 (0%)	0 (<0·1%)	5 (0·7%)	5 (2·3%)	4 (0·6%)
Underweight	16 (2·6%)	12 (2·2%)	3 (0·9%)	5 (1·4%)	2 (0·5%)	6 (1·2%)	2 (0·8%)	101 (4·7%)	12 (2·4%)	36 (8·9%)	4 (1·8%)	10 (2·2%)	12 (2·0%)	17 (7·5%)	41 (6·2%)
Normal weight	260 (42%)	238 (43%)	336 (91%)	120 (35%)	105 (31%)	51 (11%)	115 (51%)	868 (41%)	134 (26%)	144 (35%)	114 (56%)	76 (17%)	221 (36%)	86 (37%)	264 (40%)

Overweight	248 (40%)	194 (35%)	18 (4·9%)	128 (38%)	171 (50%)	68 (15%)	84 (37%)	634 (30%)	106 (20%)	59 (15%)	63 (31%)	77 (18%)	206 (34%)	62 (27%)	172 (26%)
Obesity	93 (15%)	108 (19%)	7 (1·8%)	85 (25%)	65 (19%)	336 (73%)	21 (9·2%)	516 (24%)	268 (51%)	164 (40%)	23 (11%)	276 (63%)	165 (27%)	62 (27%)	182 (27%)
Unknown	16	0	1	28	0	0	2	1	5	0	0	1	3	0	5
Pregnant															
Not applicable	313 (49%)	266 (48%)	172 (47%)	174 (47%)	164 (48%)	254 (55%)	107 (47%)	955 (45%)	269 (51%)	222 (55%)	98 (48%)	238 (54%)	326 (53%)	99 (43%)	335 (50%)
No	321 (50%)	283 (51%)	164 (44%)	185 (50%)	176 (52%)	207 (45%)	119 (52%)	1,165 (55%)	259 (49%)	176 (43%)	104 (51%)	200 (45%)	273 (45%)	106 (45%)	257 (39%)
Yes	5 (0·8%)	4 (0·8%)	33 (9·0%)	9 (2·5%)	1 (0·4%)	0 (0%)	3 (1·1%)	7 (0·3%)	1 (0·1%)	8 (2·0%)	0 (0·2%)	3 (0·6%)	14 (2·2%)	28 (12%)	75 (11%)
Gestational week	20 (12) (6 to 40)	7 (0) (6 to 7)	5 (1) (3 to 7)	25 (11) (4 to 39)	30 (0) (30 to 30)	0 (0) (Inf to -Inf)	0 (0) (Inf to -Inf)	25 (9) (15 to 40)	0 (0) (Inf to -Inf)	11 (4) (8 to 32)	12 (0) (12 to 12)	17 (11) (6 to 30)	24 (11) (9 to 36)	38 (7) (5 to 40)	35 (7) (4 to 41)
Unknown	634	552	354	359	341	461	229	2,120	529	397	203	437	607	205	643
Regular medical follow-up before COVID-19															
No	435 (68%)	374 (68%)	340 (92%)	166 (45%)	199 (58%)	316 (69%)	157 (69%)	1,363 (64%)	320 (61%)	261 (65%)	53 (26%)	223 (51%)	427 (70%)	156 (67%)	570 (85%)
Yes	204 (32%)	179 (32%)	29 (7·8%)	202 (55%)	143 (42%)	145 (31%)	72 (31%)	764 (36%)	209 (39%)	144 (35%)	150 (74%)	217 (49%)	185 (30%)	77 (33%)	97 (15%)
Unknown	1	0	0	0	0	0	0	0	0	0	0	0	0	0	0
Practising healthcare professional															
No	557 (87%)	365 (66%)	354 (96%)	345 (94%)	303 (89%)	249 (54%)	166 (73%)	2,065 (97%)	389 (73%)	225 (55%)	189 (93%)	311 (71%)	420 (69%)	117 (50%)	619 (93%)
Yes	82 (13%)	188 (34%)	15 (4·2%)	23 (6·3%)	39 (11%)	212 (46%)	63 (27%)	62 (2·9%)	140 (27%)	181 (45%)	14 (7·0%)	129 (29%)	192 (31%)	116 (50%)	48 (7·2%)
Unknown	0	0	0	0	0	0	0	0	0	0	0	0	0	0	0

Number of children less than 18 years old															
No	526 (82%)	397 (72%)	193 (52%)	270 (73%)	240 (70%)	233 (50%)	172 (75%)	1,591 (75%)	325 (61%)	255 (63%)	152 (75%)	284 (65%)	372 (61%)	125 (54%)	360 (54%)
Yes	113 (18%)	156 (28%)	176 (48%)	98 (27%)	102 (30%)	228 (50%)	57 (25%)	536 (25%)	204 (39%)	151 (37%)	51 (25%)	156 (35%)	240 (39%)	107 (46%)	307 (46%)
Number of children aged above 18	0·30 (0·70) (0·00 to 3·00)	0·43 (0·86) (0·00 to 8·00)	1·00 (1·21) (0·00 to 6·00)	0·49 (0·95) (0·00 to 5·00)	0·45 (0·83) (0·00 to 5·00)	1·03 (1·25) (0·00 to 5·00)	0·41 (0·82) (0·00 to 5·00)	0·34 (0·64) (0·00 to 4·00)	0·59 (0·86) (0·00 to 5·00)	0·44 (0·62) (0·00 to 2·00)	0·40 (0·76) (0·00 to 3·00)	0·84 (1·26) (0·00 to 5·00)	1·13 (1·66) (0·00 to 6·00)	0·79 (1·00) (0·00 to 7·00)	0·57 (0·73) (0·00 to 6·00)
Unknown	0	10	0	1	4	10	3	0	1	0	0	0	12	0	10
Number of people lived with	2·74 (1·19) (1·00 to 6·00)	2·77 (1·28) (1·00 to 7·00)	4·11 (1·46) (1·00 to 8·00)	2·46 (1·31) (1·00 to 7·00)	3·01 (1·41) (1·00 to 8·00)	4·50 (1·85) (1·00 to 17·00)	4·01 (2·09) (1·00 to 12·00)	3·27 (1·13) (1·00 to 9·00)	3·76 (1·69) (1·00 to 20·00)	4·38 (1·31) (1·00 to 9·00)	2·51 (1·18) (1·00 to 6·00)	4·25 (1·45) (1·00 to 11·00)	5·90 (2·97) (1·00 to 26·00)	3·87 (1·60) (1·00 to 12·00)	3·22 (1·17) (1·00 to 10·00)
Unknown	41	0	4	1	0	3	0	0	2	0	1	0	16	0	49
Perceived social rank	3·15 (0·93) (1·00 to 5·00)	3·51 (0·83) (1·00 to 5·00)	1·69 (0·76) (1·00 to 4·00)	3·43 (0·97) (1·00 to 5·00)	3·19 (0·85) (1·00 to 5·00)	3·41 (0·93) (1·00 to 5·00)	3·26 (0·73) (1·00 to 5·00)	2·69 (0·78) (1·00 to 5·00)	2·97 (1·16) (1·00 to 5·00)	3·45 (0·83) (1·00 to 5·00)	3·57 (0·72) (2·00 to 5·00)	2·99 (0·78) (1·00 to 5·00)	3·25 (1·10) (1·00 to 5·00)	3·41 (0·70) (1·00 to 5·00)	2·81 (0·79) (1·00 to 5·00)
Unknown	1	0	0	0	0	0	0	0	0	11	0	0	0	0	1
House size	175 (141) (10 to 575)	137 (89) (10 to 500)	85 (31) (10 to 202)	137 (84) (3 to 550)	92 (51) (10 to 400)	119 (56) (10 to 400)	163 (118) (10 to 550)	39 (19) (9 to 195)	53 (61) (1 to 581)	194 (117) (10 to 590)	115 (47) (40 to 315)	135 (66) (10 to 570)	244 (144) (11 to 500)	140 (117) (10 to 570)	105 (46) (11 to 400)
Unknown	431	29	2	113	31	2	23	1	30	0	13	21	281	0	5

(Continued)

Characteristic	Malaysia, N = 535[1]	Mexico, N = 1,016[1]	Nigeria, N = 580[1]	Philippines, N = 457[1]	Republic Of Sudan, N = 538[1]	Rwanda, N = 136[1]	Saudi Arabia, N = 609[1]	Singapore, N = 237[1]	South Africa, N = 192[1]	South Korea, N = 2,238[1]	Spain, N = 45[1]	Thailand, N = 723[1]	United Kingdom, N = 212[1]	United States, N = 184[1]	Vietnam, N = 401[1]
Age															
18-24	75 (14%)	127 (13%)	127 (22%)	74 (16%)	108 (20%)	29 (21%)	70 (12%)	24 (10%)	28 (15%)	185 (8·3%)	3 (7·6%)	70 (9·7%)	18 (8·3%)	19 (10%)	51 (13%)
25-29	78 (15%)	122 (12%)	116 (20%)	65 (14%)	90 (17%)	26 (19%)	88 (14%)	20 (8·6%)	31 (16%)	185 (8·3%)	2 (4·1%)	76 (11%)	19 (9·0%)	20 (11%)	64 (16%)
30-34	69 (13%)	115 (11%)	90 (15%)	57 (13%)	74 (14%)	24 (18%)	89 (15%)	21 (8·9%)	33 (17%)	179 (8·0%)	2 (4·5%)	81 (11%)	19 (8·8%)	19 (10%)	64 (16%)
35-39	62 (12%)	110 (11%)	69 (12%)	53 (11%)	69 (13%)	21 (15%)	100 (16%)	21 (9·0%)	28 (14%)	217 (9·7%)	2 (5·4%)	84 (12%)	18 (8·5%)	19 (10%)	59 (15%)
40-44	48 (9·0%)	104 (10%)	61 (11%)	46 (10·0%)	53 (9·9%)	15 (11%)	89 (15%)	21 (9·0%)	22 (11%)	210 (9·4%)	6 (13%)	85 (12%)	17 (7·9%)	17 (9·3%)	51 (13%)
45-49	43 (8·0%)	97 (9·5%)	44 (7·5%)	41 (9·0%)	46 (8·6%)	5 (3·6%)	66 (11%)	22 (9·4%)	18 (9·6%)	242 (11%)	6 (13%)	79 (11%)	19 (9·0%)	18 (9·8%)	48 (12%)
50-54	39 (7·4%)	86 (8·5%)	40 (6·9%)	35 (7·6%)	32 (6·0%)	4 (3·0%)	46 (7·6%)	21 (8·9%)	15 (7·8%)	219 (9·8%)	5 (12%)	69 (9·5%)	20 (9·2%)	18 (9·8%)	43 (11%)
55-59	35 (6·6%)	70 (6·8%)	20 (3·4%)	28 (6·2%)	25 (4·7%)	8 (6·0%)	32 (5·2%)	22 (9·2%)	13 (6·7%)	230 (10%)	5 (11%)	53 (7·4%)	18 (8·4%)	19 (10%)	21 (5·2%)
60-64	29 (5·4%)	59 (5·8%)	13 (2·2%)	22 (4·7%)	17 (3·2%)	4 (2·7%)	13 (2·2%)	20 (8·5%)	4 (2·3%)	180 (8·0%)	0 (0%)	41 (5·7%)	15 (7·2%)	9 (5·0%)	0 (0%)
>=65	55 (10%)	126 (12%)	0 (0%)	37 (8·2%)	23 (4·3%)	0 (0%)	16 (2·6%)	44 (18%)	0 (0%)	391 (17%)	13 (30%)	86 (12%)	51 (24%)	25 (14%)	0 (0%)
Gender															
Female	260 (49%)	529 (52%)	287 (49%)	223 (49%)	262 (49%)	65 (48%)	225 (37%)	122 (52%)	95 (49%)	1,121 (50%)	26 (58%)	366 (51%)	109 (51%)	109 (59%)	208 (52%)
Male	274 (51%)	479 (47%)	290 (50%)	225 (49%)	276 (51%)	66 (49%)	384 (63%)	104 (44%)	97 (50%)	1,110 (50%)	18 (41%)	344 (48%)	103 (49%)	72 (39%)	190 (48%)
Non-binary	1 (0·2%)	8 (0·8%)	3 (0·4%)	9 (2·0%)	0 (0%)	5 (3·5%)	0 (0%)	11 (4·7%)	0 (<0·1%)	6 (0·3%)	0 (1·1%)	14 (1·9%)	0 (0·2%)	3 (1·8%)	2 (0·5%)

Marital															
Single	159 (30%)	433 (43%)	281 (48%)	208 (46%)	254 (47%)	59 (43%)	174 (29%)	93 (39%)	77 (40%)	686 (31%)	9 (19%)	424 (59%)	45 (21%)	64 (35%)	105 (26%)
Married/Cohabitation/Common-law	353 (66%)	432 (43%)	278 (48%)	220 (48%)	244 (45%)	70 (51%)	411 (67%)	137 (58%)	106 (55%)	1,427 (64%)	32 (71%)	256 (35%)	146 (69%)	108 (59%)	284 (71%)
Separated/Divorced/Widowed	24 (4·5%)	151 (15%)	20 (3·5%)	29 (6·3%)	40 (7·5%)	8 (5·7%)	24 (3·9%)	7 (3·0%)	9 (4·7%)	125 (5·6%)	4 (9·6%)	43 (6·0%)	20 (9·6%)	12 (6·4%)	12 (2·9%)
Unknown	0	0	0	0	0	0	0	0	0	0	0	0	0	0	0
Education															
Primary or below	24 (4·5%)	14 (1·4%)	2 (0·4%)	2 (0·4%)	24 (4·4%)	0 (0%)	4 (0·7%)	1 (0·3%)	0 (0%)	2 (<0·1%)	0 (0%)	14 (1·9%)	0 (0%)	3 (3·0%)	0 (0%)
Secondary	57 (11%)	95 (9·4%)	23 (3·9%)	23 (5·0%)	52 (9·7%)	31 (23%)	86 (14%)	2 (0·9%)	27 (14%)	30 (1·3%)	3 (6·4%)	17 (2·4%)	12 (5·7%)	1 (1·2%)	9 (2·3%)
Associate degree	143 (27%)	3 (0·3%)	16 (2·8%)	10 (2·3%)	25 (4·6%)	8 (6·0%)	13 (2·1%)	1 (0·6%)	2 (1·3%)	316 (14%)	1 (1·1%)	22 (3·0%)	2 (1·2%)	3 (2·8%)	36 (9·0%)
College	112 (21%)	122 (12%)	143 (25%)	74 (16%)	26 (4·8%)	10 (7·2%)	26 (4·3%)	37 (16%)	22 (11%)	464 (21%)	16 (37%)	6 (0·9%)	52 (25%)	10 (10%)	22 (5·5%)
Bachelor	153 (29%)	320 (32%)	249 (43%)	216 (47%)	284 (53%)	47 (34%)	324 (53%)	132 (56%)	89 (46%)	1,165 (52%)	4 (7·9%)	199 (28%)	62 (29%)	22 (23%)	188 (47%)
Graduate	44 (8·3%)	461 (45%)	147 (25%)	133 (29%)	128 (24%)	40 (30%)	154 (25%)	63 (27%)	52 (27%)	261 (12%)	21 (48%)	462 (64%)	82 (39%)	57 (60%)	145 (36%)
Unknown	2	0	0	0	0	0	2	0	0	0	0	3	2	89	0
Employment															
Job seeking	15 (2·9%)	28 (2·7%)	49 (8·5%)	19 (4·2%)	34 (6·3%)	13 (9·2%)	50 (8·2%)	1 (0·3%)	8 (4·1%)	93 (4·1%)	3 (6·6%)	22 (3·1%)	4 (1·8%)	1 (0·6%)	8 (2·0%)
Laid off	1 (0·2%)	7 (0·7%)	14 (2·4%)	0 (0%)	14 (2·5%)	1 (0·8%)	6 (1·0%)	0 (0%)	1 (0·6%)	9 (0·4%)	0 (0%)	4 (0·5%)	5 (2·4%)	0 (0%)	0 (0%)
Not in workforce	35 (6·5%)	24 (2·4%)	4 (0·7%)	23 (4·9%)	82 (15%)	1 (0·5%)	60 (9·8%)	2 (1·0%)	3 (1·3%)	396 (18%)	0 (0%)	7 (1·0%)	3 (1·6%)	2 (1·2%)	0 (0%)

Retired	68 (13%)	60 (5·9%)	0 (0%)	32 (7·1%)	3 (0·6%)	0 (0%)	46 (7·5%)	22 (9·1%)	4 (1·9%)	87 (3·9%)	10 (22%)	97 (13%)	31 (15%)	15 (8·2%)	37 (9·3%)
Self-employed	53 (10·0%)	132 (13%)	28 (4·8%)	61 (13%)	56 (10%)	12 (8·7%)	11 (1·7%)	4 (1·6%)	41 (21%)	324 (14%)	8 (18%)	75 (10%)	8 (4·0%)	12 (6·6%)	20 (5·0%)
Student	99 (18%)	145 (14%)	134 (23%)	70 (15%)	131 (24%)	23 (17%)	81 (13%)	42 (18%)	32 (16%)	150 (6·7%)	4 (10%)	89 (12%)	32 (15%)	21 (11%)	49 (12%)
Working (>=40hrs/wk)	203 (38%)	409 (40%)	221 (38%)	202 (44%)	82 (15%)	64 (47%)	191 (31%)	142 (60%)	83 (43%)	891 (40%)	12 (27%)	307 (43%)	54 (25%)	87 (47%)	217 (54%)
Working (1-39hrs/wk)	60 (11%)	211 (21%)	129 (22%)	50 (11%)	137 (25%)	23 (17%)	165 (27%)	25 (11%)	22 (11%)	289 (13%)	8 (17%)	121 (17%)	74 (35%)	45 (25%)	69 (17%)
Height (m)	1·62 (0·08) (1·40 to 1·85)	1·66 (0·10) (1·23 to 2·10)	1·65 (0·17) (1·20 to 2·20)	1·61 (0·11) (1·15 to 1·96)	1·67 (0·12) (1·12 to 2·00)	1·66 (0·09) (1·40 to 1·93)	1·68 (0·09) (1·35 to 1·95)	1·67 (0·11) (1·48 to 1·85)	1·65 (0·13) (1·10 to 1·96)	1·66 (0·08) (1·40 to 1·90)	1·69 (0·07) (1·50 to 1·85)	1·65 (0·08) (1·43 to 1·85)	1·69 (0·10) (1·25 to 1·95)	1·68 (0·07) (1·45 to 1·80)	1·61 (0·07) (1·45 to 1·84)
Unknown	0	2	20	5	9	6	2	0	1	0	0	1	0	15	0
Weight(kg)	66 (12) (35 to 98)	72 (15) (40 to 120)	64 (12) (25 to 100)	67 (15) (21 to 112)	72 (16) (35 to 120)	67 (12) (32 to 97)	77 (18) (35 to 145)	68 (13) (39 to 103)	71 (19) (40 to 140)	65 (12) (32 to 137)	73 (13) (49 to 100)	65 (14) (37 to 108)	75 (20) (16 to 160)	76 (16) (40 to 134)	58 (9) (38 to 88)
Unknown	0	3	4	0	0	0	2	0	0	0	0	3	0	14	0
BMI	25·1 (4·3) (14·3 to 39·4)	26·2 (4·3) (13·8 to 39·4)	24·4 (6·6) (11·2 to 51·7)	25·8 (5·2) (7·5 to 47·4)	25·9 (5·1) (10·8 to 38·3)	24·6 (3·7) (12·9 to 32·9)	27·3 (5·3) (12·9 to 53·5)	24·5 (4·0) (15·2 to 36·9)	25·9 (6·7) (14·3 to 56·7)	23·3 (3·3) (13·3 to 45·7)	25·6 (3·9) (18·0 to 36·3)	23·6 (4·2) (14·1 to 38·9)	26·3 (7·0) (6·2 to 50·6)	27·1 (5·9) (16·9 to 50·2)	22·4 (2·4) (14·7 to 32·9)
Unknown	0	5	22	5	9	6	2	0	2	0	0	3	0	17	0
BMI classification															
Severely underweight	14 (2·7%)	9 (0·9%)	28 (5·1%)	7 (1·4%)	9 (1·7%)	2 (1·6%)	4 (0·6%)	0 (0·1%)	2 (1·1%)	9 (0·4%)	0 (0%)	12 (1·7%)	8 (3·8%)	0 (0%)	1 (0·3%)
Underweight	13 (2·4%)	16 (1·6%)	40 (7·1%)	19 (4·1%)	20 (3·8%)	0 (0·3%)	15 (2·5%)	7 (3·1%)	14 (7·3%)	101 (4·5%)	1 (1·5%)	43 (6·0%)	4 (1·8%)	1 (0·6%)	20 (4·9%)

Normal weight	159 (30%)	370 (37%)	289 (52%)	122 (27%)	211 (40%)	70 (54%)	91 (15%)	97 (41%)	78 (41%)	983 (44%)	22 (49%)	304 (42%)	89 (42%)	65 (38%)	212 (53%)
Overweight	92 (17%)	434 (43%)	117 (21%)	65 (14%)	153 (29%)	47 (36%)	93 (15%)	37 (16%)	55 (29%)	530 (24%)	15 (32%)	134 (19%)	63 (30%)	68 (41%)	120 (30%)
Obesity	257 (48%)	182 (18%)	84 (15%)	239 (53%)	136 (26%)	11 (8·2%)	404 (67%)	96 (40%)	41 (22%)	614 (27%)	7 (17%)	226 (31%)	47 (22%)	34 (20%)	48 (12%)
Unknown	0	5	22	5	9	6	2	0	2	0	0	3	0	17	0
Pregnant															
Not applicable	274 (51%)	479 (47%)	290 (50%)	225 (49%)	276 (51%)	66 (49%)	384 (63%)	104 (44%)	97 (50%)	1,117 (50%)	18 (41%)	344 (48%)	103 (49%)	72 (39%)	190 (48%)
No	250 (47%)	533 (52%)	261 (45%)	229 (50%)	253 (47%)	63 (46%)	213 (35%)	132 (56%)	95 (49%)	1,115 (50%)	26 (59%)	377 (52%)	109 (51%)	110 (60%)	205 (51%)
Yes	11 (2·1%)	4 (0·4%)	28 (4·9%)	4 (0·8%)	9 (1·7%)	7 (5·3%)	12 (2·0%)	1 (0·6%)	0 (<0·1%)	6 (0·3%)	0 (0%)	2 (0·3%)	0 (0%)	2 (0·8%)	5 (1·4%)
Gestational week	24 (10) (12 to 35)	24 (0) (24 to 24)	26 (5) (17 to 34)	0 (0) (Inf to -Inf)	6 (0) (6 to 8)	6 (2) (4 to 9)	14 (10) (5 to 30)	10 (0) (10 to 10)	0 (0) (Inf to -Inf)	9 (2) (5 to 10)	0 (0) (Inf to -Inf)	17 (13) (6 to 36)	0 (0) (Inf to -Inf)	29 (4) (25 to 34)	31 (7) (15 to 36)
Unknown	527	1,015	574	457	529	133	606	236	192	2,232	45	721	212	183	397
Regular medical follow-up before COVID-19															
No	339 (63%)	595 (59%)	409 (70%)	325 (71%)	407 (76%)	118 (86%)	412 (68%)	127 (54%)	131 (68%)	1,264 (56%)	24 (54%)	405 (56%)	104 (49%)	73 (40%)	69 (17%)
Yes	196 (37%)	421 (41%)	171 (30%)	132 (29%)	131 (24%)	19 (14%)	198 (32%)	110 (46%)	61 (32%)	974 (44%)	20 (46%)	318 (44%)	108 (51%)	109 (60%)	332 (83%)
Unknown	0	0	0	0	0	0	0	0	0	0	0	0	0	2	0
Practising healthcare professional															
No	344 (64%)	387 (38%)	265 (46%)	323 (71%)	378 (70%)	73 (53%)	433 (71%)	101 (42%)	127 (66%)	2,163 (97%)	40 (90%)	649 (90%)	140 (66%)	88 (47%)	175 (44%)
Yes	191 (36%)	629 (62%)	315 (54%)	134 (29%)	160 (30%)	64 (47%)	176 (29%)	136 (58%)	65 (34%)	75 (3·4%)	4 (10·0%)	74 (10%)	72 (34%)	97 (53%)	226 (56%)

Unknown	0	0	0	0	0	0	0	0	0	0	0	0	0	0	0
Number of children less than 18 years old															
No	320 (60%)	756 (74%)	358 (62%)	283 (62%)	350 (65%)	72 (53%)	254 (42%)	165 (70%)	138 (72%)	1,646 (74%)	30 (68%)	613 (85%)	153 (72%)	134 (72%)	146 (36%)
Yes	215 (40%)	260 (26%)	221 (38%)	174 (38%)	188 (35%)	64 (47%)	356 (58%)	72 (30%)	54 (28%)	592 (26%)	14 (32%)	110 (15%)	59 (28%)	51 (28%)	255 (64%)
Number of children aged above 18	0·92 (1·42) (0·00 to 8·00)	0·44 (0·98) (0·00 to 11·00)	1·03 (1·71) (0·00 to 10·00)	0·66 (1·01) (0·00 to 4·00)	0·96 (1·53) (0·00 to 6·00)	1·00 (1·31) (0·00 to 5·00)	1·62 (1·74) (0·00 to 7·00)	0·59 (0·98) (0·00 to 4·00)	0·45 (0·82) (0·00 to 3·00)	0·42 (0·76) (0·00 to 5·00)	0·55 (0·88) (0·00 to 3·00)	0·21 (0·56) (0·00 to 4·00)	0·46 (0·88) (0·00 to 6·00)	0·44 (0·81) (0·00 to 4·00)	1·08 (0·95) (0·00 to 4·00)
Unknown	0	14	5	3	10	0	17	0	0	0	0	2	2	0	0
Number of people lived with	4·63 (2·24) (1·00 to 16·00)	3·38 (1·76) (1·00 to 14·00)	4·74 (2·92) (1·00 to 23·00)	4·91 (2·30) (1·00 to 14·00)	6·28 (2·75) (1·00 to 18·00)	4·41 (2·22) (1·00 to 10·00)	6·00 (2·67) (1·00 to 21·00)	3·97 (1·61) (1·00 to 10·00)	3·96 (2·01) (1·00 to 12·00)	3·02 (1·13) (1·00 to 7·00)	2·98 (1·50) (1·00 to 6·00)	3·32 (1·83) (1·00 to 18·00)	2·67 (1·33) (1·00 to 8·00)	2·61 (1·26) (1·00 to 8·00)	4·18 (1·57) (1·00 to 12·00)
Unknown	1	4	5	5	49	0	28	0	3	0	0	1	0	16	1
Perceived social rank	3·55 (0·84) (1·00 to 5·00)	3·13 (0·66) (1·00 to 5·00)	3·20 (0·97) (1·00 to 5·00)	3·22 (0·71) (1·00 to 5·00)	3·16 (0·96) (1·00 to 5·00)	2·76 (0·80) (1·00 to 5·00)	3·45 (1·01) (1·00 to 5·00)	3·33 (0·73) (1·00 to 5·00)	3·46 (0·87) (1·00 to 5·00)	2·96 (0·70) (1·00 to 5·00)	3·33 (0·78) (1·00 to 5·00)	3·98 (0·83) (1·00 to 5·00)	3·43 (0·79) (1·00 to 5·00)	3·85 (0·83) (1·00 to 5·00)	3·21 (0·76) (1·00 to 5·00)
Unknown	0	0	0	0	0	0	0	0	0	0	0	0	0	11	0
House size	154 (136) (1 to 594)	137 (100) (10 to 500)	73 (77) (1 to 555)	121 (104) (1 to 561)	253 (145) (10 to 500)	104 (44) (12 to 249)	251 (157) (10 to 555)	102 (68) (1 to 500)	169 (122) (1 to 550)	32 (13) (10 to 100)	139 (71) (25 to 300)	151 (128) (10 to 540)	138 (119) (1 to 500)	196 (111) (20 to 560)	186 (143) (10 to 650)

Unknown	19	52	24	22	168	0	245	1	5	1	0	43	17	35	7

[1]n (%); Mean (SD) (Range)

Table S4. Description of respondents' weighted perception of COVID-19's impact and preference for future preparations by country.

Impact and preparation	All samples			Australia			Brazil			Burundi			Canada			Chile			Egypt			Guatemala			Hong Kong		
	Mean	SE	SD	Mean	SE	SD	Mean	SE	SD	Mean	SE	SD	Mean	SE	SD	Mean	SE	SD	Mean	SE	SD	Mean	SE	SD	Mean	SE	SD
Perception of COVID-19' impact																											
Food types in daily meals	-0·02	0·01	0·86	-0·29	0·03	0·75	0·19	0·05	0·90	-0·50	0·07	0·98	-0·03	0·05	0·81	0·45	0·06	0·85	-0·04	0·04	0·73	0·23	0·09	0·97	0·14	0·02	0·69
Consumption of fruits and vegetables	0·15	0·01	0·89	-0·66	0·03	0·73	0·16	0·05	0·87	-0·45	0·06	0·93	-0·11	0·05	0·79	0·42	0·07	0·89	0·15	0·06	0·88	0·24	0·08	0·99	0·29	0·02	0·69
Consumption of frozen food/food products	-0·01	0·01	0·96	-0·02	0·04	0·92	-0·22	0·05	1·02	-0·52	0·06	0·90	0·18	0·08	0·79	0·11	0·06	0·87	-0·68	0·07	0·98	-0·04	0·08	1·01	0·40	0·02	0·75
Consumption of snacks	-0·09	0·01	1·00	0·43	0·03	0·70	-0·29	0·06	1·08	-0·17	0·07	0·93	0·31	0·11	0·95	-0·13	0·11	1·26	-0·56	0·06	0·95	-0·01	0·10	1·16	0·05	0·02	0·84
Soft drinks/juices/other sugary drinks	-0·26	0·01	1·04	0·42	0·04	0·80	-0·21	0·06	1·13	-0·41	0·07	0·96	-0·23	0·10	0·85	-0·17	0·10	1·19	-0·89	0·07	1·03	-0·40	0·12	1·20	0·03	0·02	0·84
Having a meal at home	0·84	0·01	1·00	0·96	0·04	0·81	1·03	0·05	0·97	0·02	0·07	0·97	0·72	0·07	0·95	1·37	0·08	0·88	0·58	0·07	0·98	1·35	0·08	0·96	0·87	0·02	0·82
Cooking at home	0·78	0·01	1·00	1·04	0·03	0·62	0·95	0·05	1·01	0·09	0·06	0·89	0·61	0·07	0·91	1·28	0·08	0·94	0·54	0·06	0·97	1·29	0·08	0·96	0·87	0·02	0·81
Eating takeout food	-0·08	0·02	1·19	0·09	0·05	1·08	0·24	0·06	1·15	-0·40	0·06	0·88	-0·02	0·09	1·11	-0·19	0·11	1·32	-1·05	0·07	1·01	-0·06	0·11	1·23	0·32	0·02	0·91
Taking TCM or natural health products	-0·09	0·01	0·90	-0·71	0·02	0·52	0·24	0·05	0·85	-0·06	0·06	0·92	0·11	0·06	0·58	0·22	0·06	0·85	-0·43	0·11	1·15	0·34	0·11	1·09	0·01	0·01	0·47
Taking oral supplements/vitamins	0·07	0·01	0·94	-0·07	0·04	1·01	0·43	0·05	0·88	-0·20	0·07	0·95	0·16	0·06	0·68	0·11	0·09	0·96	-0·39	0·09	1·19	0·51	0·11	1·04	0·15	0·01	0·51
Smoking tobacco	-0·38	0·01	0·95	0·34	0·04	0·74	-0·28	0·05	0·91	-0·09	0·05	0·64	-0·08	0·04	0·56	-0·18	0·07	1·03	-1·02	0·08	1·10	-0·68	0·10	1·00	-0·01	0·01	0·45
Alcohol consumption	-0·36	0·01	0·95	0·31	0·03	0·71	-0·08	0·05	0·92	0·07	0·06	0·91	0·07	0·12	0·95	0·09	0·06	0·95	-1·17	0·07	1·00	-0·43	0·11	1·12	-0·11	0·01	0·58
Duration of sitting	0·59	0·01	0·97	1·27	0·03	0·67	1·01	0·05	0·96	0·12	0·07	0·97	0·92	0·07	0·84	1·38	0·06	0·82	0·01	0·06	1·02	1·11	0·07	0·91	0·60	0·02	0·73

Duration of screen time	0·67	0·01	0·97	1·20	0·03	0·58	1·05	0·06	0·99	0·00	0·07	1·07	0·95	0·07	0·82	1·47	0·06	0·81	0·07	0·06	1·02	1·31	0·07	0·87	0·59	0·02	0·74
Frequency of exercise	-0·21	0·01	1·06	0·25	0·05	1·04	-0·42	0·07	1·30	0·01	0·07	1·01	-0·49	0·08	1·08	-0·68	0·09	1·18	-0·60	0·09	1·07	-0·22	0·11	1·28	-0·28	0·02	0·83
Duration of exercise	-0·25	0·01	1·04	0·28	0·04	0·92	-0·40	0·07	1·23	-0·24	0·07	1·04	-0·48	0·08	1·07	-0·73	0·09	1·16	-0·68	0·09	1·08	-0·19	0·11	1·29	-0·29	0·02	0·81
Type of exercise	-0·25	0·01	1·00	0·07	0·04	0·89	-0·42	0·06	1·18	0·01	0·07	1·01	-0·38	0·10	1·09	-0·68	0·09	1·18	-0·60	0·08	1·06	-0·24	0·11	1·28	-0·29	0·02	0·75
Overall amount of exercise	-0·25	0·01	1·05	0·12	0·04	0·95	-0·44	0·07	1·24	0·01	0·07	0·99	-0·55	0·08	1·09	-0·72	0·10	1·21	-0·59	0·08	1·05	-0·26	0·11	1·27	-0·32	0·02	0·80
Weight	0·19	0·01	0·88	0·71	0·03	0·70	0·27	0·06	1·01	-0·34	0·07	1·00	0·32	0·08	0·85	0·58	0·08	0·99	0·04	0·04	0·86	0·27	0·07	0·98	0·26	0·02	0·62
Appetite	0·09	0·01	0·79	0·36	0·03	0·60	0·31	0·05	0·89	0·12	0·06	0·88	0·01	0·08	0·81	0·55	0·06	0·90	-0·14	0·04	0·82	0·24	0·06	0·89	-0·01	0·01	0·53
Physical health	-0·11	0·01	0·78	-0·66	0·03	0·65	-0·21	0·05	0·98	-0·01	0·07	0·98	-0·35	0·07	0·83	-0·26	0·07	1·00	-0·37	0·04	0·79	-0·10	0·07	0·93	-0·07	0·01	0·52
Sleep quality	-0·18	0·01	0·91	-0·25	0·04	0·86	-0·51	0·05	0·98	0·01	0·07	0·94	-0·43	0·08	0·95	-0·34	0·09	1·16	-0·53	0·07	0·98	-0·43	0·08	1·09	-0·14	0·02	0·65
Quality of life	-0·33	0·01	0·95	-0·82	0·03	0·69	-0·45	0·06	1·04	-0·23	0·06	0·87	-0·84	0·09	1·04	-0·27	0·08	1·10	-0·51	0·08	0·97	-0·24	0·12	1·12	-0·25	0·02	0·66
Mental burden	0·37	0·01	1·05	0·98	0·03	0·74	0·93	0·06	1·09	0·19	0·07	0·97	0·71	0·13	1·12	1·09	0·08	0·99	0·19	0·06	1·11	1·03	0·09	1·06	0·39	0·02	0·73
Emotional distress	0·32	0·01	1·02	0·96	0·04	0·87	1·08	0·05	0·95	0·39	0·08	1·13	0·62	0·13	1·07	1·10	0·07	0·90	-0·14	0·06	1·09	1·10	0·07	0·94	0·32	0·02	0·69
Family disputes	0·06	0·01	0·85	0·90	0·03	0·75	0·25	0·04	0·77	0·45	0·07	1·07	0·21	0·07	0·74	0·16	0·10	1·03	-0·22	0·07	1·04	0·34	0·08	0·92	0·12	0·01	0·52
Social support provided	0·08	0·01	0·87	-0·41	0·05	1·13	0·13	0·05	0·88	0·17	0·08	1·05	-0·50	0·09	1·01	0·40	0·09	1·02	0·22	0·10	1·03	0·45	0·09	1·03	0·02	0·01	0·40
Social support received	-0·05	0·01	0·84	-0·10	0·04	1·01	-0·04	0·04	0·81	0·14	0·07	1·01	-0·64	0·09	0·96	-0·02	0·06	0·88	-0·28	0·09	1·00	0·11	0·12	1·02	0·08	0·01	0·43
Social activities	-0·68	0·01	1·06	-0·91	0·03	0·75	-1·32	0·05	0·97	-0·14	0·07	0·98	-1·44	0·07	0·89	-1·33	0·07	1·03	-0·54	0·08	1·07	-1·35	0·08	1·02	-0·81	0·02	0·94
Working hours	-0·07	0·01	1·05	0·09	0·06	1·31	0·53	0·06	1·14	0·04	0·07	0·96	-0·09	0·08	0·98	0·52	0·13	1·37	-0·28	0·07	1·02	0·42	0·15	1·33	-0·33	0·02	0·76
Income	-0·36	0·01	0·92	-0·89	0·04	0·92	-0·33	0·05	0·94	-0·23	0·07	1·01	-0·25	0·08	0·93	-0·25	0·07	0·94	-0·45	0·07	0·95	-0·36	0·09	1·03	-0·44	0·02	0·76

Economic burden	0·24	0·01	1·01	0·77	0·04	0·95	0·53	0·05	0·90	0·02	0·08	1·11	0·19	0·08	0·91	0·16	0·12	1·14	0·02	0·07	1·10	0·40	0·08	1·00	0·29	0·02	0·75
Preference of future preparation																											
Online consultation with doctors	3·19	0·01	1·07	3·82	0·04	0·89	3·54	0·06	1·03	3·03	0·07	1·00	2·90	0·12	1·41	3·23	0·11	1·18	2·93	0·07	1·05	3·29	0·14	1·20	3·04	0·02	1·00
Instant personalized health by online chatbot	2·90	0·01	1·11	2·16	0·05	1·01	3·09	0·06	1·15	2·88	0·08	1·10	1·91	0·08	1·09	3·01	0·11	1·23	2·64	0·07	1·03	3·19	0·10	1·16	2·82	0·02	1·01
Telephone health advice	3·00	0·01	1·09	3·01	0·04	0·95	3·06	0·06	1·11	3·18	0·07	1·01	2·75	0·16	1·37	2·90	0·11	1·23	2·87	0·11	1·12	3·06	0·10	1·22	3·00	0·03	1·08
Online courses	3·17	0·01	1·12	3·25	0·06	1·30	3·59	0·05	0·94	3·32	0·07	0·98	2·68	0·16	1·41	3·16	0·11	1·18	2·91	0·07	1·08	3·67	0·08	1·01	2·98	0·03	1·07
Instant streaming courses	3·16	0·01	1·13	3·43	0·06	1·34	3·63	0·06	0·97	3·02	0·08	1·07	2·75	0·17	1·44	3·18	0·11	1·14	2·91	0·08	1·11	3·55	0·08	0·99	3·01	0·03	1·08
Receiving health information through email	2·84	0·01	1·11	2·15	0·05	1·10	2·89	0·05	1·01	3·33	0·07	0·98	2·39	0·13	1·25	2·66	0·10	1·19	2·57	0·06	1·02	3·01	0·10	1·14	2·72	0·03	1·07
Receiving health information through text messaging	3·00	0·01	1·12	2·93	0·05	1·16	3·00	0·05	1·03	3·10	0·08	1·11	2·13	0·13	1·20	2·51	0·09	1·18	2·62	0·08	1·09	2·90	0·11	1·23	2·92	0·03	1·02
Receiving health information from social media	2·92	0·01	1·16	2·69	0·06	1·25	3·01	0·06	1·11	3·01	0·09	1·16	1·88	0·10	1·11	2·60	0·10	1·20	2·84	0·09	1·13	3·00	0·08	1·14	2·92	0·03	1·03
Receiving health information from mobile app	2·96	0·02	1·12	2·60	0·06	1·21	3·02	0·05	1·01	3·18	0·07	1·03	1·99	0·11	1·18	2·62	0·10	1·20	3·06	0·60	1·13	2·90	0·10	1·12	2·89	0·03	1·05
Get medicine prescribed in a hospital visit/follow-up in a community pharmacy	3·37	0·01	1·07	3·35	0·05	1·15	3·60	0·05	0·96	3·01	0·07	1·04	3·05	0·15	1·37	3·62	0·10	1·12	3·34	0·08	1·07	3·60	0·12	1·19	3·45	0·03	1·10
Medicine delivery	3·50	0·01	1·11	4·10	0·04	0·91	3·88	0·05	0·94	3·23	0·07	0·97	2·81	0·16	1·48	3·84	0·10	1·11	3·44	0·07	1·12	3·99	0·09	1·03	3·62	0·03	1·04
Online shopping	3·33	0·02	1·16	3·99	0·04	0·90	3·86	0·05	0·96	3·32	0·06	0·91	2·90	0·17	1·50	3·48	0·11	1·24	2·79	0·09	1·19	3·56	0·11	1·19	3·30	0·03	1·12
Food delivery	3·27	0·02	1·18	3·21	0·13	1·27	3·81	0·05	0·93	3·01	0·06	0·94	2·68	0·18	1·57	3·25	0·12	1·28	2·86	0·09	1·23	3·54	0·11	1·18	3·41	0·03	1·08

(Continued)

Impact and preparation	India			Indonesia			Italy			Lebanon			Libya			Macau			Mainland China			Malaysia			Mexico		
	Mean	SE	SD	Mean	SE	SD	Mean	SE	SD	Mean	SE	SD	Mean	SE	SD	Mean	SE	SD	Mean	SE	SD	Mean	SE	SD	Mean	SE	SD
Perception of COVID-19' impact																											
Food types in daily meals	0·04	0·04	0·73	-0·21	0·17	1·01	0·15	0·05	0·61	-0·29	0·10	0·89	0·00	0·04	0·85	0·04	0·10	0·64	-0·11	0·07	0·82	-0·07	0·06	0·79	0·11	0·05	0·99
Consumption of fruits and vegetables	0·38	0·04	0·79	-0·13	0·13	0·93	0·27	0·06	0·69	-0·13	0·08	0·89	0·40	0·05	0·94	0·37	0·14	0·71	0·06	0·07	0·86	0·10	0·06	0·82	0·35	0·07	0·98
Consumption of frozen food/food products	-0·34	0·05	0·95	-0·32	0·12	0·87	-0·13	0·08	0·87	-0·54	0·08	0·86	-0·40	0·05	0·95	0·08	0·11	0·78	-0·39	0·09	0·95	-0·01	0·06	0·84	-0·14	0·05	1·00
Consumption of snacks	-0·29	0·05	0·98	-0·16	0·16	0·99	-0·20	0·09	1·01	-0·44	0·09	0·96	-0·44	0·06	1·12	0·02	0·11	0·87	-0·21	0·09	0·92	-0·05	0·06	0·85	-0·31	0·07	1·20
Soft drinks/juices/other sugary drinks	-0·58	0·06	1·04	-0·25	0·17	1·04	-0·24	0·08	0·91	-0·74	0·09	1·00	-0·63	0·05	1·06	-0·18	0·07	0·69	-0·32	0·12	1·02	-0·28	0·08	1·00	-0·54	0·06	1·15
Having a meal at home	0·68	0·06	0·97	0·26	0·20	1·07	0·61	0·07	0·88	0·46	0·17	1·10	0·57	0·05	1·03	0·85	0·12	0·89	1·04	0·12	1·10	0·57	0·07	0·94	1·16	0·05	1·02
Cooking at home	0·75	0·06	1·00	0·17	0·20	1·08	0·73	0·08	0·91	0·32	0·10	0·97	0·58	0·05	1·04	0·82	0·12	0·86	0·97	0·12	1·09	0·55	0·06	0·94	0·99	0·06	1·07
Eating takeout food	-0·62	0·06	1·06	-0·17	0·18	1·09	-0·09	0·10	1·06	-0·87	0·12	1·02	-0·78	0·07	1·11	0·30	0·17	1·04	-0·84	0·11	1·08	-0·01	0·06	0·99	-0·07	0·07	1·29
Taking TCM or natural health products	-0·10	0·06	0·99	-0·25	0·14	0·86	0·04	0·04	0·53	-0·34	0·07	0·97	0·08	0·05	1·15	-0·03	0·10	0·66	-0·18	0·08	0·77	-0·08	0·06	0·93	0·19	0·04	0·96
Taking oral supplements/vitamins	0·13	0·05	0·91	-0·06	0·17	1·04	0·17	0·08	0·69	-0·47	0·08	1·03	-0·06	0·06	1·22	0·15	0·08	0·60	-0·14	0·11	0·84	-0·03	0·05	0·85	0·42	0·04	0·94
Smoking tobacco	-0·43	0·05	0·89	-0·24	0·17	0·94	-0·25	0·07	0·76	-0·55	0·16	1·16	-0·60	0·06	1·09	-0·12	0·06	0·59	-0·37	0·12	0·90	-0·32	0·06	0·87	-0·60	0·05	1·05
Alcohol consumption	-0·41	0·04	0·83	-0·03	0·19	1·04	-0·14	0·07	0·78	-0·90	0·14	1·02	-0·76	0·06	0·98	-0·16	0·07	0·66	-0·39	0·08	0·79	-0·29	0·05	0·83	-0·43	0·04	1·00
Duration of sitting	0·42	0·05	0·91	-0·05	0·13	0·93	0·85	0·06	0·81	0·39	0·10	1·03	0·50	0·08	1·05	0·51	0·14	0·87	0·61	0·08	0·95	0·45	0·07	0·90	0·92	0·06	1·06

Duration of screen time	0·45	0·05	0·88	0·06	0·12	0·91	0·94	0·06	0·75	0·25	0·07	1·03	0·40	0·05	1·08	0·70	0·12	0·79	0·92	0·09	0·96	0·51	0·07	0·97	1·01	0·05	1·03
Frequency of exercise	-0·02	0·04	0·89	-0·19	0·12	0·89	-0·37	0·09	1·09	-0·36	0·09	1·02	-0·22	0·07	1·09	-0·22	0·17	0·98	-0·28	0·10	1·06	0·00	0·06	0·96	-0·24	0·09	1·31
Duration of exercise	0·00	0·04	0·91	-0·21	0·12	0·84	-0·45	0·08	1·01	-0·42	0·09	1·01	-0·31	0·06	1·09	-0·27	0·16	0·93	-0·27	0·09	1·03	-0·02	0·07	0·98	-0·31	0·09	1·30
Type of exercise	-0·03	0·05	0·84	-0·22	0·11	0·81	-0·35	0·08	1·04	-0·38	0·09	0·99	-0·32	0·05	1·03	-0·30	0·15	0·88	-0·36	0·09	1·03	-0·02	0·06	0·93	-0·28	0·09	1·29
Overall amount of exercise	-0·06	0·05	0·92	-0·24	0·13	0·89	-0·37	0·09	1·13	-0·38	0·09	0·99	-0·29	0·05	1·05	-0·24	0·16	0·95	-0·31	0·10	1·05	-0·01	0·06	0·96	-0·34	0·09	1·32
Weight	0·16	0·04	0·75	-0·27	0·12	0·87	0·22	0·07	0·82	-0·12	0·11	0·94	0·14	0·04	0·92	0·31	0·09	0·79	0·31	0·07	0·77	0·10	0·06	0·87	0·16	0·08	1·12
Appetite	0·08	0·03	0·66	-0·18	0·11	0·80	0·14	0·05	0·67	-0·30	0·17	1·10	0·04	0·04	0·96	0·11	0·08	0·56	-0·11	0·07	0·69	0·19	0·05	0·77	0·23	0·04	0·90
Physical health	0·10	0·03	0·61	-0·23	0·13	0·87	-0·19	0·04	0·59	-0·49	0·09	0·86	-0·10	0·04	0·80	-0·14	0·08	0·49	-0·16	0·06	0·62	0·03	0·05	0·75	-0·06	0·06	0·95
Sleep quality	0·13	0·04	0·83	-0·11	0·09	0·81	-0·22	0·10	0·94	-0·57	0·14	1·02	-0·18	0·05	1·02	-0·19	0·08	0·61	-0·09	0·09	0·78	0·13	0·07	0·93	-0·40	0·06	1·11
Quality of life	0·02	0·04	0·80	-0·25	0·11	0·85	-0·58	0·08	0·93	-0·96	0·11	0·95	-0·48	0·05	0·97	-0·22	0·07	0·56	-0·19	0·06	0·69	0·10	0·07	0·90	-0·28	0·05	1·02
Mental burden	0·33	0·04	0·82	-0·19	0·12	0·87	0·63	0·08	0·95	0·30	0·20	1·32	0·27	0·05	1·12	0·54	0·13	0·74	0·40	0·14	0·97	0·21	0·08	0·93	0·92	0·05	1·01
Emotional distress	0·30	0·04	0·83	-0·05	0·16	0·95	0·66	0·06	0·77	0·08	0·12	1·13	0·10	0·06	1·07	0·36	0·07	0·58	0·38	0·14	0·97	0·16	0·06	0·87	1·02	0·05	0·97
Family disputes	0·15	0·04	0·78	-0·23	0·11	0·69	0·16	0·06	0·72	0·12	0·14	1·07	**0·10**	0·04	0·98	0·15	0·07	0·43	0·12	0·13	0·87	0·02	0·04	0·65	0·23	0·04	0·97
Social support provided	0·05	0·04	0·73	-0·09	0·13	0·76	0·07	0·06	0·81	0·25	0·13	1·01	0·33	0·05	1·03	0·00	0·03	0·39	-0·08	0·12	0·81	0·09	0·05	0·78	0·29	0·07	1·03
Social support received	-0·07	0·04	0·74	-0·05	0·13	0·78	-0·01	0·05	0·60	-0·42	0·16	1·07	-0·06	0·04	0·94	0·05	0·03	0·37	-0·12	0·12	0·87	0·08	0·06	0·79	-0·20	0·05	0·93
Social activities	-0·33	0·06	0·95	0·12	0·22	1·16	-1·17	0·09	1·04	-0·66	0·16	1·13	-0·54	0·05	1·04	-0·66	0·13	0·82	-0·79	0·10	0·93	-0·05	0·07	0·98	-1·18	0·07	1·12
Working hours	0·06	0·04	0·89	-0·17	0·17	1·01	0·18	0·08	0·97	-0·52	0·09	1·08	-0·44	0·08	1·17	-0·11	0·10	0·76	-0·18	0·16	1·18	0·09	0·05	0·83	0·30	0·08	1·33
Income	-0·46	0·04	0·79	-0·09	0·14	0·96	-0·25	0·06	0·72	-0·88	0·06	0·89	-0·48	0·07	0·98	-0·22	0·08	0·65	-0·53	0·09	0·88	-0·01	0·05	0·74	-0·46	0·05	0·99

Economic burden	0·27	0·05	0·86	0·19	0·16	0·96	0·05	0·07	0·77	0·23	0·11	1·29	0·15	0·06	1·16	0·22	0·05	0·58	0·38	0·15	1·08	0·17	0·06	0·83	0·58	0·06	0·98
Preference of future preparation																											
Online consultation with doctors	2·51	0·07	1·12	2·89	0·11	0·75	3·30	0·09	1·12	2·94	0·06	0·88	3·26	0·06	1·15	3·26	0·07	0·81	3·23	0·11	1·04	3·13	0·07	0·99	3·41	0·06	1·12
Instant personalized health by online chatbot	2·32	0·07	1·07	2·75	0·08	0·63	2·93	0·10	1·24	2·62	0·09	0·95	2·98	0·05	1·14	3·07	0·06	0·77	3·41	0·12	0·99	3·05	0·07	0·99	3·28	0·05	1·10
Telephone health advice	2·47	0·07	1·05	2·80	0·08	0·63	3·21	0·09	1·08	2·78	0·08	0·90	2·97	0·06	1·12	2·97	0·06	0·76	3·29	0·13	1·07	3·01	0·07	0·99	3·08	0·05	1·12
Online courses	2·58	0·07	1·14	2·60	0·09	0·71	2·92	0·10	1·15	2·77	0·11	1·08	3·10	0·07	1·25	3·17	0·06	0·78	3·35	0·11	0·98	3·03	0·08	1·01	3·83	0·05	0·95
Instant streaming courses	2·54	0·07	1·14	2·73	0·08	0·66	2·97	0·09	1·12	2·85	0·09	1·02	2·94	0·07	1·25	3·16	0·06	0·79	3·30	0·11	0·97	3·17	0·07	0·98	3·78	0·05	0·99
Receiving health information through email	2·36	0·07	1·09	2·76	0·09	0·70	2·94	0·09	1·13	2·54	0·14	1·04	2·94	0·06	1·19	2·87	0·07	0·82	3·12	0·08	0·87	3·03	0·07	0·97	3·33	0·05	1·07
Receiving health information through text messaging	2·50	0·07	1·08	2·82	0·10	0·76	2·82	0·10	1·21	2·65	0·08	0·96	2·95	0·06	1·22	3·03	0·07	0·83	3·06	0·10	0·97	3·39	0·09	1·03	3·20	0·06	1·19
Receiving health information from social media	2·36	0·07	1·10	2·81	0·09	0·68	2·23	0·11	1·26	2·60	0·14	1·14	3·28	0·06	1·20	3·07	0·08	0·84	3·10	0·10	1·00	3·38	0·09	1·05	3·17	0·06	1·18
Receiving health information from mobile app	2·19	0·10	1·00	2·86	0·09	0·70	2·54	0·11	1·24	2·00	0·00	0·00	0·00	0·00	0·00	3·05	0·07	0·80	3·17	0·10	0·95	3·30	0·09	1·00	3·20	0·06	1·12
Get medicine prescribed in a hospital visit/follow-up in a community pharmacy	2·70	0·06	1·06	3·19	0·11	0·71	3·74	0·08	0·93	3·26	0·11	0·98	3·49	0·06	1·13	3·12	0·06	0·77	3·29	0·09	0·89	3·47	0·08	0·96	3·68	0·06	1·07
Medicine delivery	3·17	0·06	0·95	3·04	0·12	0·73	3·57	0·08	1·02	3·49	0·09	1·04	3·48	0·06	1·22	3·28	0·09	0·86	3·45	0·12	1·00	3·41	0·08	0·95	3·88	0·06	1·03
Online shopping	2·71	0·07	1·08	2·88	0·12	0·77	3·33	0·09	1·09	2·77	0·11	1·17	3·37	0·06	1·25	3·25	0·12	1·03	3·29	0·10	1·12	3·18	0·09	1·12	3·39	0·06	1·13
Food delivery	2·61	0·07	1·14	3·05	0·15	0·86	3·35	0·09	1·07	3·14	0·11	1·14	3·04	0·10	1·39	3·25	0·11	1·01	3·31	0·11	1·10	3·35	0·08	1·07	3·34	0·06	1·14

(Continued)

Impact and preparation	Nigeria			Philippines			Republic Of Sudan			Rwanda			Saudi Arabia			Singapore			South Africa			South Korea			Spain		
	Mean	SE	SD	Mean	SE	SD	Mean	SE	SD	Mean	SE	SD	Mean	SE	SD	Mean	SE	SD	Mean	SE	SD	Mean	SE	SD	Mean	SE	SD
Perception of COVID-19' impact																											
Food types in daily meals	-0·15	0·06	1·01	0·19	0·08	1·14	-0·08	0·10	0·96	-0·45	0·11	1·00	0·02	0·05	0·96	0·04	0·11	0·77	0·07	0·09	0·88	-0·16	0·02	0·76	-0·05	0·12	0·63
Consumption of fruits and vegetables	0·33	0·07	1·14	0·27	0·07	1·10	0·40	0·08	0·95	-0·29	0·14	1·24	0·26	0·04	0·92	0·19	0·07	0·67	0·24	0·09	0·88	0·05	0·02	0·77	0·58	0·14	0·74
Consumption of frozen food/food products	-0·42	0·07	1·03	0·27	0·07	1·11	-0·44	0·08	0·94	-0·52	0·12	1·18	-0·07	0·05	0·98	0·24	0·08	0·77	0·18	0·07	0·71	0·50	0·02	0·75	-0·02	0·09	0·51
Consumption of snacks	-0·36	0·07	1·05	-0·04	0·08	1·17	-0·44	0·08	1·02	-0·46	0·12	1·12	-0·23	0·06	1·12	0·09	0·08	0·84	0·48	0·11	1·04	0·14	0·02	0·80	0·01	0·17	0·94
Soft drinks/juices/other sugary drinks	-0·38	0·08	1·13	-0·24	0·08	1·24	-0·64	0·09	1·10	-0·69	0·12	1·12	-0·59	0·06	1·24	-0·34	0·12	0·96	0·02	0·12	1·17	-0·01	0·02	0·82	-0·03	0·16	0·75
Having a meal at home	0·56	0·07	1·09	0·75	0·07	1·11	0·63	0·10	1·17	0·62	0·14	1·19	1·02	0·06	1·11	0·90	0·11	0·93	1·01	0·12	1·05	1·12	0·02	0·77	0·80	0·18	0·88
Cooking at home	0·50	0·09	1·19	0·79	0·07	1·09	0·68	0·11	1·21	0·52	0·12	1·13	1·04	0·06	1·09	0·74	0·11	0·94	1·05	0·11	1·00	0·97	0·02	0·79	0·69	0·19	0·93
Eating takeout food	-0·41	0·09	1·18	-0·25	0·09	1·36	-0·91	0·08	1·02	-0·55	0·15	1·20	-0·60	0·06	1·24	0·10	0·11	1·13	-0·68	0·12	1·20	0·64	0·02	0·91	-0·17	0·20	0·95
Taking TCM or natural health products	-0·38	0·07	1·12	-0·16	0·07	1·04	0·24	0·10	1·18	-0·11	0·12	1·05	-0·20	0·06	1·18	-0·24	0·14	0·94	0·28	0·12	1·02	0·01	0·02	0·66	0·00	0·14	0·69
Taking oral supplements/vitamins	0·02	0·08	1·10	0·24	0·07	1·13	-0·04	0·11	1·16	-0·37	0·09	0·87	-0·21	0·06	1·14	0·20	0·09	0·74	0·60	0·11	0·99	0·27	0·02	0·66	-0·07	0·12	0·62
Smoking tobacco	-0·73	0·08	1·09	-0·65	0·08	1·23	-0·63	0·08	1·00	-0·55	0·11	0·92	-0·62	0·06	1·18	-0·29	0·10	0·72	-0·40	0·11	0·93	-0·40	0·02	0·88	-0·09	0·13	0·72
Alcohol consumption	-0·73	0·06	1·02	-0·63	0·08	1·25	-0·60	0·08	0·97	-0·48	0·12	1·03	-0·70	0·21	0·97	-0·30	0·10	0·81	-0·37	0·09	0·88	-0·47	0·02	0·90	-0·07	0·14	0·81
Duration of sitting	0·26	0·09	1·17	0·63	0·07	1·02	0·35	0·09	1·08	0·61	0·15	1·24	0·62	0·06	1·15	0·69	0·12	0·96	0·89	0·09	0·94	0·57	0·02	0·71	0·47	0·27	1·25

Duration of screen time	0·27	0·07	1·13	0·74	0·07	1·08	0·24	0·10	1·12	0·80	0·12	1·08	0·65	0·06	1·11	0·85	0·12	0·92	1·00	0·10	0·96	0·76	0·02	0·72	0·85	0·17	0·84
Frequency of exercise	0·14	0·07	1·03	-0·12	0·08	1·23	-0·27	0·09	1·08	0·34	0·12	1·13	-0·13	0·06	1·13	0·06	0·08	1·02	-0·13	0·11	1·13	-0·39	0·02	0·96	-0·06	0·27	1·22
Duration of exercise	0·13	0·07	1·05	-0·14	0·08	1·21	-0·37	0·09	1·07	0·26	0·12	1·16	-0·20	0·05	1·09	0·07	0·08	0·96	-0·26	0·11	1·14	-0·41	0·02	0·94	-0·17	0·30	1·34
Type of exercise	0·12	0·07	1·00	-0·18	0·08	1·12	-0·35	0·08	0·99	0·26	0·11	1·06	-0·21	0·05	1·08	0·01	0·06	0·86	-0·11	0·11	1·10	-0·44	0·02	0·88	-0·26	0·25	1·26
Overall amount of exercise	0·14	0·07	1·02	-0·09	0·08	1·20	-0·29	0·08	1·03	0·23	0·12	1·11	-0·20	0·05	1·10	0·02	0·07	1·00	-0·15	0·12	1·25	-0·45	0·02	0·96	-0·03	0·27	1·28
Weight	-0·03	0·06	0·94	0·24	0·07	1·08	0·00	0·09	0·95	0·27	0·11	0·99	0·10	0·05	0·98	0·04	0·12	0·92	0·31	0·11	1·03	0·23	0·02	0·73	0·46	0·13	0·70
Appetite	0·11	0·06	0·88	0·16	0·06	0·95	0·05	0·09	0·93	0·17	0·10	0·86	0·09	0·04	0·94	-0·09	0·08	0·68	0·28	0·09	0·93	0·06	0·02	0·67	0·20	0·18	0·70
Physical health	0·32	0·07	0·94	0·17	0·06	0·97	-0·17	0·07	0·77	-0·09	0·08	0·73	0·04	0·04	0·86	0·03	0·07	0·70	-0·07	0·08	0·80	-0·22	0·01	0·61	-0·17	0·14	0·64
Sleep quality	0·21	0·06	1·00	0·06	0·06	1·06	-0·10	0·09	1·01	-0·09	0·10	0·95	-0·05	0·05	1·01	-0·07	0·08	0·84	-0·11	0·11	1·03	-0·35	0·02	0·70	-0·34	0·14	0·73
Quality of life	0·14	0·09	1·10	0·08	0·06	1·04	-0·39	0·10	1·08	-0·46	0·09	0·81	-0·07	0·05	1·06	-0·17	0·09	0·84	-0·16	0·11	1·01	-0·77	0·02	0·79	-0·44	0·14	0·86
Mental burden	0·13	0·09	1·15	0·32	0·07	1·06	0·31	0·11	1·19	0·22	0·13	1·10	0·19	0·05	1·11	0·33	0·09	0·84	0·74	0·10	0·99	-0·11	0·03	1·09	0·88	0·15	0·77
Emotional distress	0·12	0·08	1·11	0·26	0·07	1·10	0·09	0·10	1·10	0·37	0·11	1·06	0·04	0·05	1·01	0·21	0·10	0·84	0·76	0·10	0·94	-0·13	0·02	0·96	0·73	0·13	0·75
Family disputes	-0·22	0·07	1·08	-0·30	0·06	1·00	-0·04	0·09	1·03	-0·24	0·12	0·95	-0·09	0·05	0·99	0·05	0·11	0·80	0·24	0·11	0·88	-0·08	0·01	0·54	0·36	0·15	0·75
Social support provided	-0·03	0·08	1·12	0·07	0·06	1·02	0·27	0·10	1·13	0·29	0·14	1·17	0·33	0·05	0·96	0·06	0·07	0·65	0·02	0·11	0·86	0·02	0·01	0·61	0·68	0·13	0·72
Social support received	-0·24	0·07	1·10	0·04	0·06	0·98	-0·12	0·10	1·04	-0·19	0·13	1·09	-0·11	0·04	0·90	0·05	0·07	0·63	-0·13	0·10	0·84	0·16	0·02	0·68	0·30	0·12	0·63
Social activities	-0·27	0·08	1·10	-0·44	0·07	1·14	-0·66	0·10	1·11	-0·95	0·14	1·20	-0·41	0·06	1·12	-0·82	0·07	0·77	-1·02	0·11	1·07	-0·68	0·02	0·78	-1·45	0·16	0·84
Working hours	-0·11	0·07	1·10	0·27	0·08	1·20	-0·66	0·09	1·06	-0·18	0·12	1·12	-0·29	0·06	1·12	0·37	0·12	0·95	0·01	0·12	1·21	-0·24	0·02	0·67	0·40	0·15	0·97
Income	-0·36	0·07	1·05	-0·22	0·07	1·13	-0·40	0·09	1·05	-0·71	0·12	1·02	-0·22	0·04	0·90	-0·16	0·11	0·79	-0·62	0·11	0·94	-0·34	0·02	0·86	-0·40	0·15	0·82

Economic burden	0·59	0·06	1·15	0·32	0·08	1·19	0·37	0·12	1·33	0·05	0·15	1·30	0·04	0·05	1·06	0·22	0·05	0·60	0·48	0·13	1·14	-0·11	0·02	0·95	-0·28	0·13	0·70
Preference of future preparation																											
Online consultation with doctors	3·31	0·07	1·10	3·55	0·06	1·00	3·34	0·10	1·12	3·31	0·14	1·18	3·26	0·06	1·17	2·81	0·18	1·13	3·33	0·12	1·12	3·20	0·02	0·92	2·90	0·23	1·04
Instant personalized health by online chatbot	3·15	0·07	1·11	3·09	0·07	1·05	3·13	0·12	1·22	3·81	0·13	1·13	2·96	0·06	1·21	2·40	0·15	1·09	2·73	0·15	1·26	2·98	0·02	0·96	2·74	0·26	1·12
Telephone health advice	3·41	0·07	1·06	3·29	0·07	1·04	3·25	0·12	1·20	3·82	0·13	1·17	3·20	0·07	1·21	2·52	0·15	1·04	3·09	0·13	1·17	2·79	0·02	0·95	2·74	0·23	1·03
Online courses	3·69	0·06	0·98	3·36	0·07	1·09	3·37	0·12	1·22	3·87	0·13	1·06	3·28	0·07	1·21	2·61	0·12	1·00	3·39	0·13	1·21	3·10	0·03	1·00	2·72	0·18	1·03
Instant streaming courses	3·54	0·07	1·04	3·38	0·07	1·09	3·21	0·11	1·21	3·77	0·15	1·19	3·17	0·06	1·22	2·76	0·19	1·21	3·41	0·14	1·28	3·03	0·03	0·98	3·04	0·21	1·08
Receiving health information through email	3·58	0·06	0·99	3·38	0·07	1·07	2·88	0·12	1·30	3·47	0·14	1·12	2·74	0·06	1·26	2·60	0·11	1·02	2·94	0·14	1·16	2·66	0·03	1·00	2·93	0·24	1·13
Receiving health information through text messaging	3·72	0·06	0·97	3·45	0·07	1·01	3·11	0·12	1·28	4·06	0·12	1·06	3·16	0·06	1·21	2·74	0·18	1·14	2·83	0·14	1·24	3·01	0·03	1·02	2·55	0·24	1·03
Receiving health information from social media	3·58	0·06	1·01	3·43	0·07	1·04	3·31	0·11	1·25	3·91	0·16	1·25	3·18	0·06	1·26	2·35	0·16	1·15	2·53	0·15	1·27	2·68	0·03	1·03	2·16	0·22	1·09
Receiving health information from mobile app	3·52	0·06	1·00	3·28	0·16	1·05	0·00	0·00	0·00	3·91	0·14	1·18	2·41	0·45	1·19	2·51	0·16	1·02	2·69	0·15	1·23	4·73	0·24	0·44	2·34	0·15	0·94
Get medicine prescribed in a hospital visit/follow-up in a community pharmacy	3·59	0·06	1·02	3·58	0·06	0·94	3·59	0·12	1·21	3·21	0·17	1·24	3·57	0·06	1·15	3·12	0·13	0·93	3·40	0·13	1·18	3·31	0·02	0·90	2·98	0·27	1·19
Medicine delivery	3·71	0·06	0·98	3·65	0·07	1·04	3·51	0·12	1·29	3·15	0·13	1·07	3·85	0·06	1·19	3·33	0·22	1·15	3·74	0·12	1·14	3·19	0·03	1·05	3·17	0·28	1·25
Online shopping	3·48	0·06	1·08	3·13	0·08	1·20	3·19	0·14	1·41	3·55	0·13	1·07	3·66	0·07	1·18	2·70	0·19	1·30	3·48	0·14	1·27	3·48	0·03	0·98	2·87	0·21	1·04
Food delivery	3·54	0·06	1·09	3·32	0·08	1·15	3·19	0·13	1·40	3·21	0·13	1·15	3·33	0·08	1·35	2·82	0·20	1·31	3·32	0·16	1·36	3·29	0·03	1·00	2·57	0·28	1·40

(Continued)

Impact and preparation	Thailand			United Kingdom			United States			Vietnam			EUR	AMR	EMR	WPR	AFR	SEAR	High income country	Upper middle income country	Lower middle income country	Low income country	High severity level	Medium severity level	Low severity level
	Mean	SE	SD	Mean	SE	SD	Mean	SE	SD	Mean	SE	SD	SE	SE	SE	SE	SE	SE	SE	SE	SE	SE	SE	SE	SE
Perception of COVID-19' impact																									
Food types in daily meals	-0·12	0·05	0·86	0·14	0·09	0·89	0·11	0·08	0·78	0·22	0·07	0·76	0·05	0·03	0·03	0·01	0·04	0·05	0·01	0·02	0·03	0·06	0·03	0·03	0·01
Consumption of fruits and vegetables	0·04	0·04	0·78	0·21	0·11	0·77	0·21	0·07	0·70	0·42	0·06	0·79	0·06	0·03	0·03	0·01	0·05	0·04	0·01	0·02	0·03	0·06	0·03	0·02	0·01
Consumption of frozen food/food products	-0·15	0·05	1·01	0·21	0·13	0·87	0·22	0·08	0·77	0·12	0·08	0·84	0·08	0·03	0·03	0·02	0·04	0·04	0·01	0·02	0·03	0·05	0·03	0·02	0·02
Consumption of snacks	-0·25	0·06	0·99	0·28	0·18	1·03	0·29	0·11	0·94	0·02	0·08	0·82	0·09	0·04	0·03	0·01	0·05	0·05	0·02	0·03	0·04	0·05	0·03	0·03	0·01
Soft drinks/juices/other sugary drinks	-0·25	0·06	1·04	-0·04	0·10	0·96	-0·07	0·10	0·79	-0·36	0·08	0·95	0·06	0·03	0·03	0·02	0·05	0·05	0·01	0·03	0·04	0·06	0·03	0·03	0·02
Having a meal at home	0·78	0·06	0·96	0·69	0·13	1·02	1·04	0·14	0·99	0·60	0·08	0·95	0·07	0·03	0·04	0·02	0·05	0·06	0·01	0·03	0·04	0·06	0·03	0·03	0·02
Cooking at home	0·52	0·05	1·01	0·41	0·28	1·29	1·00	0·13	0·99	0·57	0·08	0·99	0·14	0·03	0·04	0·02	0·05	0·06	0·02	0·03	0·04	0·06	0·04	0·03	0·02
Eating takeout food	0·47	0·06	1·07	-0·10	0·16	1·22	0·05	0·14	1·18	0·27	0·07	0·98	0·09	0·04	0·04	0·02	0·05	0·06	0·02	0·03	0·04	0·05	0·04	0·03	0·02
Taking TCM or natural health products	-0·63	0·05	0·99	-0·37	0·20	1·04	0·12	0·07	0·62	-0·22	0·06	0·82	0·11	0·02	0·04	0·01	0·04	0·05	0·01	0·02	0·03	0·06	0·03	0·03	0·01
Taking oral supplements/vitamins	-0·32	0·06	1·03	0·11	0·25	1·07	0·29	0·09	0·76	-0·29	0·11	0·97	0·12	0·03	0·04	0·02	0·05	0·05	0·01	0·03	0·04	0·06	0·03	0·03	0·02
Smoking tobacco	-0·78	0·05	1·00	-0·20	0·08	0·82	-0·08	0·10	0·72	-0·53	0·09	1·02	0·05	0·03	0·04	0·02	0·04	0·05	0·01	0·03	0·04	0·05	0·03	0·03	0·02
Alcohol consumption	-0·68	0·05	0·99	-0·28	0·13	1·02	0·32	0·12	0·89	-0·51	0·08	0·99	0·07	0·03	0·04	0·01	0·04	0·05	0·01	0·02	0·04	0·05	0·03	0·03	0·01

Duration of sitting	0·37	0·05	0·92	1·00	0·14	0·84	1·01	0·08	0·80	-0·02	0·07	0·93	0·07	0·03	0·04	0·01	0·05	0·05	0·01	0·03	0·03	0·06	0·03	0·03	0·01
Duration of screen time	0·57	0·04	0·87	1·12	0·14	0·79	1·03	0·07	0·76	0·20	0·07	0·88	0·07	0·03	0·03	0·02	0·05	0·04	0·01	0·03	0·03	0·06	0·03	0·03	0·01
Frequency of exercise	-0·09	0·05	0·94	-0·28	0·16	1·28	-0·29	0·13	1·15	0·35	0·06	0·82	0·09	0·04	0·04	0·02	0·04	0·04	0·02	0·03	0·03	0·06	0·04	0·03	0·02
Duration of exercise	-0·15	0·04	0·86	-0·26	0·15	1·25	-0·33	0·11	1·08	0·31	0·06	0·83	0·08	0·04	0·03	0·02	0·05	0·04	0·02	0·03	0·03	0·06	0·03	0·03	0·02
Type of exercise	-0·04	0·04	0·90	-0·27	0·14	1·20	-0·21	0·11	1·09	0·23	0·06	0·82	0·08	0·04	0·03	0·02	0·04	0·04	0·01	0·03	0·03	0·05	0·03	0·02	0·02
Overall amount of exercise	-0·12	0·05	0·90	-0·30	0·17	1·34	-0·16	0·16	1·28	0·27	0·06	0·78	0·09	0·04	0·03	0·02	0·04	0·04	0·02	0·03	0·03	0·05	0·04	0·03	0·02
Weight	0·20	0·04	0·74	0·37	0·13	1·02	0·07	0·13	0·96	0·11	0·06	0·74	0·07	0·04	0·03	0·01	0·04	0·04	0·01	0·03	0·03	0·06	0·03	0·03	0·01
Appetite	0·15	0·03	0·67	0·11	0·10	0·88	0·26	0·12	0·85	0·15	0·04	0·59	0·05	0·03	0·04	0·01	0·04	0·04	0·01	0·03	0·03	0·05	0·03	0·02	0·01
Physical health	0·18	0·04	0·67	-0·30	0·12	0·98	-0·04	0·10	0·80	0·22	0·04	0·58	0·06	0·03	0·03	0·01	0·04	0·04	0·01	0·02	0·03	0·04	0·03	0·02	0·01
Sleep quality	0·23	0·04	0·79	-0·45	0·12	0·98	-0·43	0·11	0·96	0·16	0·05	0·67	0·07	0·03	0·04	0·01	0·04	0·03	0·01	0·03	0·03	0·06	0·03	0·02	0·01
Quality of life	0·33	0·04	0·83	-0·58	0·14	1·14	-0·28	0·11	0·99	0·07	0·05	0·70	0·08	0·03	0·04	0·01	0·05	0·04	0·01	0·03	0·03	0·06	0·03	0·03	0·01
Mental burden	0·35	0·04	0·86	0·72	0·14	1·09	0·83	0·10	0·98	0·06	0·05	0·79	0·07	0·03	0·05	0·02	0·05	0·04	0·02	0·03	0·03	0·06	0·04	0·03	0·02
Emotional distress	0·19	0·05	0·90	0·55	0·12	0·99	0·71	0·12	0·99	-0·07	0·06	0·78	0·06	0·03	0·04	0·02	0·05	0·05	0·01	0·03	0·03	0·06	0·03	0·03	0·02
Family disputes	-0·23	0·04	0·84	0·05	0·07	0·70	0·13	0·09	0·78	-0·20	0·06	0·78	0·05	0·03	0·04	0·02	0·05	0·04	0·01	0·03	0·03	0·06	0·03	0·02	0·01
Social support provided	0·13	0·05	0·82	0·00	0·09	0·81	0·13	0·12	0·92	-0·15	0·06	0·76	0·05	0·04	0·04	0·01	0·05	0·04	0·01	0·03	0·03	0·06	0·03	0·02	0·01
Social support received	-0·14	0·04	0·82	-0·18	0·16	0·89	-0·08	0·10	0·85	-0·10	0·05	0·72	0·08	0·03	0·04	0·02	0·04	0·04	0·01	0·03	0·03	0·06	0·03	0·02	0·01
Social activities	-0·03	0·06	1·16	-1·06	0·18	1·14	-1·07	0·15	1·04	-0·08	0·05	0·74	0·09	0·03	0·04	0·02	0·05	0·06	0·01	0·03	0·04	0·06	0·04	0·03	0·02
Working hours	0·30	0·05	0·92	0·12	0·14	1·13	0·46	0·10	1·00	0·10	0·06	0·82	0·07	0·04	0·04	0·02	0·04	0·05	0·01	0·03	0·04	0·06	0·04	0·03	0·02

Income	0·21	0·04	0·84	-0·30	0·10	0·79	0·01	0·09	0·73	-0·34	0·07	0·87	0·05	0·03	0·03	0·02	0·04	0·04	0·01	0·02	0·03	0·06	0·03	0·02	0·01
Economic burden	0·29	0·05	0·94	0·02	0·11	0·83	0·04	0·07	0·68	-0·04	0·07	0·95	0·06	0·03	0·04	0·02	0·05	0·05	0·01	0·03	0·04	0·07	0·03	0·03	0·02
Preference of future preparation																									
Online consultation with doctors	3·07	0·06	1·13	3·40	0·11	1·05	3·45	0·14	1·12	3·11	0·13	0·94	0·07	0·04	0·03	0·02	0·04	0·05	0·02	0·03	0·04	0·06	0·03	0·03	0·02
Instant personalized health by online chatbot	2·89	0·06	1·16	2·61	0·17	1·31	2·49	0·13	1·17	3·03	0·12	0·97	0·10	0·04	0·04	0·02	0·05	0·04	0·02	0·03	0·03	0·07	0·03	0·03	0·02
Telephone health advice	2·93	0·07	1·19	3·24	0·20	1·27	3·00	0·13	1·15	3·05	0·13	0·96	0·10	0·04	0·04	0·02	0·04	0·04	0·02	0·03	0·04	0·07	0·03	0·03	0·02
Online courses	3·34	0·07	1·19	2·97	0·18	1·25	3·18	0·14	1·25	2·97	0·13	0·98	0·09	0·04	0·04	0·02	0·04	0·05	0·02	0·03	0·04	0·07	0·04	0·03	0·02
Instant streaming courses	3·47	0·07	1·23	2·96	0·15	1·21	3·38	0·14	1·22	3·11	0·09	0·87	0·08	0·04	0·04	0·02	0·05	0·05	0·02	0·03	0·03	0·07	0·04	0·03	0·02
Receiving health information through email	2·95	0·06	1·18	2·81	0·16	1·16	2·71	0·13	1·11	2·97	0·12	0·89	0·09	0·04	0·04	0·02	0·04	0·04	0·02	0·03	0·03	0·07	0·04	0·03	0·02
Receiving health information through text messaging	3·23	0·06	1·15	2·68	0·15	1·15	2·63	0·13	1·23	3·09	0·12	0·86	0·08	0·04	0·04	0·02	0·05	0·04	0·02	0·03	0·04	0·07	0·03	0·03	0·02
Receiving health information from social media	3·32	0·07	1·19	2·19	0·15	1·24	2·18	0·11	1·08	3·03	0·12	0·90	0·09	0·04	0·05	0·02	0·05	0·05	0·02	0·03	0·04	0·07	0·04	0·03	0·02
Receiving health information from mobile app	3·28	0·06	1·10	2·36	0·22	1·30	2·31	0·36	1·23	3·11	0·12	0·89	0·11	0·04	0·33	0·03	0·04	0·05	0·03	0·03	0·05	0·07	0·05	0·04	0·02
Get medicine prescribed in a hospital visit/follow-up in a community pharmacy	3·21	0·06	1·11	3·29	0·28	1·29	3·24	0·12	1·09	2·88	0·12	1·00	0·14	0·04	0·04	0·02	0·04	0·04	0·02	0·03	0·04	0·07	0·04	0·03	0·02
Medicine delivery	3·52	0·06	1·15	3·17	0·29	1·54	3·58	0·13	1·18	2·84	0·12	1·02	0·15	0·04	0·04	0·02	0·04	0·04	0·02	0·03	0·04	0·07	0·04	0·03	0·02
Online shopping	3·64	0·07	1·18	3·65	0·16	1·26	3·55	0·12	1·03	2·80	0·13	1·07	0·09	0·04	0·05	0·02	0·04	0·05	0·02	0·03	0·04	0·08	0·04	0·03	0·02
Food delivery	3·64	0·07	1·19	3·60	0·18	1·36	3·48	0·12	1·22	2·87	0·13	1·01	0·10	0·04	0·05	0·02	0·04	0·05	0·02	0·03	0·04	0·07	0·04	0·03	0·02

Figure S1. Visualization of the perceived impact of COVID-19 on lifestyles and health-related areas as well as perceived importance of possible preparations by country, World Health Organization (WHO) region, economic development level, and COVID-19 severity level.

a. Weighted mean of COVID-19's impact on lifestyles and health-related issues by country.

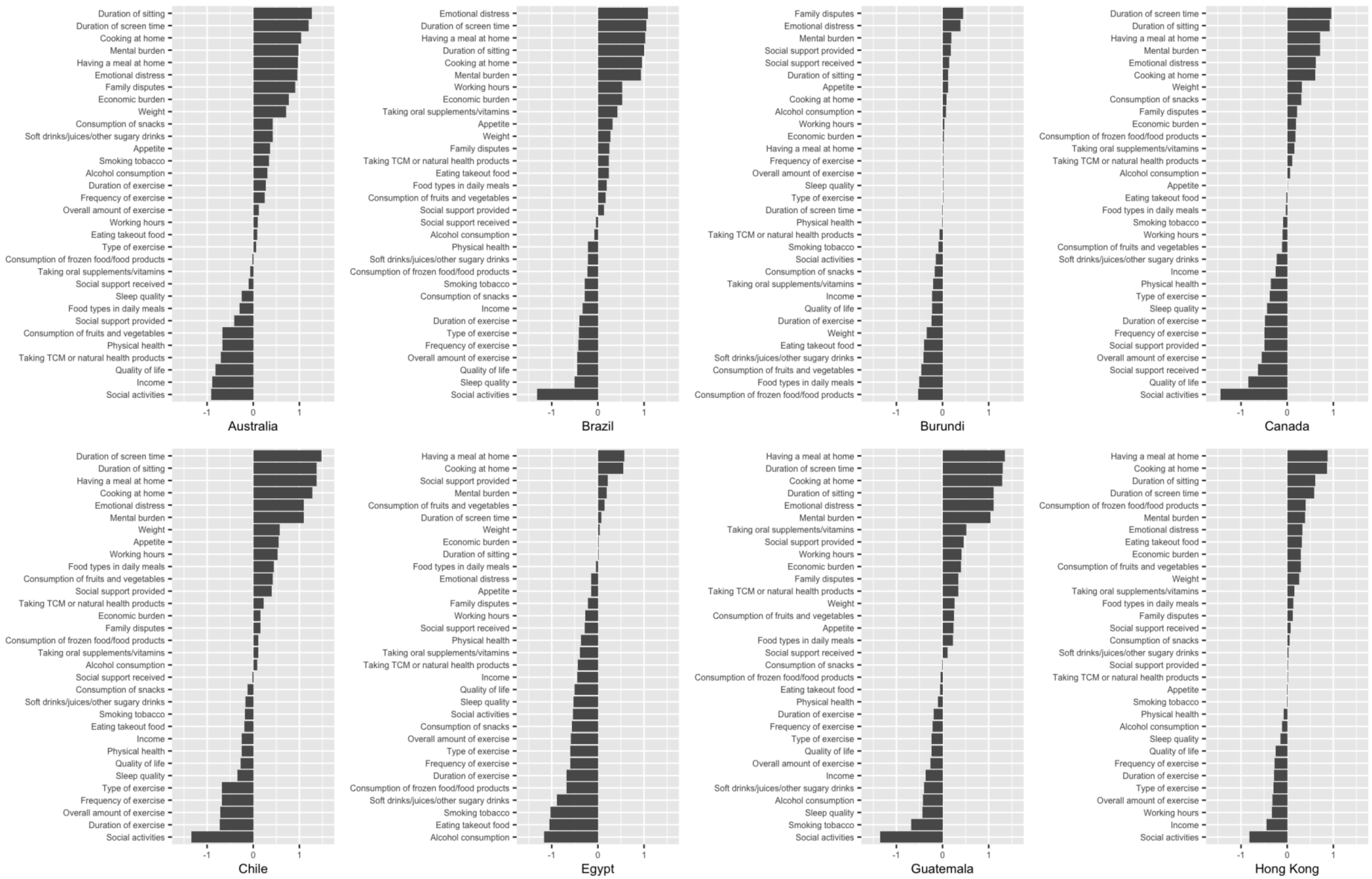
Duration of sitting
Duration of screen time
Cooking at home
Mental burden
Having a meal at home
Emotional distress
Family disputes
Economic burden
Weight
Consumption of snacks
Soft drinks/juices/other sugary drinks
Appetite
Smoking tobacco
Alcohol consumption
Duration of exercise
Frequency of exercise
Overall amount of exercise
Working hours
Eating takeout food
Type of exercise
Consumption of frozen food/food products
Taking oral supplements/vitamins
Social support received
Sleep quality
Food types in daily meals
Social support provided
Consumption of fruits and vegetables
Physical health
Taking TCM or natural health products
Quality of life
Income
Social activities
-1
0
1
Australia
Emotional distress
Duration of screen time
Having a meal at home
Duration of sitting
Cooking at home
Mental burden
Working hours
Economic burden
Taking oral supplements/vitamins
Appetite
Weight
Family disputes
Taking TCM or natural health products
Eating takeout food
Food types in daily meals
Consumption of fruits and vegetables
Social support provided
Social support received
Alcohol consumption
Physical health
Soft drinks/juices/other sugary drinks
Consumption of frozen food/food products
Smoking tobacco
Consumption of snacks
Income
Duration of exercise
Type of exercise
Frequency of exercise
Overall amount of exercise
Quality of life
Sleep quality
Social activities
-1
0
1
Brazil
Family disputes
Emotional distress
Mental burden
Social support provided
Social support received
Duration of sitting
Appetite
Cooking at home
Alcohol consumption
Working hours
Economic burden
Having a meal at home
Frequency of exercise
Overall amount of exercise
Sleep quality
Type of exercise
Duration of screen time
Physical health
Taking TCM or natural health products
Smoking tobacco
Social activities
Consumption of snacks
Taking oral supplements/vitamins
Income
Quality of life
Duration of exercise
Weight
Eating takeout food
Soft drinks/juices/other sugary drinks
Consumption of fruits and vegetables
Food types in daily meals
Consumption of frozen food/food products
-1
0
1
Burundi
Duration of screen time
Duration of sitting
Having a meal at home
Mental burden
Emotional distress
Cooking at home
Weight
Consumption of snacks
Family disputes
Economic burden
Consumption of frozen food/food products
Taking oral supplements/vitamins
Taking TCM or natural health products
Alcohol consumption
Appetite
Eating takeout food
Food types in daily meals
Smoking tobacco
Working hours
Consumption of fruits and vegetables
Soft drinks/juices/other sugary drinks
Income
Physical health
Type of exercise
Sleep quality
Duration of exercise
Frequency of exercise
Social support provided
Overall amount of exercise
Social support received
Quality of life
Social activities
-1
0
1
Canada
Duration of screen time
Duration of sitting
Having a meal at home
Cooking at home
Emotional distress
Mental burden
Weight
Appetite
Working hours
Food types in daily meals
Consumption of fruits and vegetables
Social support provided
Taking TCM or natural health products
Economic burden
Family disputes
Consumption of frozen food/food products
Taking oral supplements/vitamins
Alcohol consumption
Social support received
Consumption of snacks
Soft drinks/juices/other sugary drinks
Smoking tobacco
Eating takeout food
Income
Physical health
Quality of life
Sleep quality
Type of exercise
Frequency of exercise
Overall amount of exercise
Duration of exercise
Social activities
-1
0
1
Chile
Having a meal at home
Cooking at home
Social support provided
Mental burden
Consumption of fruits and vegetables
Duration of screen time
Weight
Economic burden
Duration of sitting
Food types in daily meals
Emotional distress
Appetite
Family disputes
Working hours
Social support received
Physical health
Taking oral supplements/vitamins
Taking TCM or natural health products
Income
Quality of life
Sleep quality
Social activities
Consumption of snacks
Overall amount of exercise
Type of exercise
Frequency of exercise
Duration of exercise
Consumption of frozen food/food products
Soft drinks/juices/other sugary drinks
Smoking tobacco
Eating takeout food
Alcohol consumption
-1
0
1
Egypt
Having a meal at home
Duration of screen time
Cooking at home
Duration of sitting
Emotional distress
Mental burden
Taking oral supplements/vitamins
Social support provided
Working hours
Economic burden
Family disputes
Taking TCM or natural health products
Weight
Consumption of fruits and vegetables
Appetite
Food types in daily meals
Social support received
Consumption of snacks
Consumption of frozen food/food products
Eating takeout food
Physical health
Duration of exercise
Frequency of exercise
Type of exercise
Quality of life
Overall amount of exercise
Income
Soft drinks/juices/other sugary drinks
Alcohol consumption
Sleep quality
Smoking tobacco
Social activities
-1
0
1
Guatemala
Having a meal at home
Cooking at home
Duration of sitting
Duration of screen time
Consumption of frozen food/food products
Mental burden
Emotional distress
Eating takeout food
Economic burden
Consumption of fruits and vegetables
Weight
Taking oral supplements/vitamins
Food types in daily meals
Family disputes
Social support received
Consumption of snacks
Soft drinks/juices/other sugary drinks
Social support provided
Taking TCM or natural health products
Appetite
Smoking tobacco
Physical health
Alcohol consumption
Sleep quality
Quality of life
Frequency of exercise
Duration of exercise
Type of exercise
Overall amount of exercise
Working hours
Income
Social activities
-1
0
1
Hong Kong

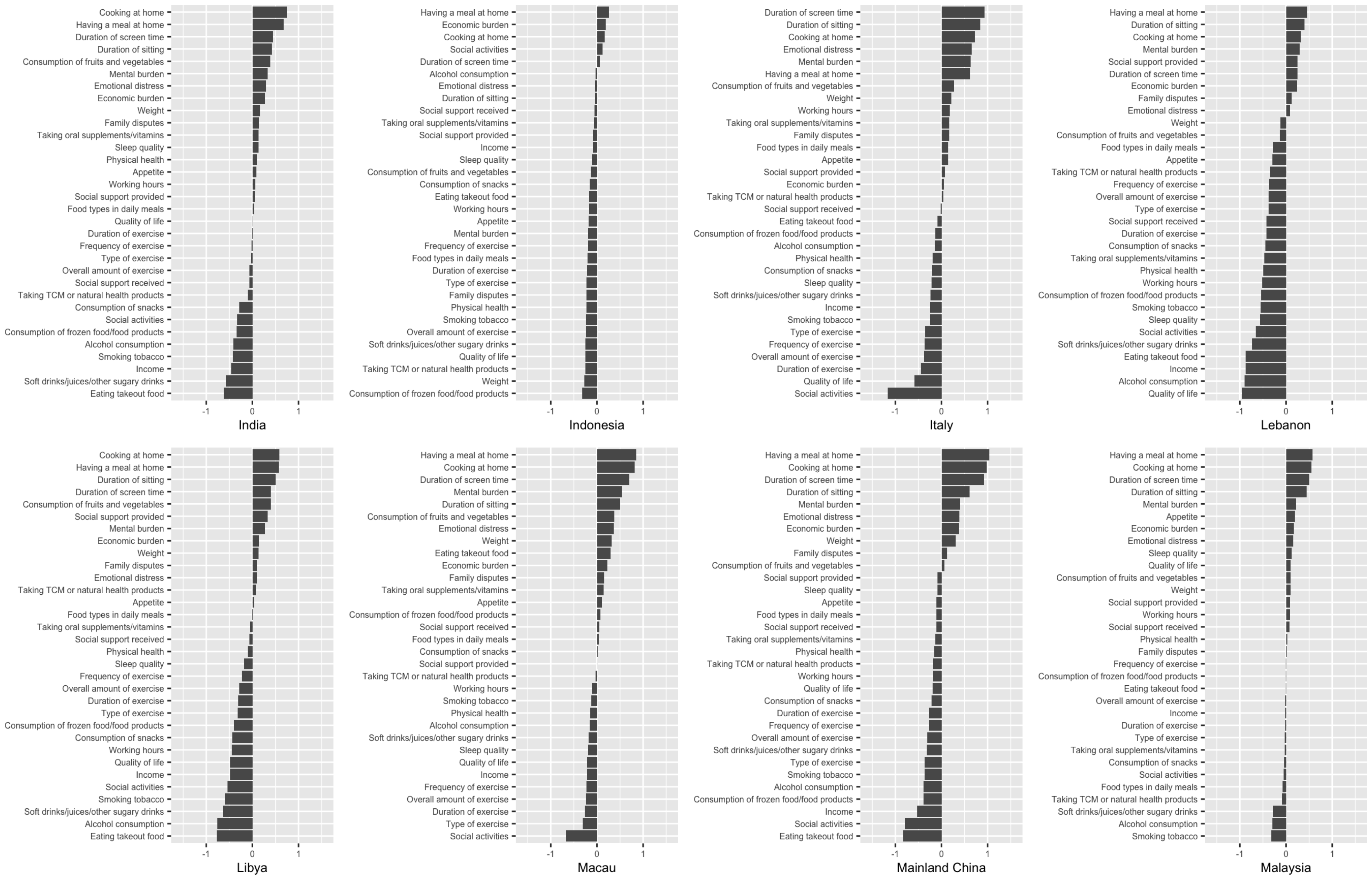
India
Cooking at home
Having a meal at home
Duration of screen time
Duration of sitting
Consumption of fruits and vegetables
Mental burden
Emotional distress
Economic burden
Weight
Family disputes
Taking oral supplements/vitamins
Sleep quality
Physical health
Appetite
Working hours
Social support provided
Food types in daily meals
Quality of life
Duration of exercise
Frequency of exercise
Type of exercise
Overall amount of exercise
Social support received
Taking TCM or natural health products
Consumption of snacks
Social activities
Consumption of frozen food/food products
Alcohol consumption
Smoking tobacco
Income
Soft drinks/juices/other sugary drinks
Eating takeout food
-1
0
1
Indonesia
Having a meal at home
Economic burden
Cooking at home
Social activities
Duration of screen time
Alcohol consumption
Emotional distress
Duration of sitting
Social support received
Taking oral supplements/vitamins
Social support provided
Income
Sleep quality
Consumption of fruits and vegetables
Consumption of snacks
Eating takeout food
Working hours
Appetite
Mental burden
Frequency of exercise
Food types in daily meals
Duration of exercise
Type of exercise
Family disputes
Physical health
Smoking tobacco
Overall amount of exercise
Soft drinks/juices/other sugary drinks
Quality of life
Taking TCM or natural health products
Weight
Consumption of frozen food/food products
-1
0
1
Italy
Duration of screen time
Duration of sitting
Cooking at home
Emotional distress
Mental burden
Having a meal at home
Consumption of fruits and vegetables
Weight
Working hours
Taking oral supplements/vitamins
Family disputes
Food types in daily meals
Appetite
Social support provided
Economic burden
Taking TCM or natural health products
Social support received
Eating takeout food
Consumption of frozen food/food products
Alcohol consumption
Physical health
Consumption of snacks
Sleep quality
Soft drinks/juices/other sugary drinks
Income
Smoking tobacco
Type of exercise
Frequency of exercise
Overall amount of exercise
Duration of exercise
Quality of life
Social activities
-1
0
1
Lebanon
Having a meal at home
Duration of sitting
Cooking at home
Mental burden
Social support provided
Duration of screen time
Economic burden
Family disputes
Emotional distress
Weight
Consumption of fruits and vegetables
Food types in daily meals
Appetite
Taking TCM or natural health products
Frequency of exercise
Overall amount of exercise
Type of exercise
Social support received
Duration of exercise
Consumption of snacks
Taking oral supplements/vitamins
Physical health
Working hours
Consumption of frozen food/food products
Smoking tobacco
Sleep quality
Social activities
Soft drinks/juices/other sugary drinks
Eating takeout food
Income
Alcohol consumption
Quality of life
-1
0
1
Libya
Cooking at home
Having a meal at home
Duration of sitting
Duration of screen time
Consumption of fruits and vegetables
Social support provided
Mental burden
Economic burden
Weight
Family disputes
Emotional distress
Taking TCM or natural health products
Appetite
Food types in daily meals
Taking oral supplements/vitamins
Social support received
Physical health
Sleep quality
Frequency of exercise
Overall amount of exercise
Duration of exercise
Type of exercise
Consumption of frozen food/food products
Consumption of snacks
Working hours
Quality of life
Income
Social activities
Smoking tobacco
Soft drinks/juices/other sugary drinks
Alcohol consumption
Eating takeout food
-1
0
1
Macau
Having a meal at home
Cooking at home
Duration of screen time
Mental burden
Duration of sitting
Consumption of fruits and vegetables
Emotional distress
Weight
Eating takeout food
Economic burden
Family disputes
Taking oral supplements/vitamins
Appetite
Consumption of frozen food/food products
Social support received
Food types in daily meals
Consumption of snacks
Social support provided
Taking TCM or natural health products
Working hours
Smoking tobacco
Physical health
Alcohol consumption
Soft drinks/juices/other sugary drinks
Sleep quality
Quality of life
Income
Frequency of exercise
Overall amount of exercise
Duration of exercise
Type of exercise
Social activities
-1
0
1
Mainland China
Having a meal at home
Cooking at home
Duration of screen time
Duration of sitting
Mental burden
Emotional distress
Economic burden
Weight
Family disputes
Consumption of fruits and vegetables
Social support provided
Sleep quality
Appetite
Food types in daily meals
Social support received
Taking oral supplements/vitamins
Physical health
Taking TCM or natural health products
Working hours
Quality of life
Consumption of snacks
Duration of exercise
Frequency of exercise
Overall amount of exercise
Soft drinks/juices/other sugary drinks
Type of exercise
Smoking tobacco
Alcohol consumption
Consumption of frozen food/food products
Income
Social activities
Eating takeout food
-1
0
1
Malaysia
Having a meal at home
Cooking at home
Duration of screen time
Duration of sitting
Mental burden
Appetite
Economic burden
Emotional distress
Sleep quality
Quality of life
Consumption of fruits and vegetables
Weight
Social support provided
Working hours
Social support received
Physical health
Family disputes
Frequency of exercise
Consumption of frozen food/food products
Eating takeout food
Overall amount of exercise
Income
Duration of exercise
Type of exercise
Taking oral supplements/vitamins
Consumption of snacks
Social activities
Food types in daily meals
Taking TCM or natural health products
Soft drinks/juices/other sugary drinks
Alcohol consumption
Smoking tobacco
-1
0
1

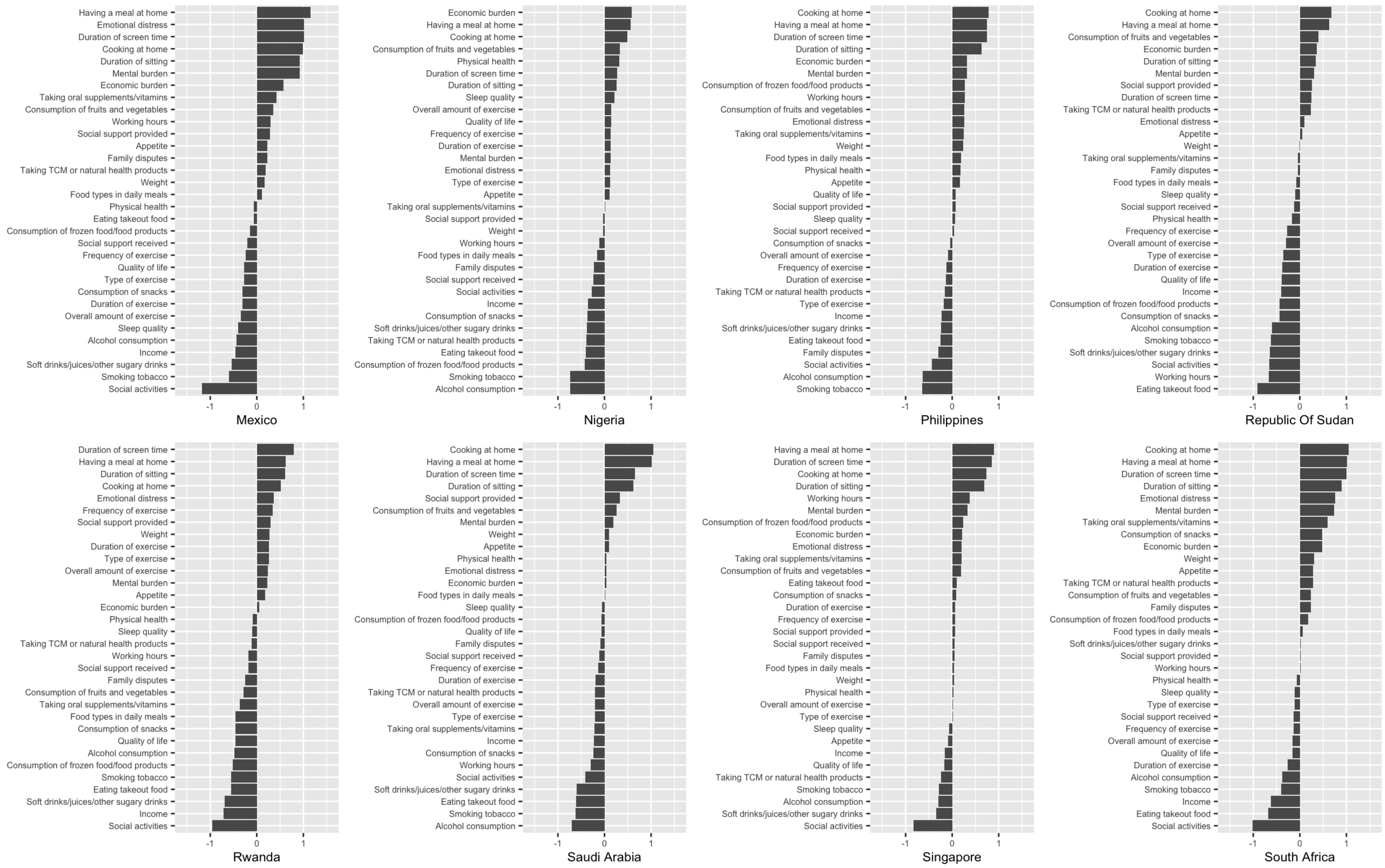
Having a meal at home
Emotional distress
Duration of screen time
Cooking at home
Duration of sitting
Mental burden
Economic burden
Taking oral supplements/vitamins
Consumption of fruits and vegetables
Working hours
Social support provided
Appetite
Family disputes
Taking TCM or natural health products
Weight
Food types in daily meals
Physical health
Eating takeout food
Consumption of frozen food/food products
Social support received
Frequency of exercise
Quality of life
Type of exercise
Consumption of snacks
Duration of exercise
Overall amount of exercise
Sleep quality
Alcohol consumption
Income
Soft drinks/juices/other sugary drinks
Smoking tobacco
Social activities
-1
0
1
Mexico
Economic burden
Having a meal at home
Cooking at home
Consumption of fruits and vegetables
Physical health
Duration of screen time
Duration of sitting
Sleep quality
Overall amount of exercise
Quality of life
Frequency of exercise
Duration of exercise
Mental burden
Emotional distress
Type of exercise
Appetite
Taking oral supplements/vitamins
Social support provided
Weight
Working hours
Food types in daily meals
Family disputes
Social support received
Social activities
Income
Consumption of snacks
Soft drinks/juices/other sugary drinks
Taking TCM or natural health products
Eating takeout food
Consumption of frozen food/food products
Smoking tobacco
Alcohol consumption
-1
0
1
Nigeria
Cooking at home
Having a meal at home
Duration of screen time
Duration of sitting
Economic burden
Mental burden
Consumption of frozen food/food products
Working hours
Consumption of fruits and vegetables
Emotional distress
Taking oral supplements/vitamins
Weight
Food types in daily meals
Physical health
Appetite
Quality of life
Social support provided
Sleep quality
Social support received
Consumption of snacks
Overall amount of exercise
Frequency of exercise
Duration of exercise
Taking TCM or natural health products
Type of exercise
Income
Soft drinks/juices/other sugary drinks
Eating takeout food
Family disputes
Social activities
Alcohol consumption
Smoking tobacco
-1
0
1
Philippines
Cooking at home
Having a meal at home
Consumption of fruits and vegetables
Economic burden
Duration of sitting
Mental burden
Social support provided
Duration of screen time
Taking TCM or natural health products
Emotional distress
Appetite
Weight
Taking oral supplements/vitamins
Family disputes
Food types in daily meals
Sleep quality
Social support received
Physical health
Frequency of exercise
Overall amount of exercise
Type of exercise
Duration of exercise
Quality of life
Income
Consumption of frozen food/food products
Consumption of snacks
Alcohol consumption
Smoking tobacco
Soft drinks/juices/other sugary drinks
Social activities
Working hours
Eating takeout food
-1
0
1
Republic Of Sudan
Duration of screen time
Having a meal at home
Duration of sitting
Cooking at home
Emotional distress
Frequency of exercise
Social support provided
Weight
Duration of exercise
Type of exercise
Overall amount of exercise
Mental burden
Appetite
Economic burden
Physical health
Sleep quality
Taking TCM or natural health products
Working hours
Social support received
Family disputes
Consumption of fruits and vegetables
Taking oral supplements/vitamins
Food types in daily meals
Consumption of snacks
Quality of life
Alcohol consumption
Consumption of frozen food/food products
Smoking tobacco
Eating takeout food
Soft drinks/juices/other sugary drinks
Income
Social activities
-1
0
1
Rwanda
Cooking at home
Having a meal at home
Duration of screen time
Duration of sitting
Social support provided
Consumption of fruits and vegetables
Mental burden
Weight
Appetite
Physical health
Emotional distress
Economic burden
Food types in daily meals
Sleep quality
Consumption of frozen food/food products
Quality of life
Family disputes
Social support received
Frequency of exercise
Duration of exercise
Taking TCM or natural health products
Overall amount of exercise
Type of exercise
Taking oral supplements/vitamins
Income
Consumption of snacks
Working hours
Social activities
Soft drinks/juices/other sugary drinks
Eating takeout food
Smoking tobacco
Alcohol consumption
-1
0
1
Saudi Arabia
Having a meal at home
Duration of screen time
Cooking at home
Duration of sitting
Working hours
Mental burden
Consumption of frozen food/food products
Economic burden
Emotional distress
Taking oral supplements/vitamins
Consumption of fruits and vegetables
Eating takeout food
Consumption of snacks
Duration of exercise
Frequency of exercise
Social support provided
Social support received
Family disputes
Food types in daily meals
Weight
Physical health
Overall amount of exercise
Type of exercise
Sleep quality
Appetite
Income
Quality of life
Taking TCM or natural health products
Smoking tobacco
Alcohol consumption
Soft drinks/juices/other sugary drinks
Social activities
-1
0
1
Singapore
Cooking at home
Having a meal at home
Duration of screen time
Duration of sitting
Emotional distress
Mental burden
Taking oral supplements/vitamins
Consumption of snacks
Economic burden
Weight
Appetite
Taking TCM or natural health products
Consumption of fruits and vegetables
Family disputes
Consumption of frozen food/food products
Food types in daily meals
Soft drinks/juices/other sugary drinks
Social support provided
Working hours
Physical health
Sleep quality
Type of exercise
Social support received
Frequency of exercise
Overall amount of exercise
Quality of life
Duration of exercise
Alcohol consumption
Smoking tobacco
Income
Eating takeout food
Social activities
-1
0
1
South Africa

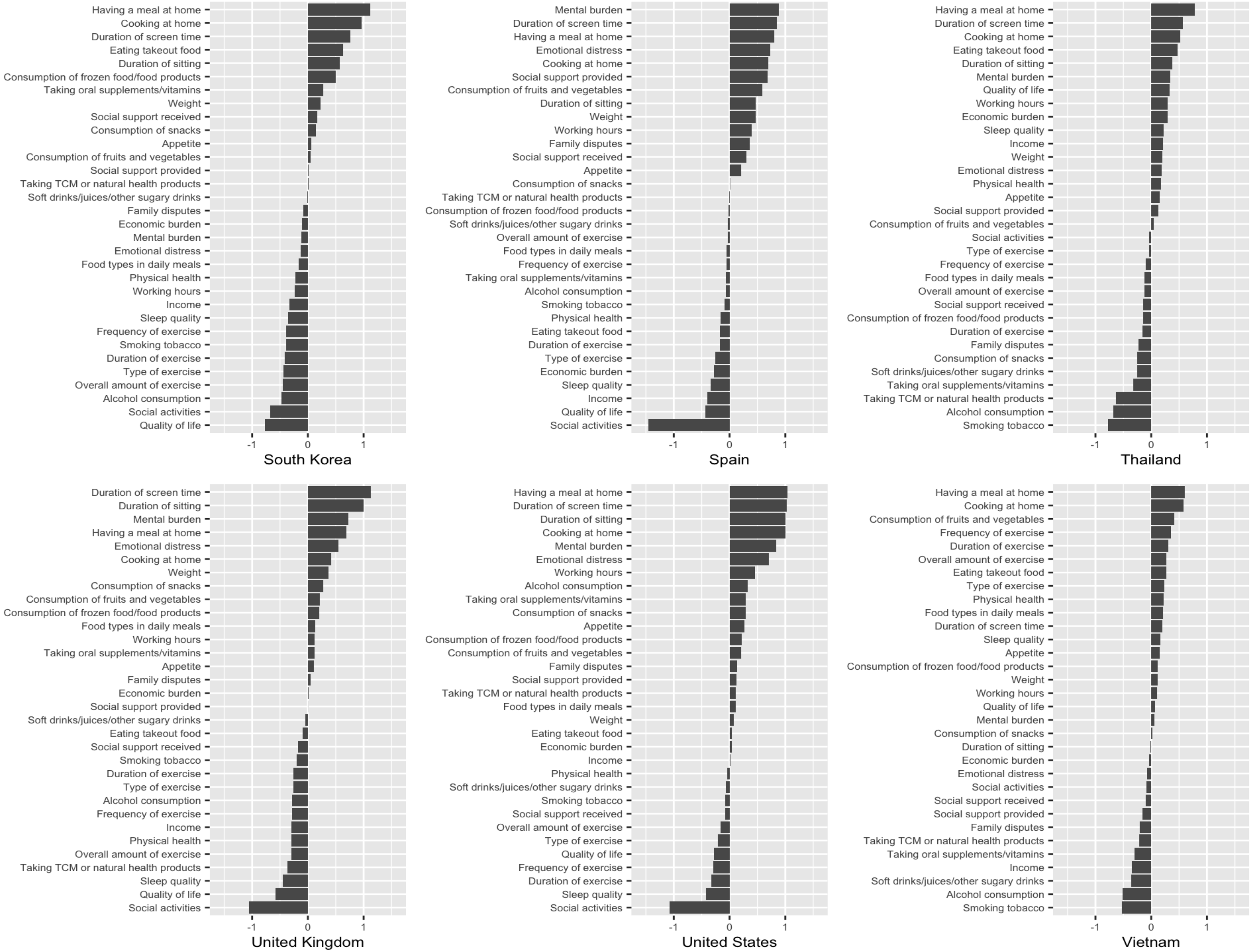
South Korea
Having a meal at home
Cooking at home
Duration of screen time
Eating takeout food
Duration of sitting
Consumption of frozen food/food products
Taking oral supplements/vitamins
Weight
Social support received
Consumption of snacks
Appetite
Consumption of fruits and vegetables
Social support provided
Taking TCM or natural health products
Soft drinks/juices/other sugary drinks
Family disputes
Economic burden
Mental burden
Emotional distress
Food types in daily meals
Physical health
Working hours
Income
Sleep quality
Frequency of exercise
Smoking tobacco
Duration of exercise
Type of exercise
Overall amount of exercise
Alcohol consumption
Social activities
Quality of life
-1
0
1
Spain
Mental burden
Duration of screen time
Having a meal at home
Emotional distress
Cooking at home
Social support provided
Consumption of fruits and vegetables
Duration of sitting
Weight
Working hours
Family disputes
Social support received
Appetite
Consumption of snacks
Taking TCM or natural health products
Consumption of frozen food/food products
Soft drinks/juices/other sugary drinks
Overall amount of exercise
Food types in daily meals
Frequency of exercise
Taking oral supplements/vitamins
Alcohol consumption
Smoking tobacco
Physical health
Eating takeout food
Duration of exercise
Type of exercise
Economic burden
Sleep quality
Income
Quality of life
Social activities
Thailand
Having a meal at home
Duration of screen time
Cooking at home
Eating takeout food
Duration of sitting
Mental burden
Quality of life
Working hours
Economic burden
Sleep quality
Income
Weight
Emotional distress
Physical health
Appetite
Social support provided
Consumption of fruits and vegetables
Social activities
Type of exercise
Frequency of exercise
Food types in daily meals
Overall amount of exercise
Social support received
Consumption of frozen food/food products
Duration of exercise
Family disputes
Consumption of snacks
Soft drinks/juices/other sugary drinks
Taking oral supplements/vitamins
Taking TCM or natural health products
Alcohol consumption
Smoking tobacco
United Kingdom
Duration of screen time
Duration of sitting
Mental burden
Having a meal at home
Emotional distress
Cooking at home
Weight
Consumption of snacks
Consumption of fruits and vegetables
Consumption of frozen food/food products
Food types in daily meals
Working hours
Taking oral supplements/vitamins
Appetite
Family disputes
Economic burden
Social support provided
Soft drinks/juices/other sugary drinks
Eating takeout food
Social support received
Smoking tobacco
Duration of exercise
Type of exercise
Alcohol consumption
Frequency of exercise
Income
Physical health
Overall amount of exercise
Taking TCM or natural health products
Sleep quality
Quality of life
Social activities
United States
Having a meal at home
Duration of screen time
Duration of sitting
Cooking at home
Mental burden
Emotional distress
Working hours
Alcohol consumption
Taking oral supplements/vitamins
Consumption of snacks
Appetite
Consumption of frozen food/food products
Consumption of fruits and vegetables
Family disputes
Social support provided
Taking TCM or natural health products
Food types in daily meals
Weight
Eating takeout food
Economic burden
Income
Physical health
Soft drinks/juices/other sugary drinks
Smoking tobacco
Social support received
Overall amount of exercise
Type of exercise
Quality of life
Frequency of exercise
Duration of exercise
Sleep quality
Social activities
Vietnam
Having a meal at home
Cooking at home
Consumption of fruits and vegetables
Frequency of exercise
Duration of exercise
Overall amount of exercise
Eating takeout food
Type of exercise
Physical health
Food types in daily meals
Duration of screen time
Sleep quality
Appetite
Consumption of frozen food/food products
Weight
Working hours
Quality of life
Mental burden
Consumption of snacks
Duration of sitting
Economic burden
Emotional distress
Social activities
Social support received
Social support provided
Family disputes
Taking TCM or natural health products
Taking oral supplements/vitamins
Income
Soft drinks/juices/other sugary drinks
Alcohol consumption
Smoking tobacco

b. Comparison of respondents' weighted mean of COVID-19's impact on lifestyles and health-related issues across countries.

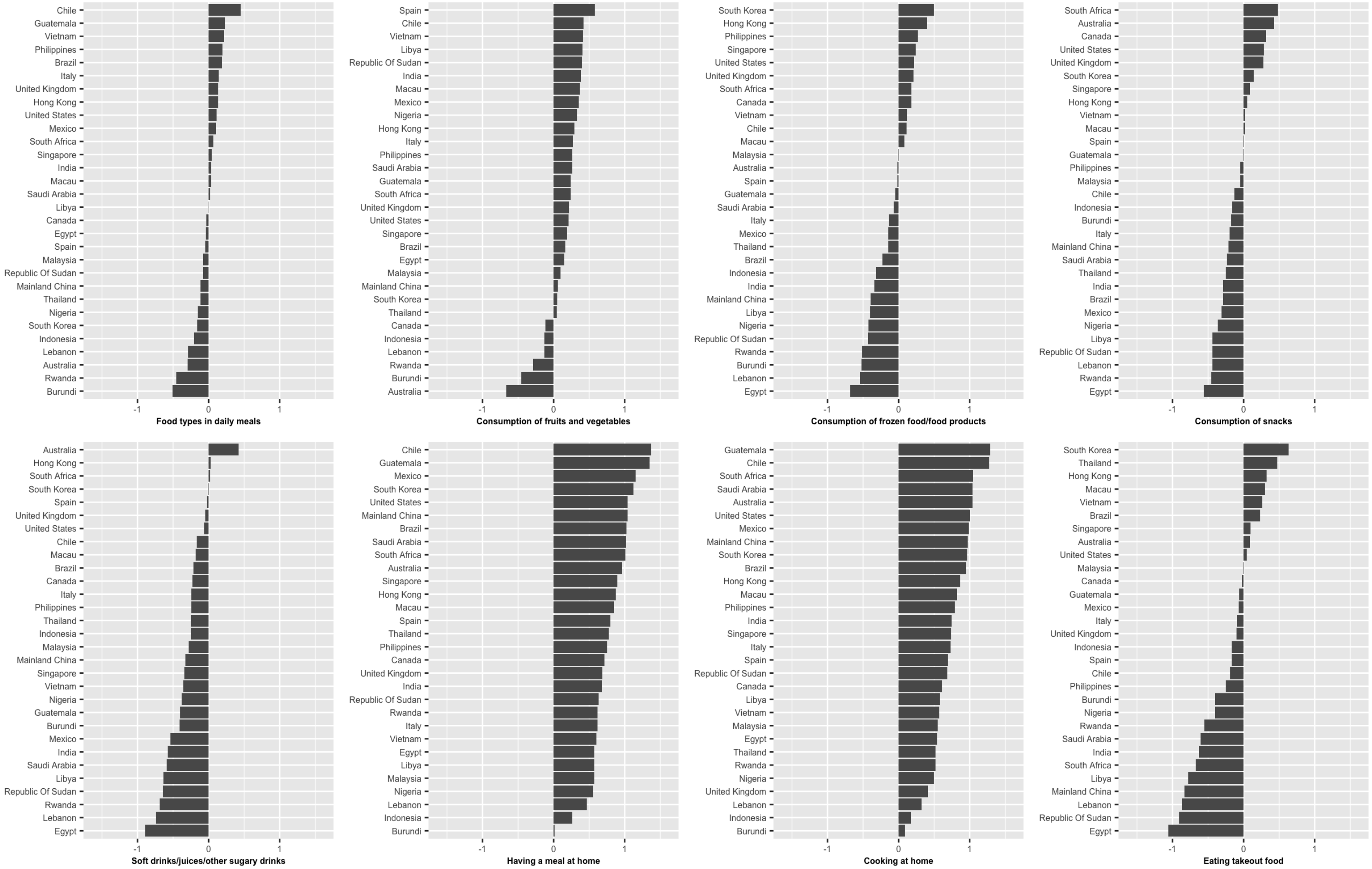

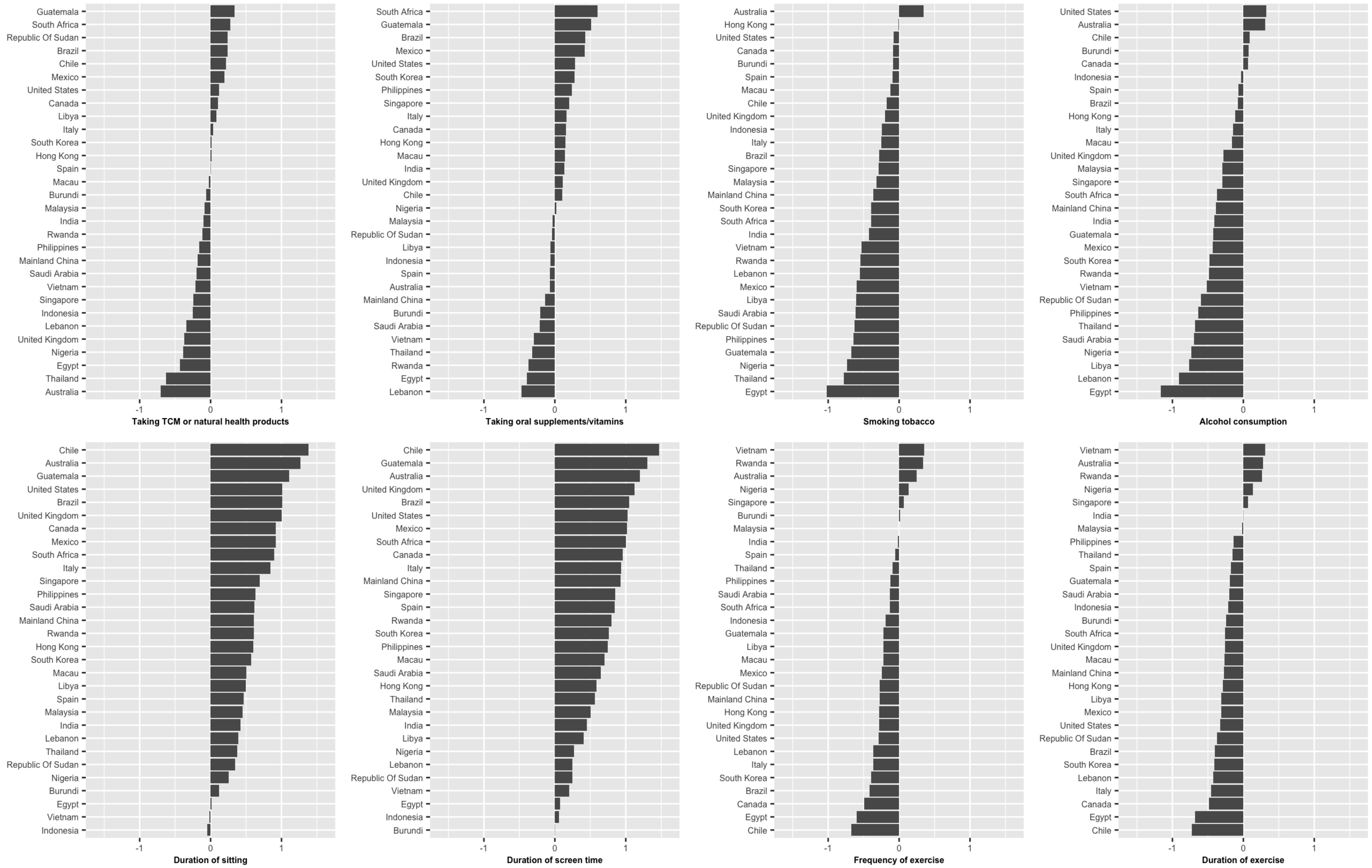
Guatemala
South Africa
Republic Of Sudan
Brazil
Chile
Mexico
United States
Canada
Libya
Italy
South Korea
Hong Kong
Spain
Macau
Burundi
Malaysia
India
Rwanda
Philippines
Mainland China
Saudi Arabia
Vietnam
Singapore
Indonesia
Lebanon
United Kingdom
Nigeria
Egypt
Thailand
Australia
-1
0
1
Taking TCM or natural health products
South Africa
Guatemala
Brazil
Mexico
United States
South Korea
Philippines
Singapore
Italy
Canada
Hong Kong
Macau
India
United Kingdom
Chile
Nigeria
Malaysia
Republic Of Sudan
Libya
Indonesia
Spain
Australia
Mainland China
Burundi
Saudi Arabia
Vietnam
Thailand
Rwanda
Egypt
Lebanon
-1
0
1
Taking oral supplements/vitamins
Australia
Hong Kong
United States
Canada
Burundi
Spain
Macau
Chile
United Kingdom
Indonesia
Italy
Brazil
Singapore
Malaysia
Mainland China
South Korea
South Africa
India
Vietnam
Rwanda
Lebanon
Mexico
Libya
Saudi Arabia
Republic Of Sudan
Philippines
Guatemala
Nigeria
Thailand
Egypt
-1
0
1
Smoking tobacco
United States
Australia
Chile
Burundi
Canada
Indonesia
Spain
Brazil
Hong Kong
Italy
Macau
United Kingdom
Malaysia
Singapore
South Africa
Mainland China
India
Guatemala
Mexico
South Korea
Rwanda
Vietnam
Republic Of Sudan
Philippines
Thailand
Saudi Arabia
Nigeria
Libya
Lebanon
Egypt
-1
0
1
Alcohol consumption
Chile
Australia
Guatemala
United States
Brazil
United Kingdom
Canada
Mexico
South Africa
Italy
Singapore
Philippines
Saudi Arabia
Mainland China
Rwanda
Hong Kong
South Korea
Macau
Libya
Spain
Malaysia
India
Lebanon
Thailand
Republic Of Sudan
Nigeria
Burundi
Egypt
Vietnam
Indonesia
-1
0
1
Duration of sitting
Chile
Guatemala
Australia
United Kingdom
Brazil
United States
Mexico
South Africa
Canada
Italy
Mainland China
Singapore
Spain
Rwanda
South Korea
Philippines
Macau
Saudi Arabia
Hong Kong
Thailand
Malaysia
India
Libya
Nigeria
Lebanon
Republic Of Sudan
Vietnam
Egypt
Indonesia
Burundi
-1
0
1
Duration of screen time
Vietnam
Rwanda
Australia
Nigeria
Singapore
Burundi
Malaysia
India
Spain
Thailand
Philippines
Saudi Arabia
South Africa
Indonesia
Guatemala
Libya
Macau
Mexico
Republic Of Sudan
Mainland China
Hong Kong
United Kingdom
United States
Lebanon
Italy
South Korea
Brazil
Canada
Egypt
Chile
-1
0
1
Frequency of exercise
Vietnam
Australia
Rwanda
Nigeria
Singapore
India
Malaysia
Philippines
Thailand
Spain
Guatemala
Saudi Arabia
Indonesia
Burundi
South Africa
United Kingdom
Macau
Mainland China
Hong Kong
Libya
Mexico
United States
Republic Of Sudan
Brazil
South Korea
Lebanon
Italy
Canada
Egypt
Chile
-1
0
1
Duration of exercise

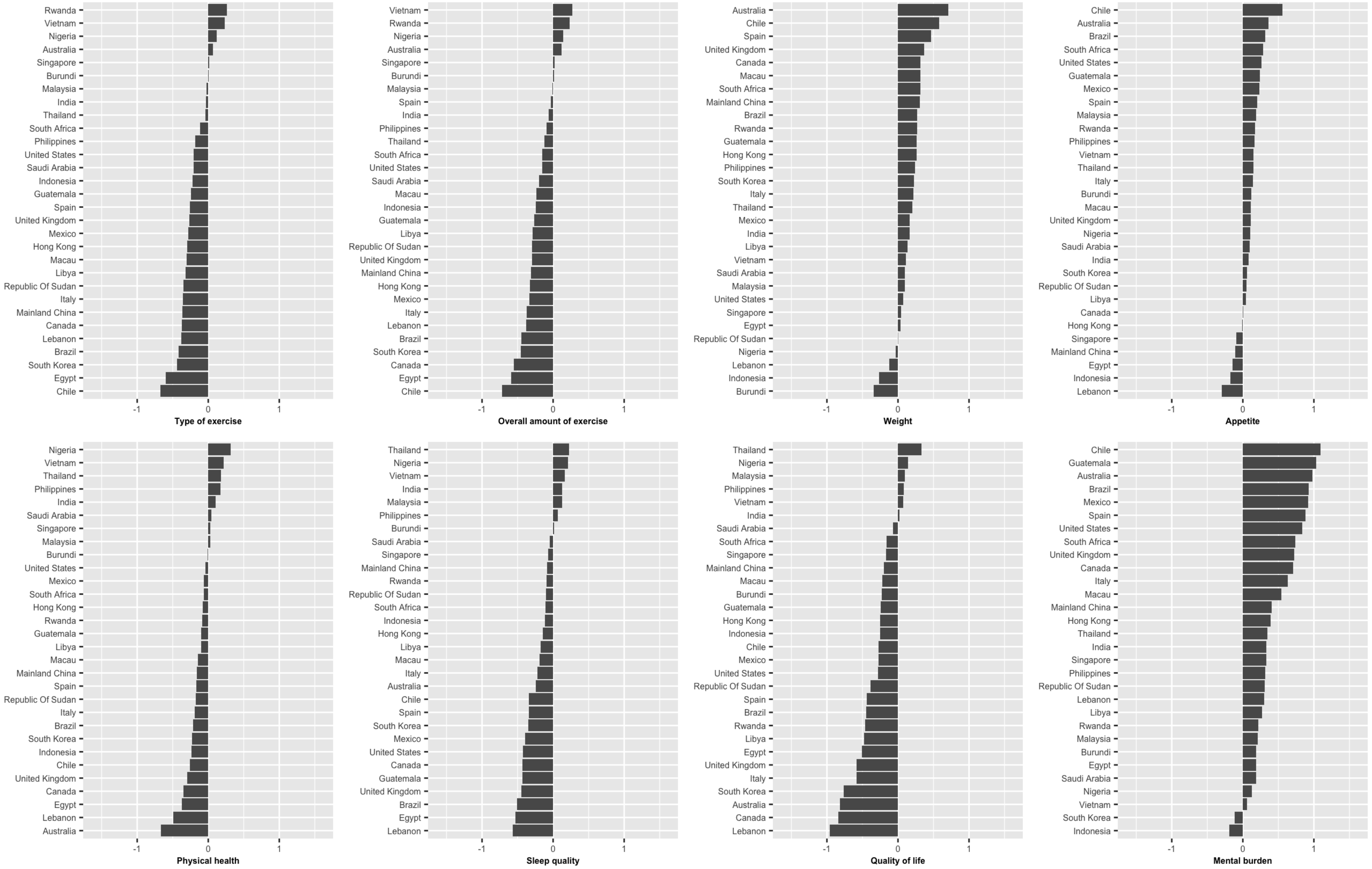
Rwanda
Vietnam
Nigeria
Australia
Singapore
Burundi
Malaysia
India
Thailand
South Africa
Philippines
United States
Saudi Arabia
Indonesia
Guatemala
Spain
United Kingdom
Mexico
Hong Kong
Macau
Libya
Republic Of Sudan
Italy
Mainland China
Canada
Lebanon
Brazil
South Korea
Egypt
Chile
-1
0
1
Type of exercise
Vietnam
Rwanda
Nigeria
Australia
Singapore
Burundi
Malaysia
Spain
India
Philippines
Thailand
South Africa
United States
Saudi Arabia
Macau
Indonesia
Guatemala
Libya
Republic Of Sudan
United Kingdom
Mainland China
Hong Kong
Mexico
Italy
Lebanon
Brazil
South Korea
Canada
Egypt
Chile
-1
0
1
Overall amount of exercise
Australia
Chile
Spain
United Kingdom
Canada
Macau
South Africa
Mainland China
Brazil
Rwanda
Guatemala
Hong Kong
Philippines
South Korea
Italy
Thailand
Mexico
India
Libya
Vietnam
Saudi Arabia
Malaysia
United States
Singapore
Egypt
Republic Of Sudan
Nigeria
Lebanon
Indonesia
Burundi
-1
0
1
Weight
Chile
Australia
Brazil
South Africa
United States
Guatemala
Mexico
Spain
Malaysia
Rwanda
Philippines
Vietnam
Thailand
Italy
Burundi
Macau
United Kingdom
Nigeria
Saudi Arabia
India
South Korea
Republic Of Sudan
Libya
Canada
Hong Kong
Singapore
Mainland China
Egypt
Indonesia
Lebanon
-1
0
1
Appetite
Nigeria
Vietnam
Thailand
Philippines
India
Saudi Arabia
Singapore
Malaysia
Burundi
United States
Mexico
South Africa
Hong Kong
Rwanda
Guatemala
Libya
Macau
Mainland China
Spain
Republic Of Sudan
Italy
Brazil
South Korea
Indonesia
Chile
United Kingdom
Canada
Egypt
Lebanon
Australia
-1
0
1
Physical health
Thailand
Nigeria
Vietnam
India
Malaysia
Philippines
Burundi
Saudi Arabia
Singapore
Mainland China
Rwanda
Republic Of Sudan
South Africa
Indonesia
Hong Kong
Libya
Macau
Italy
Australia
Chile
Spain
South Korea
Mexico
United States
Canada
Guatemala
United Kingdom
Brazil
Egypt
Lebanon
-1
0
1
Sleep quality
Thailand
Nigeria
Malaysia
Philippines
Vietnam
India
Saudi Arabia
South Africa
Singapore
Mainland China
Macau
Burundi
Guatemala
Hong Kong
Indonesia
Chile
Mexico
United States
Republic Of Sudan
Spain
Brazil
Rwanda
Libya
Egypt
United Kingdom
Italy
South Korea
Australia
Canada
Lebanon
-1
0
1
Quality of life
Chile
Guatemala
Australia
Brazil
Mexico
Spain
United States
South Africa
United Kingdom
Canada
Italy
Macau
Mainland China
Hong Kong
Thailand
India
Singapore
Philippines
Republic Of Sudan
Lebanon
Libya
Rwanda
Malaysia
Burundi
Egypt
Saudi Arabia
Nigeria
Vietnam
South Korea
Indonesia
-1
0
1
Mental burden

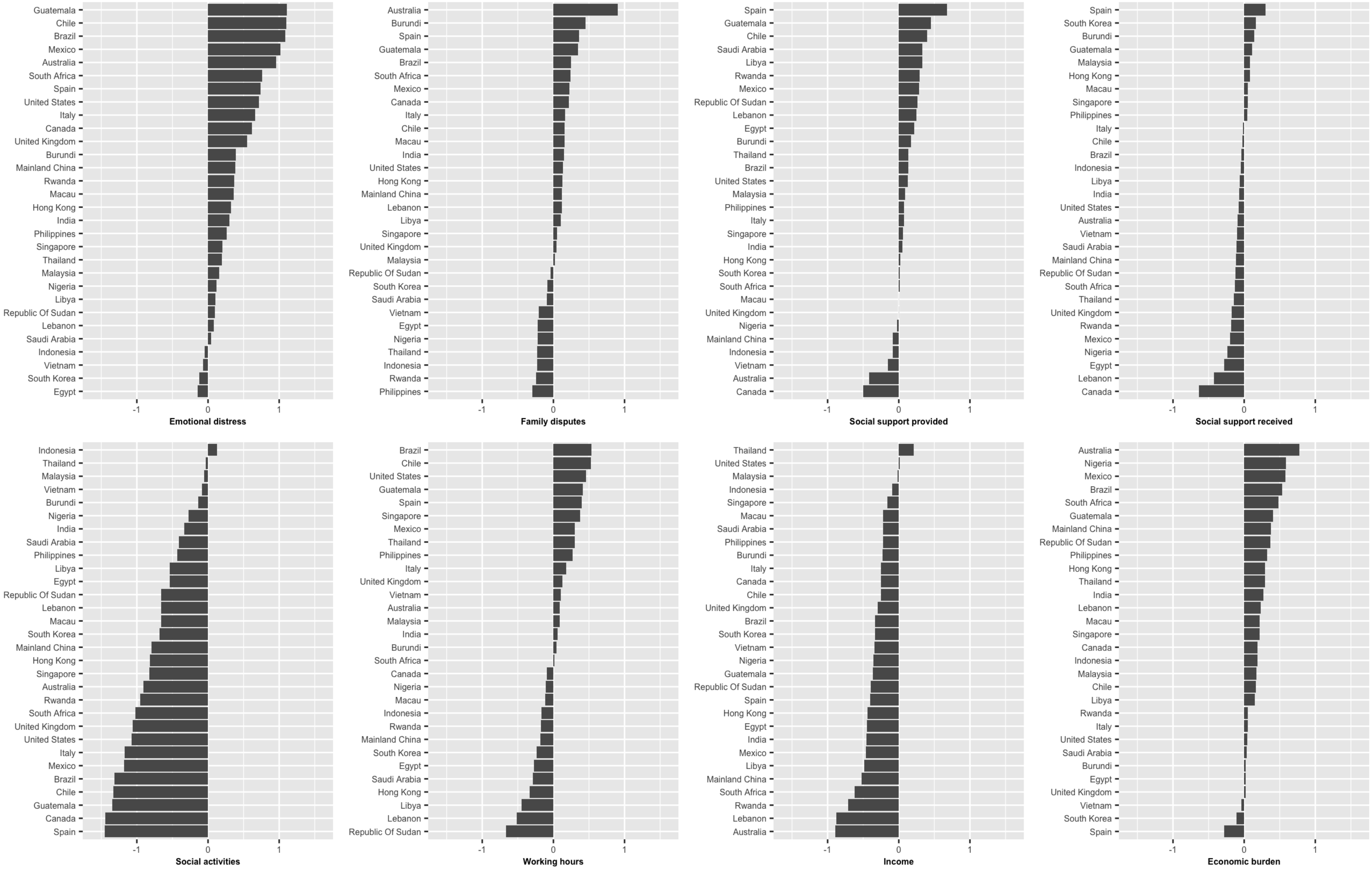
Guatemala
Chile
Brazil
Mexico
Australia
South Africa
Spain
United States
Italy
Canada
United Kingdom
Burundi
Mainland China
Rwanda
Macau
Hong Kong
India
Philippines
Singapore
Thailand
Malaysia
Nigeria
Libya
Republic Of Sudan
Lebanon
Saudi Arabia
Indonesia
Vietnam
South Korea
Egypt
-1
0
1
Emotional distress
Australia
Burundi
Spain
Guatemala
Brazil
South Africa
Mexico
Canada
Italy
Chile
Macau
India
United States
Hong Kong
Mainland China
Lebanon
Libya
Singapore
United Kingdom
Malaysia
Republic Of Sudan
South Korea
Saudi Arabia
Vietnam
Egypt
Nigeria
Thailand
Indonesia
Rwanda
Philippines
-1
0
1
Family disputes
Spain
Guatemala
Chile
Saudi Arabia
Libya
Rwanda
Mexico
Republic Of Sudan
Lebanon
Egypt
Burundi
Thailand
Brazil
United States
Malaysia
Philippines
Italy
Singapore
India
Hong Kong
South Korea
South Africa
Macau
United Kingdom
Nigeria
Mainland China
Indonesia
Vietnam
Australia
Canada
-1
0
1
Social support provided
Spain
South Korea
Burundi
Guatemala
Malaysia
Hong Kong
Macau
Singapore
Philippines
Italy
Chile
Brazil
Indonesia
Libya
India
United States
Australia
Vietnam
Saudi Arabia
Mainland China
Republic Of Sudan
South Africa
Thailand
United Kingdom
Rwanda
Mexico
Nigeria
Egypt
Lebanon
Canada
-1
0
1
Social support received
Indonesia
Thailand
Malaysia
Vietnam
Burundi
Nigeria
India
Saudi Arabia
Philippines
Libya
Egypt
Republic Of Sudan
Lebanon
Macau
South Korea
Mainland China
Hong Kong
Singapore
Australia
Rwanda
South Africa
United Kingdom
United States
Italy
Mexico
Brazil
Chile
Guatemala
Canada
Spain
-1
0
1
Social activities
Brazil
Chile
United States
Guatemala
Spain
Singapore
Mexico
Thailand
Philippines
Italy
United Kingdom
Vietnam
Australia
Malaysia
India
Burundi
South Africa
Canada
Nigeria
Macau
Indonesia
Rwanda
Mainland China
South Korea
Egypt
Saudi Arabia
Hong Kong
Libya
Lebanon
Republic Of Sudan
-1
0
1
Working hours
Thailand
United States
Malaysia
Indonesia
Singapore
Macau
Saudi Arabia
Philippines
Burundi
Italy
Canada
Chile
United Kingdom
Brazil
South Korea
Vietnam
Nigeria
Guatemala
Republic Of Sudan
Spain
Hong Kong
Egypt
India
Mexico
Libya
Mainland China
South Africa
Rwanda
Lebanon
Australia
-1
0
1
Income
Australia
Nigeria
Mexico
Brazil
South Africa
Guatemala
Mainland China
Republic Of Sudan
Philippines
Hong Kong
Thailand
India
Lebanon
Macau
Singapore
Canada
Indonesia
Malaysia
Chile
Libya
Rwanda
Italy
United States
Saudi Arabia
Burundi
Egypt
United Kingdom
Vietnam
South Korea
Spain
-1
0
1
Economic burden

c. Weighted mean of COVID-19's impact on lifestyles and health-related issues by region.

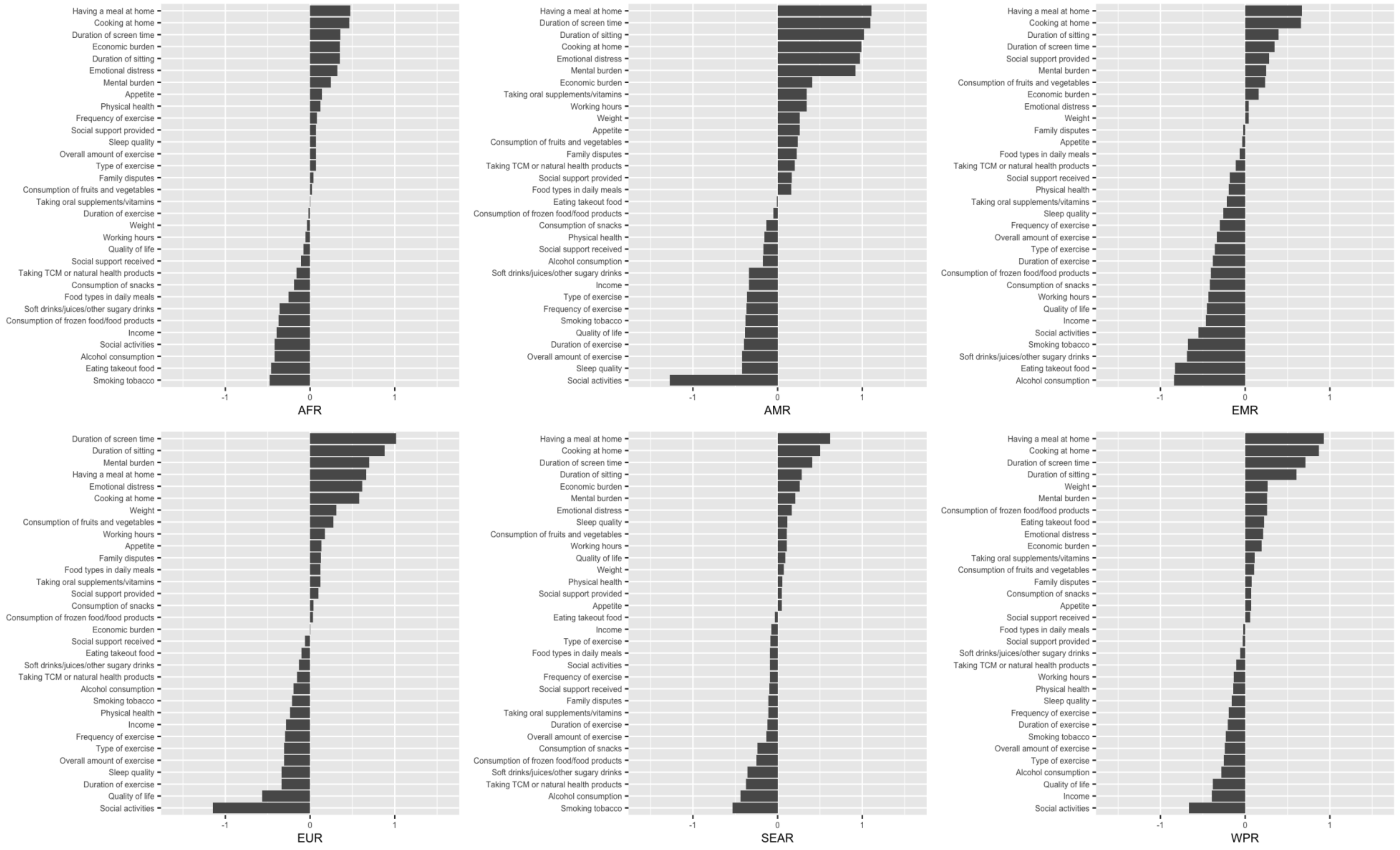

d. Weighted mean of COVID-19's impact on lifestyles and health-related issues by economic development level.

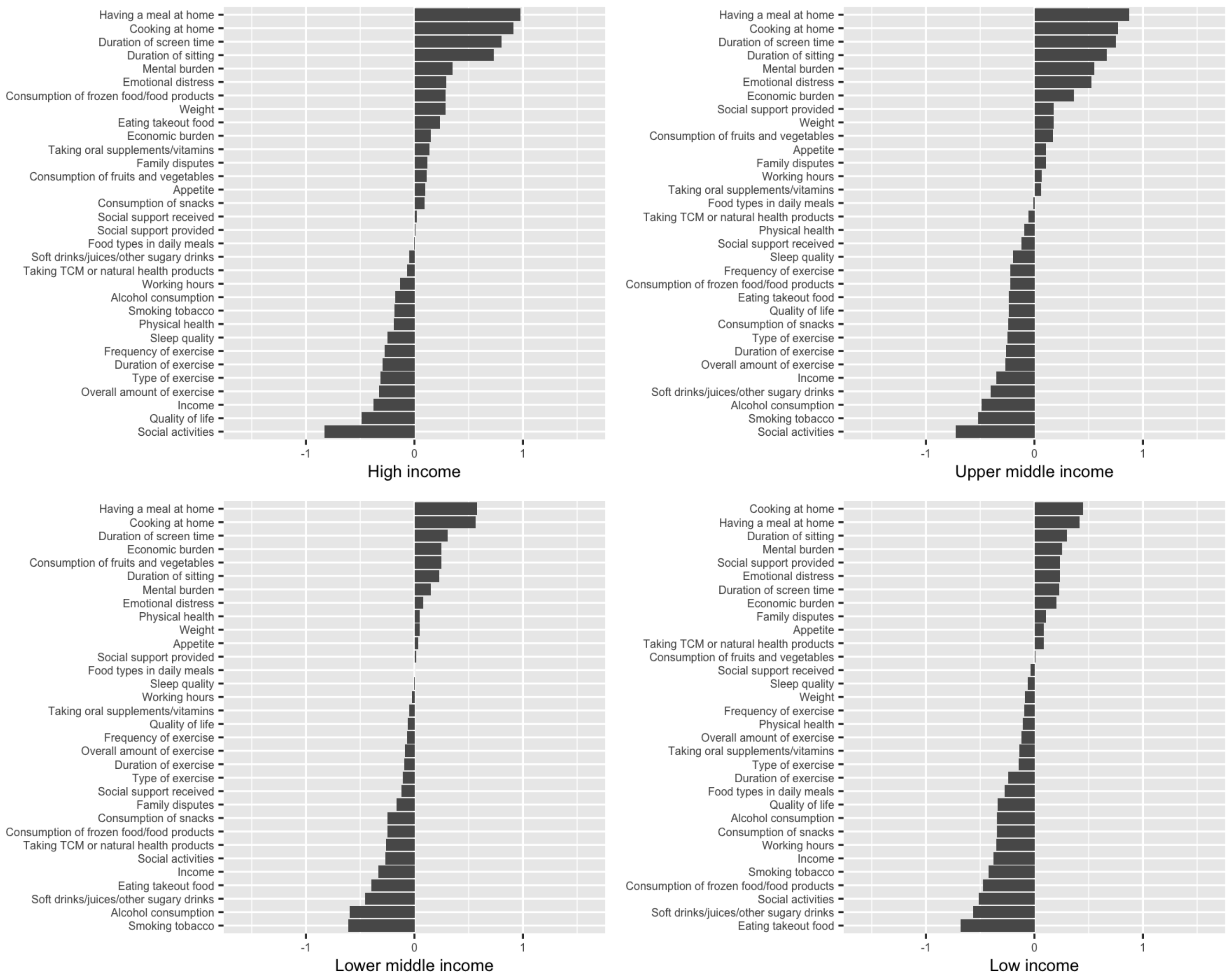

e. Weighted mean of COVID-19's impact on lifestyles and health-related issues by COVID-19 severity level.

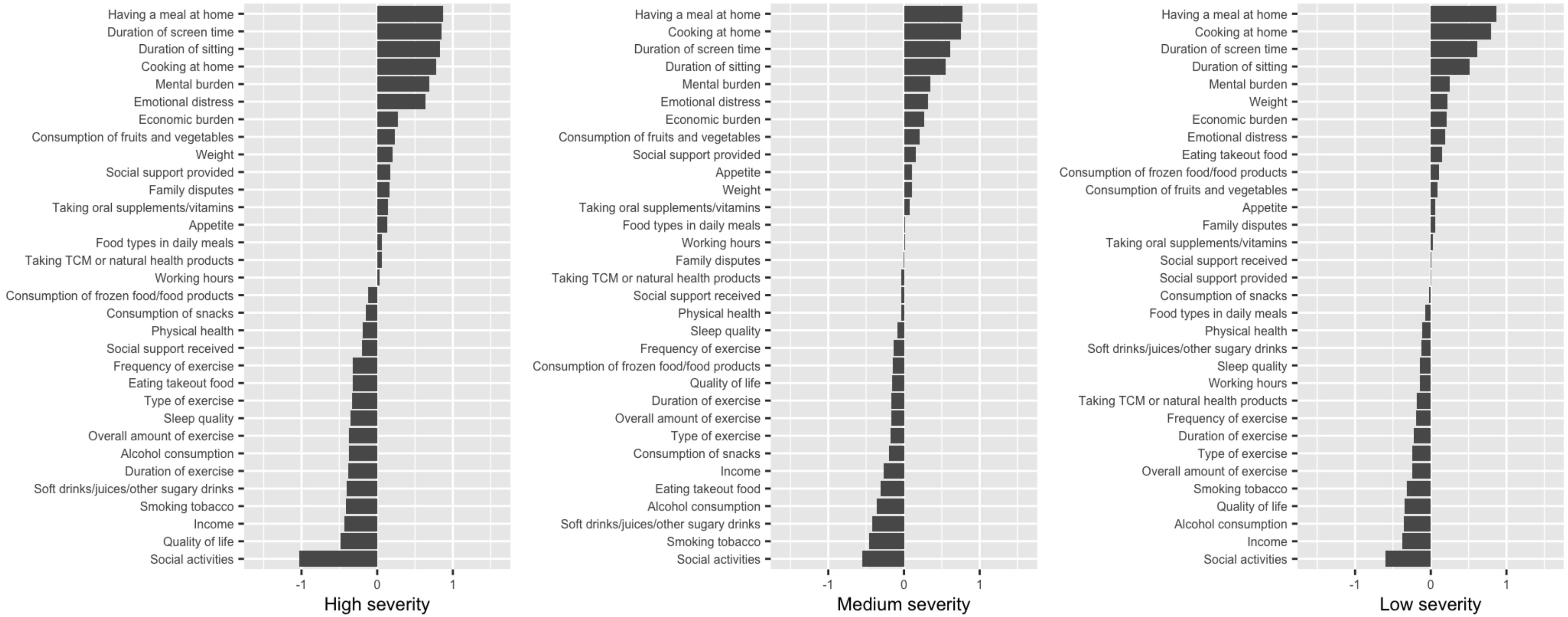

f. Weighted mean of preference for future preparations by country.

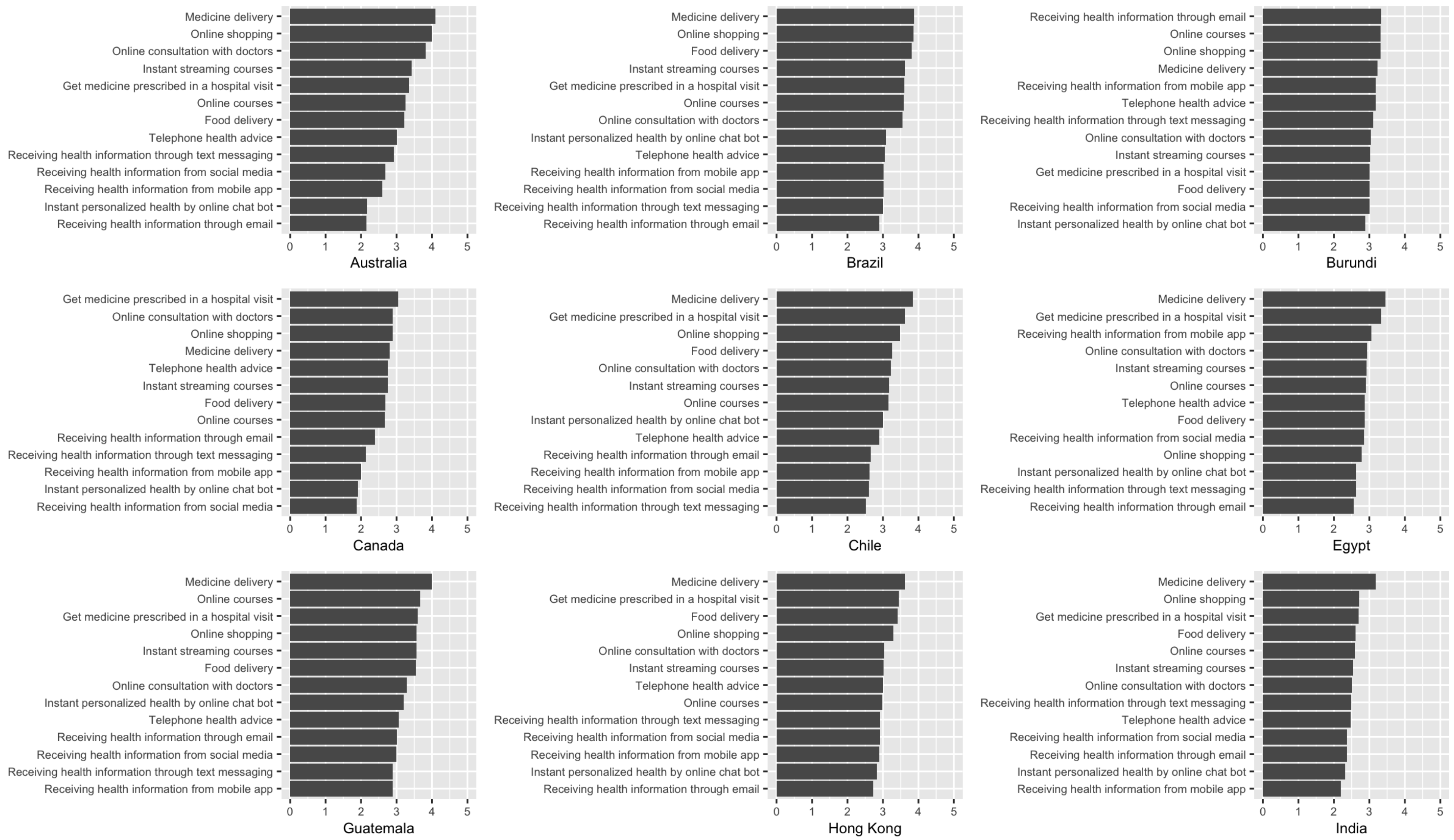

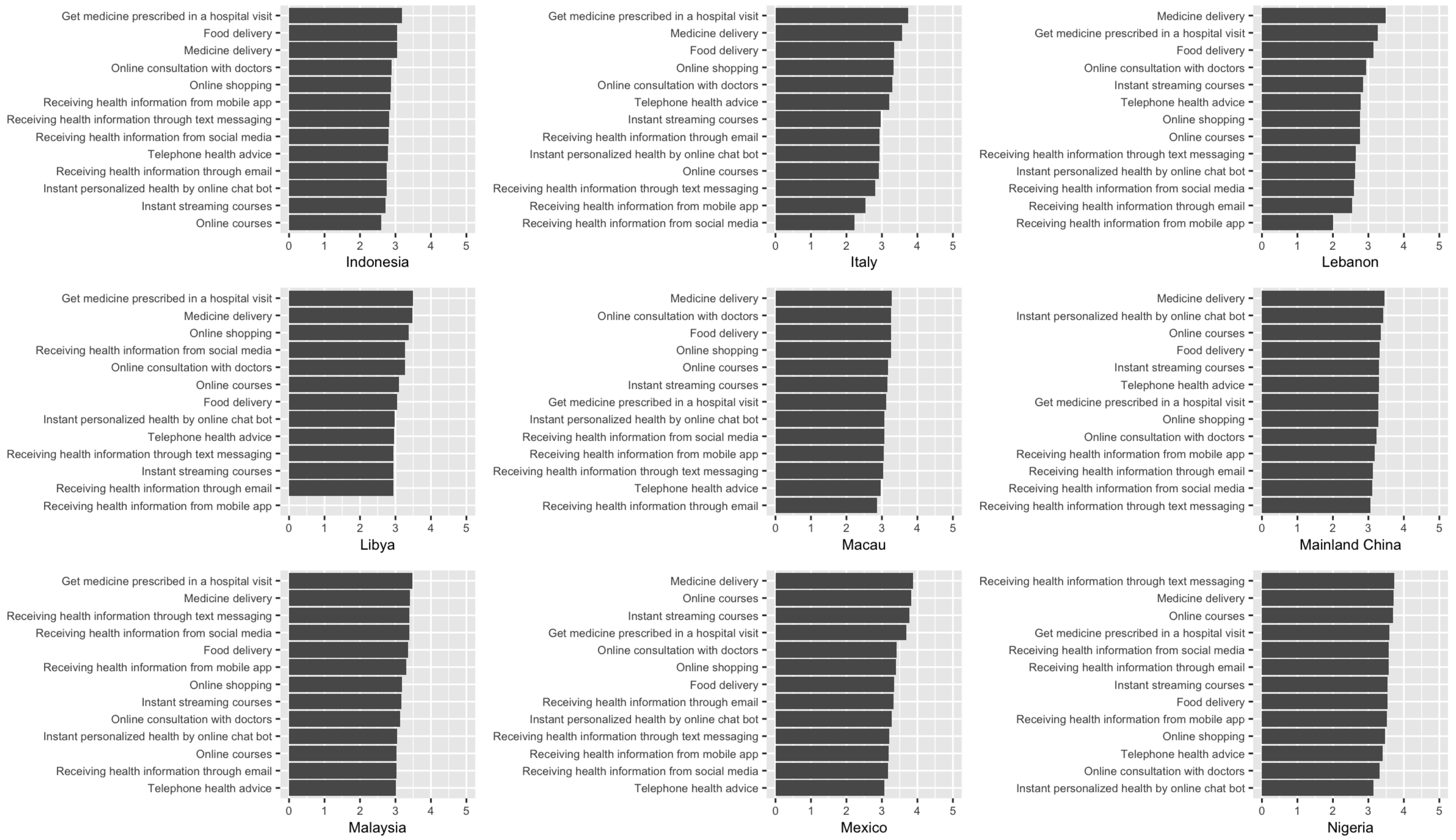
Get medicine prescribed in a hospital visit
Food delivery
Medicine delivery
Online consultation with doctors
Online shopping
Receiving health information from mobile app
Receiving health information through text messaging
Receiving health information from social media
Telephone health advice
Receiving health information through email
Instant personalized health by online chat bot
Instant streaming courses
Online courses
0 1 2 3 4 5
Indonesia
Get medicine prescribed in a hospital visit
Medicine delivery
Food delivery
Online shopping
Online consultation with doctors
Telephone health advice
Instant streaming courses
Receiving health information through email
Instant personalized health by online chat bot
Online courses
Receiving health information through text messaging
Receiving health information from mobile app
Receiving health information from social media
0 1 2 3 4 5
Italy
Medicine delivery
Get medicine prescribed in a hospital visit
Food delivery
Online consultation with doctors
Instant streaming courses
Telephone health advice
Online shopping
Online courses
Receiving health information through text messaging
Instant personalized health by online chat bot
Receiving health information from social media
Receiving health information through email
Receiving health information from mobile app
0 1 2 3 4 5
Lebanon
Get medicine prescribed in a hospital visit
Medicine delivery
Online shopping
Receiving health information from social media
Online consultation with doctors
Online courses
Food delivery
Instant personalized health by online chat bot
Telephone health advice
Receiving health information through text messaging
Instant streaming courses
Receiving health information through email
Receiving health information from mobile app
0 1 2 3 4 5
Libya
Medicine delivery
Online consultation with doctors
Food delivery
Online shopping
Online courses
Instant streaming courses
Get medicine prescribed in a hospital visit
Instant personalized health by online chat bot
Receiving health information from social media
Receiving health information from mobile app
Receiving health information through text messaging
Telephone health advice
Receiving health information through email
0 1 2 3 4 5
Macau
Medicine delivery
Instant personalized health by online chat bot
Online courses
Food delivery
Instant streaming courses
Telephone health advice
Get medicine prescribed in a hospital visit
Online shopping
Online consultation with doctors
Receiving health information from mobile app
Receiving health information through email
Receiving health information from social media
Receiving health information through text messaging
0 1 2 3 4 5
Mainland China
Get medicine prescribed in a hospital visit
Medicine delivery
Receiving health information through text messaging
Receiving health information from social media
Food delivery
Receiving health information from mobile app
Online shopping
Instant streaming courses
Online consultation with doctors
Instant personalized health by online chat bot
Online courses
Receiving health information through email
Telephone health advice
0 1 2 3 4 5
Malaysia
Medicine delivery
Online courses
Instant streaming courses
Get medicine prescribed in a hospital visit
Online consultation with doctors
Online shopping
Food delivery
Receiving health information through email
Instant personalized health by online chat bot
Receiving health information through text messaging
Receiving health information from mobile app
Receiving health information from social media
Telephone health advice
0 1 2 3 4 5
Mexico
Receiving health information through text messaging
Medicine delivery
Online courses
Get medicine prescribed in a hospital visit
Receiving health information from social media
Receiving health information through email
Instant streaming courses
Food delivery
Receiving health information from mobile app
Online shopping
Telephone health advice
Online consultation with doctors
Instant personalized health by online chat bot
0 1 2 3 4 5
Nigeria

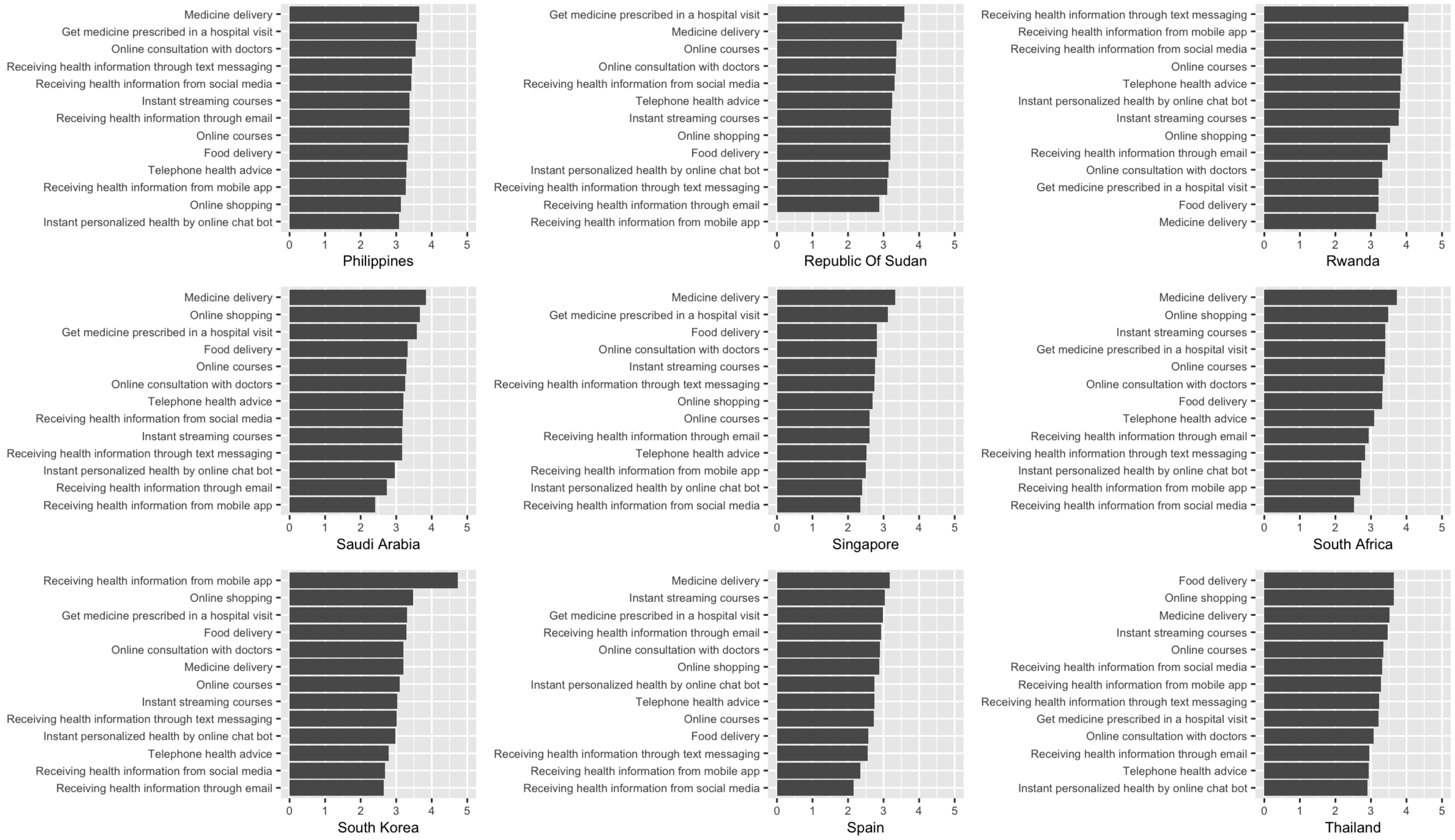
Medicine delivery
Get medicine prescribed in a hospital visit
Online consultation with doctors
Receiving health information through text messaging
Receiving health information from social media
Instant streaming courses
Receiving health information through email
Online courses
Food delivery
Telephone health advice
Receiving health information from mobile app
Online shopping
Instant personalized health by online chat bot
0 1 2 3 4 5
Philippines
Get medicine prescribed in a hospital visit
Medicine delivery
Online courses
Online consultation with doctors
Receiving health information from social media
Telephone health advice
Instant streaming courses
Online shopping
Food delivery
Instant personalized health by online chat bot
Receiving health information through text messaging
Receiving health information through email
Receiving health information from mobile app
0 1 2 3 4 5
Republic Of Sudan
Receiving health information through text messaging
Receiving health information from mobile app
Receiving health information from social media
Online courses
Telephone health advice
Instant personalized health by online chat bot
Instant streaming courses
Online shopping
Receiving health information through email
Online consultation with doctors
Get medicine prescribed in a hospital visit
Food delivery
Medicine delivery
0 1 2 3 4 5
Rwanda
Medicine delivery
Online shopping
Get medicine prescribed in a hospital visit
Food delivery
Online courses
Online consultation with doctors
Telephone health advice
Receiving health information from social media
Instant streaming courses
Receiving health information through text messaging
Instant personalized health by online chat bot
Receiving health information through email
Receiving health information from mobile app
0 1 2 3 4 5
Saudi Arabia
Medicine delivery
Get medicine prescribed in a hospital visit
Food delivery
Online consultation with doctors
Instant streaming courses
Receiving health information through text messaging
Online shopping
Online courses
Receiving health information through email
Telephone health advice
Receiving health information from mobile app
Instant personalized health by online chat bot
Receiving health information from social media
0 1 2 3 4 5
Singapore
Medicine delivery
Online shopping
Instant streaming courses
Get medicine prescribed in a hospital visit
Online courses
Online consultation with doctors
Food delivery
Telephone health advice
Receiving health information through email
Receiving health information through text messaging
Instant personalized health by online chat bot
Receiving health information from mobile app
Receiving health information from social media
0 1 2 3 4 5
South Africa
Receiving health information from mobile app
Online shopping
Get medicine prescribed in a hospital visit
Food delivery
Online consultation with doctors
Medicine delivery
Online courses
Instant streaming courses
Receiving health information through text messaging
Instant personalized health by online chat bot
Telephone health advice
Receiving health information from social media
Receiving health information through email
0 1 2 3 4 5
South Korea
Medicine delivery
Instant streaming courses
Get medicine prescribed in a hospital visit
Receiving health information through email
Online consultation with doctors
Online shopping
Instant personalized health by online chat bot
Telephone health advice
Online courses
Food delivery
Receiving health information through text messaging
Receiving health information from mobile app
Receiving health information from social media
0 1 2 3 4 5
Spain
Food delivery
Online shopping
Medicine delivery
Instant streaming courses
Online courses
Receiving health information from social media
Receiving health information from mobile app
Receiving health information through text messaging
Get medicine prescribed in a hospital visit
Online consultation with doctors
Receiving health information through email
Telephone health advice
Instant personalized health by online chat bot
0 1 2 3 4 5
Thailand

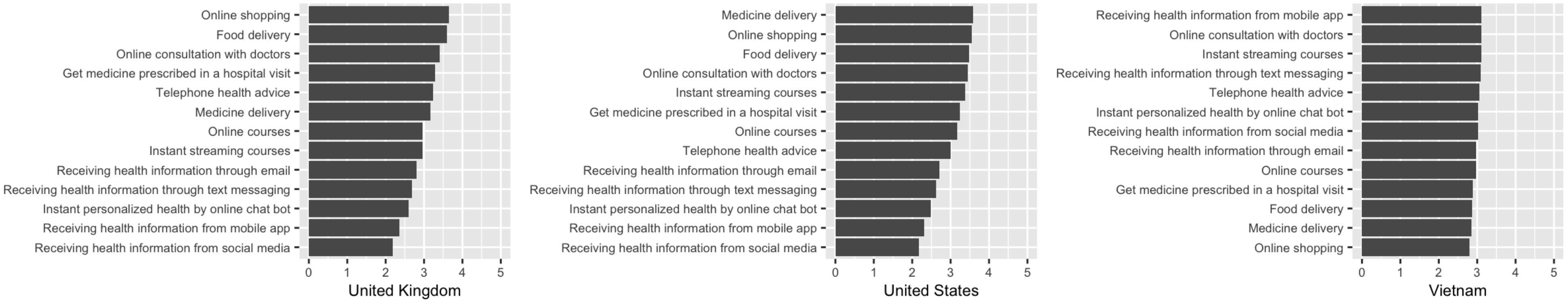
Online shopping
Food delivery
Online consultation with doctors
Get medicine prescribed in a hospital visit
Telephone health advice
Medicine delivery
Online courses
Instant streaming courses
Receiving health information through email
Receiving health information through text messaging
Instant personalized health by online chat bot
Receiving health information from mobile app
Receiving health information from social media
0 1 2 3 4 5
United Kingdom
Medicine delivery
Online shopping
Food delivery
Online consultation with doctors
Instant streaming courses
Get medicine prescribed in a hospital visit
Online courses
Telephone health advice
Receiving health information through email
Receiving health information through text messaging
Instant personalized health by online chat bot
Receiving health information from mobile app
Receiving health information from social media
0 1 2 3 4 5
United States
Receiving health information from mobile app
Online consultation with doctors
Instant streaming courses
Receiving health information through text messaging
Telephone health advice
Instant personalized health by online chat bot
Receiving health information from social media
Receiving health information through email
Online courses
Get medicine prescribed in a hospital visit
Food delivery
Medicine delivery
Online shopping
0 1 2 3 4 5
Vietnam

g. Comparison of respondents' weighted mean of preference for future preparations across countries.

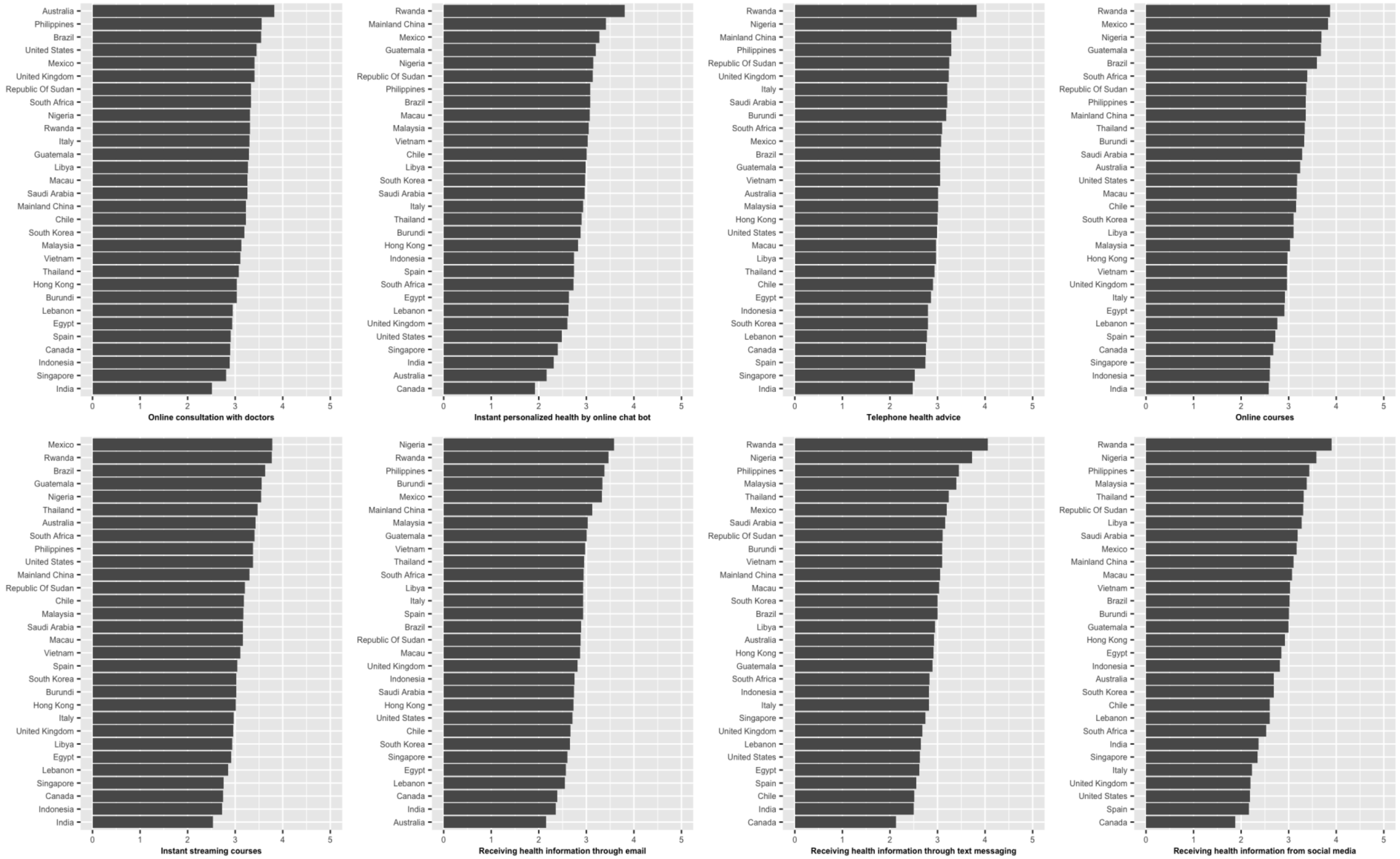
Australia
Philippines
Brazil
United States
Mexico
United Kingdom
Republic Of Sudan
South Africa
Nigeria
Rwanda
Italy
Guatemala
Libya
Macau
Saudi Arabia
Mainland China
Chile
South Korea
Malaysia
Vietnam
Thailand
Hong Kong
Burundi
Lebanon
Egypt
Spain
Canada
Indonesia
Singapore
India
0 1 2 3 4 5
Online consultation with doctors
Rwanda
Mainland China
Mexico
Guatemala
Nigeria
Republic Of Sudan
Philippines
Brazil
Macau
Malaysia
Vietnam
Chile
Libya
South Korea
Saudi Arabia
Italy
Thailand
Burundi
Hong Kong
Indonesia
Spain
South Africa
Egypt
Lebanon
United Kingdom
United States
Singapore
India
Australia
Canada
0 1 2 3 4 5
Instant personalized health by online chat bot
Rwanda
Nigeria
Mainland China
Philippines
Republic Of Sudan
United Kingdom
Italy
Saudi Arabia
Burundi
South Africa
Mexico
Brazil
Guatemala
Vietnam
Australia
Malaysia
Hong Kong
United States
Macau
Libya
Thailand
Chile
Egypt
Indonesia
South Korea
Lebanon
Canada
Spain
Singapore
India
0 1 2 3 4 5
Telephone health advice
Rwanda
Mexico
Nigeria
Guatemala
Brazil
South Africa
Republic Of Sudan
Philippines
Mainland China
Thailand
Burundi
Saudi Arabia
Australia
United States
Macau
Chile
South Korea
Libya
Malaysia
Hong Kong
Vietnam
United Kingdom
Italy
Egypt
Lebanon
Spain
Canada
Singapore
Indonesia
India
0 1 2 3 4 5
Online courses
Mexico
Rwanda
Brazil
Guatemala
Nigeria
Thailand
Australia
South Africa
Philippines
United States
Mainland China
Republic Of Sudan
Chile
Malaysia
Saudi Arabia
Macau
Vietnam
Spain
South Korea
Burundi
Hong Kong
Italy
United Kingdom
Libya
Egypt
Lebanon
Singapore
Canada
Indonesia
India
0 1 2 3 4 5
Instant streaming courses
Nigeria
Rwanda
Philippines
Burundi
Mexico
Mainland China
Malaysia
Guatemala
Vietnam
Thailand
South Africa
Libya
Italy
Spain
Brazil
Republic Of Sudan
Macau
United Kingdom
Indonesia
Saudi Arabia
Hong Kong
United States
Chile
South Korea
Singapore
Egypt
Lebanon
Canada
India
Australia
0 1 2 3 4 5
Receiving health information through email
Rwanda
Nigeria
Philippines
Malaysia
Thailand
Mexico
Saudi Arabia
Republic Of Sudan
Burundi
Vietnam
Mainland China
Macau
South Korea
Brazil
Libya
Australia
Hong Kong
Guatemala
South Africa
Indonesia
Italy
Singapore
United Kingdom
Lebanon
United States
Egypt
Spain
Chile
India
Canada
0 1 2 3 4 5
Receiving health information through text messaging
Rwanda
Nigeria
Philippines
Malaysia
Thailand
Republic Of Sudan
Libya
Saudi Arabia
Mexico
Mainland China
Macau
Vietnam
Brazil
Burundi
Guatemala
Hong Kong
Egypt
Indonesia
Australia
South Korea
Chile
Lebanon
South Africa
India
Singapore
Italy
United Kingdom
United States
Spain
Canada
0 1 2 3 4 5
Receiving health information from social media

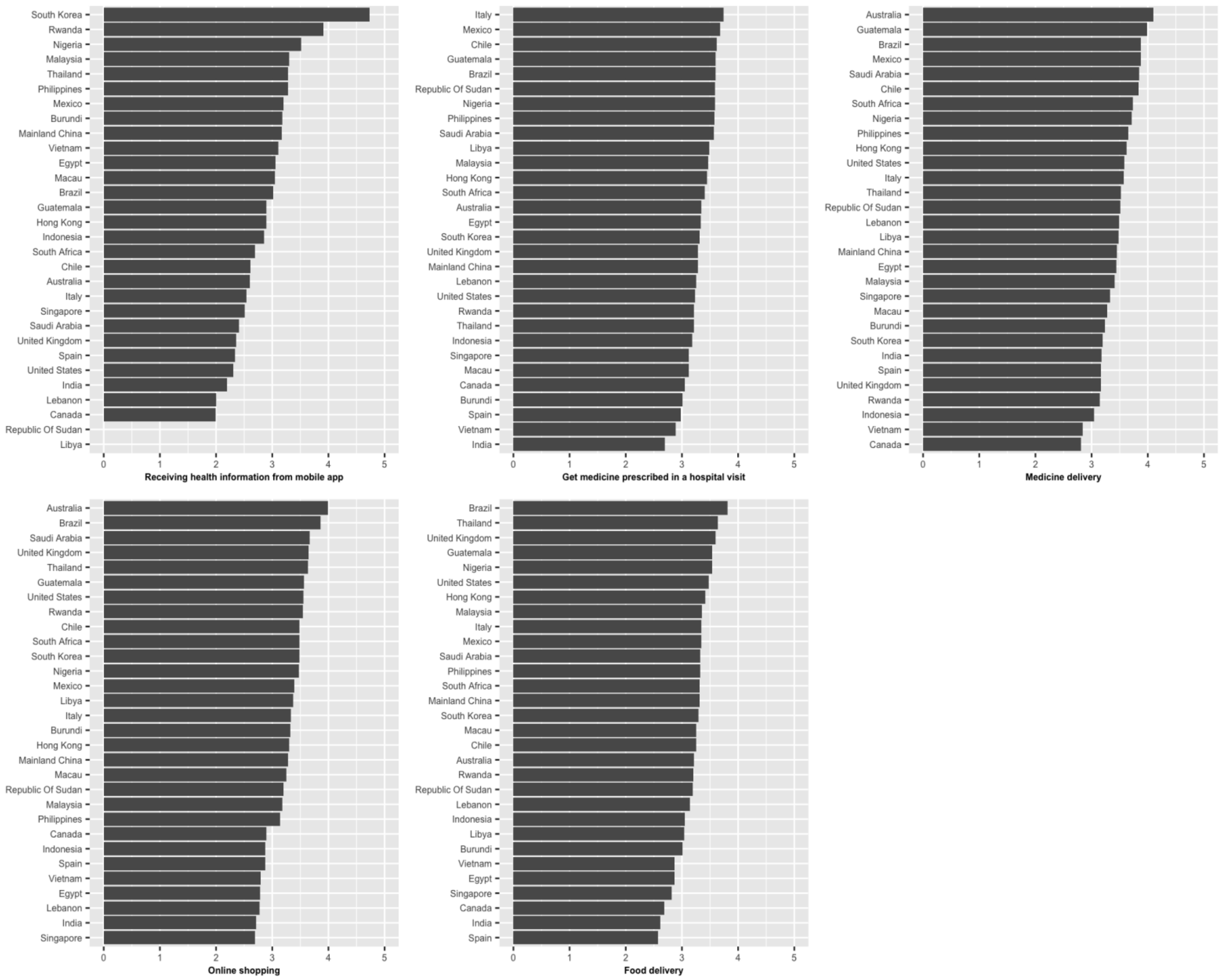
South Korea
Rwanda
Nigeria
Malaysia
Thailand
Philippines
Mexico
Burundi
Mainland China
Vietnam
Egypt
Macau
Brazil
Guatemala
Hong Kong
Indonesia
South Africa
Chile
Australia
Italy
Singapore
Saudi Arabia
United Kingdom
Spain
United States
India
Lebanon
Canada
Republic Of Sudan
Libya
0 1 2 3 4 5
Receiving health information from mobile app
Italy
Mexico
Chile
Guatemala
Brazil
Republic Of Sudan
Nigeria
Philippines
Saudi Arabia
Libya
Malaysia
Hong Kong
South Africa
Australia
Egypt
South Korea
United Kingdom
Mainland China
Lebanon
United States
Rwanda
Thailand
Indonesia
Singapore
Macau
Canada
Burundi
Spain
Vietnam
India
0 1 2 3 4 5
Get medicine prescribed in a hospital visit
Australia
Guatemala
Brazil
Mexico
Saudi Arabia
Chile
South Africa
Nigeria
Philippines
Hong Kong
United States
Italy
Thailand
Republic Of Sudan
Lebanon
Libya
Mainland China
Egypt
Malaysia
Singapore
Macau
Burundi
South Korea
India
Spain
United Kingdom
Rwanda
Indonesia
Vietnam
Canada
0 1 2 3 4 5
Medicine delivery
Australia
Brazil
Saudi Arabia
United Kingdom
Thailand
Guatemala
United States
Rwanda
Chile
South Africa
South Korea
Nigeria
Mexico
Libya
Italy
Burundi
Hong Kong
Mainland China
Macau
Republic Of Sudan
Malaysia
Philippines
Canada
Indonesia
Spain
Vietnam
Egypt
Lebanon
India
Singapore
0 1 2 3 4 5
Online shopping
Brazil
Thailand
United Kingdom
Guatemala
Nigeria
United States
Hong Kong
Malaysia
Italy
Mexico
Saudi Arabia
Philippines
South Africa
Mainland China
South Korea
Macau
Chile
Australia
Rwanda
Republic Of Sudan
Lebanon
Indonesia
Libya
Burundi
Vietnam
Egypt
Singapore
Canada
India
Spain
0 1 2 3 4 5
Food delivery

h. Weighted mean of preference for future preparations by region.

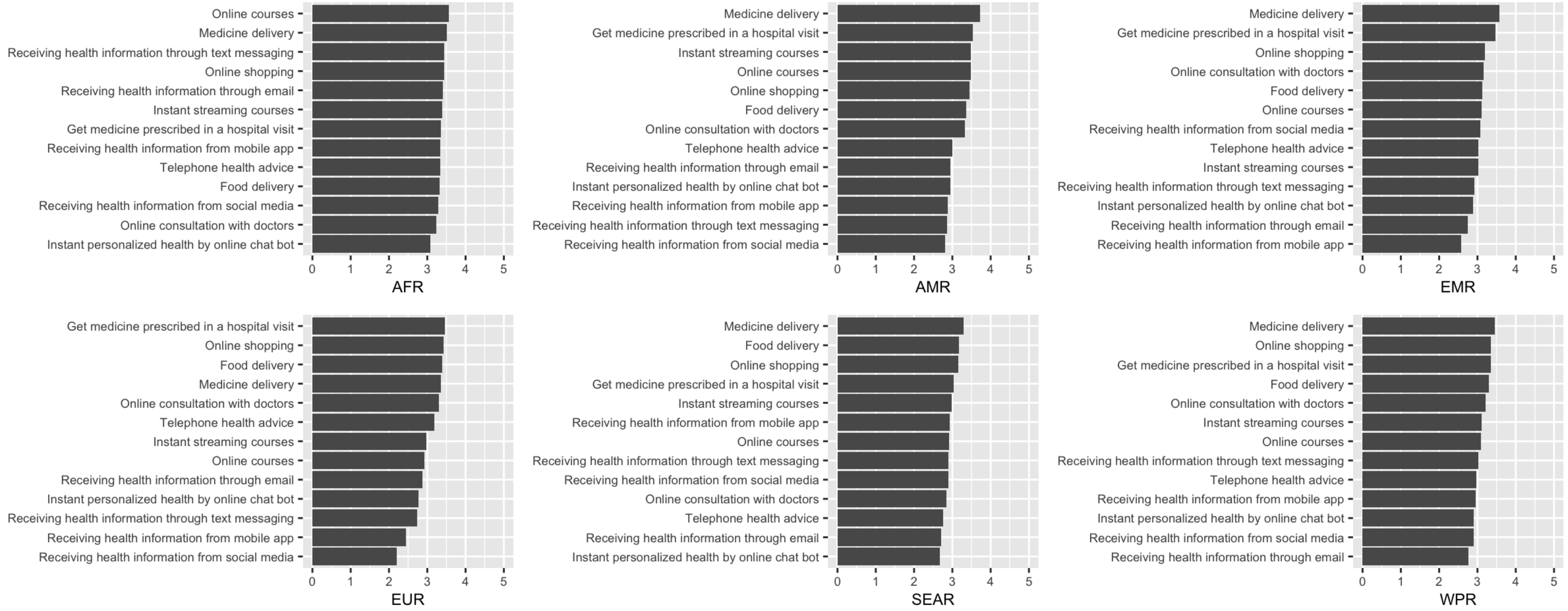

i. Weighted mean of preference for future preparations by economic development level.

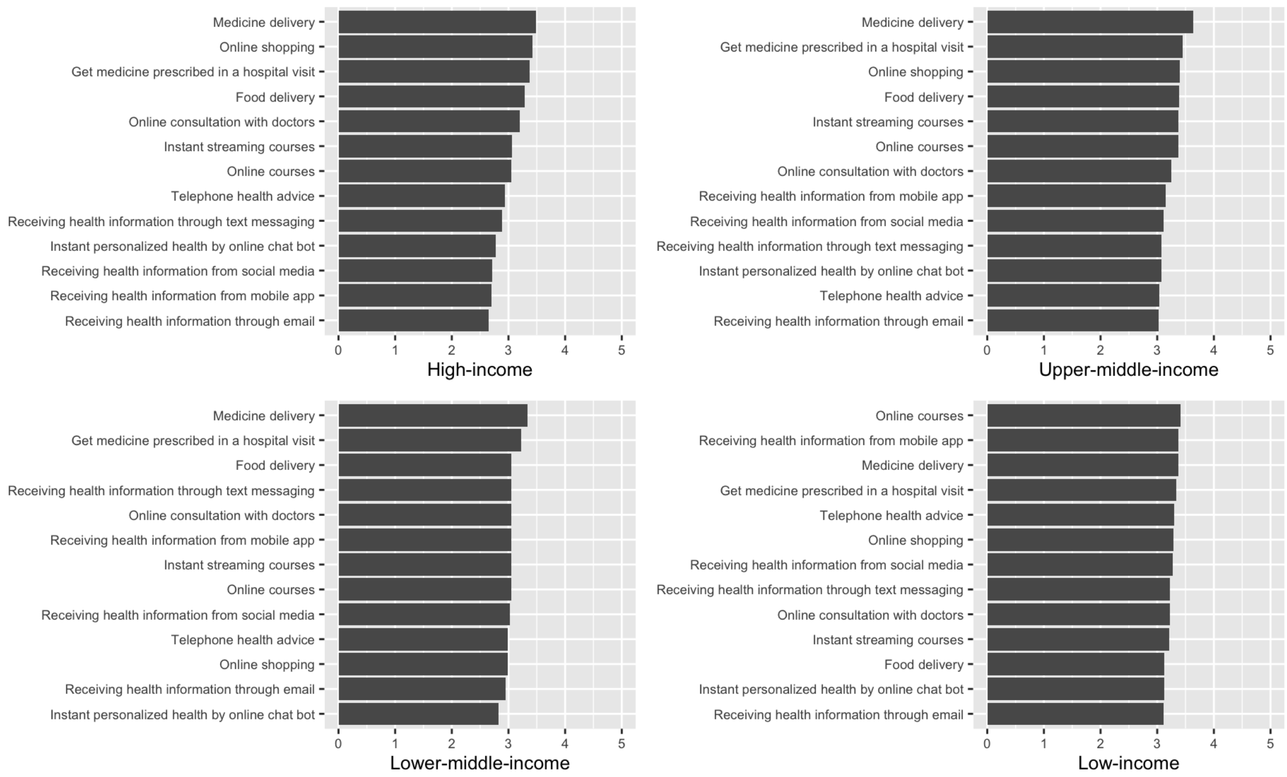

j. Weighted mean of preference for future preparations by COVID-19 severity level.

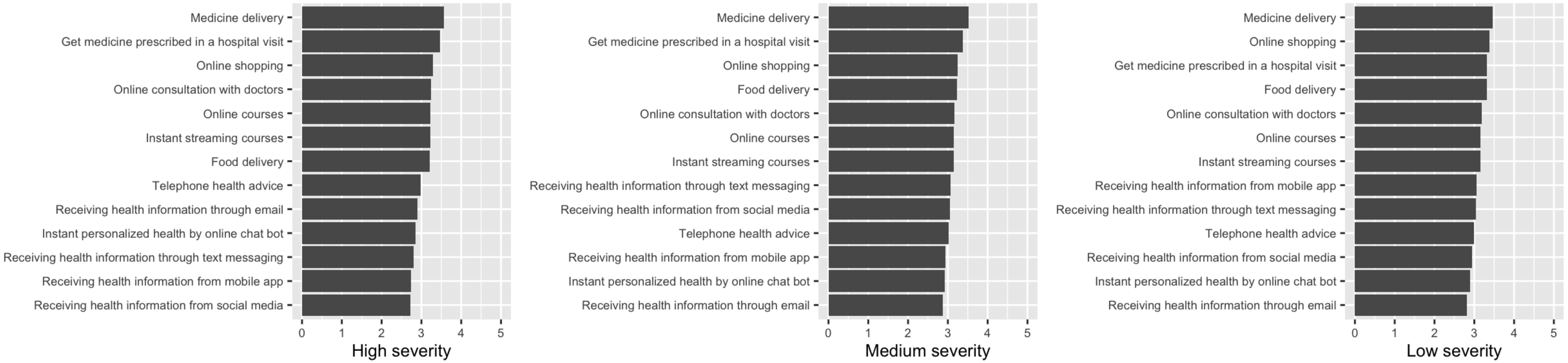

Supplement: Online Supplementary Document [file jogh-13-06031-s001.pdf]
